# Public finance of universal routine childhood immunization in India: district-level cost estimates

**DOI:** 10.1093/heapol/czab114

**Published:** 2021-09-14

**Authors:** Emily Schueller, Arindam Nandi, Amit Summan, Susmita Chatterjee, Arindam Ray, Pradeep Haldar, Ramanan Laxminarayan

**Affiliations:** Center for Disease Dynamics, Economics & Policy, 5636 Connecticut Ave NW, PO Box 42735, Washington, DC 20015, USA; The Population Council, 1 Dag Hammarskjold Plaza, New York, NY 10017, USA; Center for Disease Dynamics, Economics & Policy, 5636 Connecticut Ave NW, PO Box 42735, Washington, DC 20015, USA; The George Institute for Global Health, 311-312, Third Floor, Elegance Tower, Plot No. 8, Jasola District Centre, New Delhi, India; University of New South Wales, Sydney NSW 2052, Australia; Prasanna School of Public Health, Manipal Academy of Higher Education, Madhav Nagar, Eshwar Nagar, Manipal, Karnataka 576104, India; Bill & Melinda Gates Foundation, India Country Office, Capital Court, 5th Floor, Olof Palme Marg, Munirka, New Delhi, Delhi 110067, India; Ministry of Health and Family Welfare, Government of India, Nirman Bhawan, New Delhi 110011, India; Center for Disease Dynamics, Economics & Policy, B-25, 3rd Floor, Lajpat Nagar 2, Lala Lajpat Rai Marg, New Delhi 110024, India; High Meadows Environmental Institute, Guyot Hall, Room 129, Princeton University, Princeton, NJ 08544, USA

**Keywords:** India, UIP, Universal Immunization Programme, cMYP

## Abstract

India’s Universal Immunization Programme (UIP) is among the largest routine childhood vaccination programmes in the world. However, only an estimated 65% of Indian children under the age 2 years were fully vaccinated in 2019. We estimated the cost of raising childhood vaccination coverage to a minimum of 90% in each district in India. We obtained vaccine price data from India’s comprehensive multi-year strategic plan for immunization. Cost of vaccine delivery by district was derived from a 2018 field study in 24 districts. We used propensity score matching methods to match the remaining Indian districts with these 24, based on indicators from the National Family Health Survey (2015–16). We assumed the same unit cost of vaccine delivery in matched pair districts and estimated the total and incremental cost of providing routine vaccines to 90% of the current cohort of children in each district. The estimated national cost of providing basic vaccinations—one dose each of Bacillus Calmette–Guerin (BCG) and measles vaccines, and three doses each of oral polio (OPV) and diphtheria, pertussis and tetanus vaccines—was $784.91 million (2020 US$). Considering all childhood vaccines included in UIP during 2018–22 (one dose each of BCG, hepatitis B and measles–rubella; four doses of OPV; two doses of inactivated polio; and three doses each of rotavirus, pneumococcal and pentavalent vaccines), the estimated national cost of vaccines and delivery to 90% of target children in each district was $1.73 billion. The 2018 UIP budget for vaccinating children, pregnant women and adults was $1.17 billion (2020 US$). In comparison, $1.73 billion would be needed to vaccinate 90% of children in all Indian districts with the recommended schedule of routine childhood vaccines. Additional costs for infrastructural investments and communication activities, not incorporated in this study, may also be necessary.

Key messages

- While substantial progress toward universal child immunization in India has been made since the advent of the Universal Immunization Programme (UIP) in 1985, vaccination rates vary greatly among Indian districts. There is also heterogeneity in the cost of vaccination delivery between Indian districts and states.
- The estimated national cost of providing basic vaccinations was 784.91 million US$ in 2020. The estimated national cost of providing all vaccinations included in the Government of India’s schedule of routine childhood immunization was $1.73 billion US$.
- To achieve universal immunization, the budget for India’s UIP should be increased from its 2020 level of 1.58 billion US$. Variation in district- and state-level costs should be taken into consideration when considering expenditure needed to increase vaccination coverage in underserved areas.

## Introduction

India’s Universal Immunization Programme (UIP) began in 1985 with the goal of vaccinating all pregnant women and 85% of infants against six vaccine-preventable diseases (VPDs) by 1990 (Lahariya, 2014). The programme was expanded in 2011–17 with the introduction of *Haemophilus influenzae* type b (Hib), rotavirus (RVV), measles–rubella (MR), inactivated polio vaccine (IPV) and pneumococcal conjugate (PCV) vaccines. As of 2018, UIP included the following childhood vaccines: oral polio vaccine (OPV), diphtheria–pertussis–tetanus conjugate vaccine (DPTCV), Bacillus Calmette–Guérin (BCG), measles conjugate vaccine (MCV), hepatitis B, pentavalent (DPTCV, hepatitis B and Hib), IPV, RVV, MR, PCV and, in endemic areas, Japanese encephalitis (Ministry of Health and Family Welfare, Government of India, 2017). Vaccines included in UIP are provided by the Indian central government at no cost to the consumer.

Although UIP is among the largest childhood vaccination programmes in the world, with a target population of ∼26 million children each year, large gaps in vaccination coverage persist. India’s full immunization coverage rate (defined as the proportion of 12- to 23-month-old children who have received one dose each of the BCG and measles vaccines and three doses each of OPV and DPTCV) was estimated at 65% in 2019 (UNICEF, 2020), and the number of children who received three doses of DPTCV (another commonly used measure of full vaccination status) was estimated at 91% (World Health Organization, 2019). National coverage rates of individual vaccines were higher, at 92% for BCG, 90% for three doses of OPV, 91% for three doses of DPTCV and 95% for one dose of MCV (World Health Organization, 2019). Birth doses of OPV, DPTCV and BCG had the highest coverage rates, indicating that attrition remains a serious barrier to achieving full immunization coverage. Data from the 2015–16 National Family Health Survey (NFHS-4)—the most recent source of subnational data from all states—showed large variations across regions, with full immunization rates of 35.4% in the state of Nagaland to 89% in the state of Punjab (International Institute for Population Sciences, 2017).

Gaps in vaccination coverage likely contribute to the continued burden of VPDs in India. An estimated 1.2 million Indian children under the age of 5 years die every year, the highest in the world (Liu *et al.*, 2019). In 2015, VPDs such as pneumonia, diarrhoeal diseases, measles and meningitis accounted for >400 000 under-five deaths in India (Fadel *et al.*, 2017). Under-five mortality varies greatly within the country: in 2015, the northeastern states saw an estimated 39.6 deaths per 1000 children aged 1–59 months, compared with 9.97 deaths per 1000 in the southern states (Liu *et al.*, 2019).

To address the persistently low vaccination rates in rural and marginalized communities, the Indian government launched the special immunization campaigns of Mission Indradhanush in 2014 and Intensified Mission Indradhanush in 2017. These programmes redirected resources to districts and urban areas with historically low vaccination rates to vaccinate children who were not reached by routine immunization services (Clarke-Deelder *et al.*, 2021). Surveys were conducted to identify children who had missed doses of routine vaccines, and vaccination sessions were planned to accommodate the families of these children. Mobile teams were formed to provide vaccination sessions to children in remote areas of targeted districts. Analyses of these programmes indicate that MI increased the full immunization coverage rate in targeted districts by 27% (Summan *et al.*, 2021), and IMI increased full immunization coverage by 10.6–18.5% (Gurnani *et al.*, 2018; Clarke-Deelder *et al.*, 2021), but this effect did not persist 8 months after implementation (Clarke-Deelder *et al.*, 2021).

An assessment of the cost of full vaccination coverage in India, especially at the district and state levels, is needed to inform UIP budgeting and operations. Previous UIP budgets were based on the assumption that the average cost to vaccinate an Indian child would be constant over time and geography, considering only the national average expenditure on vaccines, personnel, equipment and other expenses (Chatterjee *et al.*, 2016; 2018a). The 2013–17 comprehensive multiyear plan (cMYP) assumed an average cost of $25 (2017 US$) to immunize each Indian child with BCG, hepatitis B, OPV, DPTCV or pentavalent, and MCV vaccines, not taking into account regional or local variation in the cost of delivery (Ministry of Health and Family Welfare, 2014). A field study of the public cost of delivering routine vaccines in 24 districts across seven Indian states during 2013–14 found substantial variation in average cost per DPTCV3-immunized child and per fully immunized child (Chatterjee *et al.*, 2018a). These estimates were incorporated into budget estimates for the 2018–22 cMYP (Ministry of Health and Family Welfare, Government of India, 2017).

To the best of our knowledge, this is the first study to estimate the district-level aggregate cost of universal childhood vaccination coverage in India. We used data from the 24-district field study (Chatterjee *et al.*, 2018a) and other sources to estimate the cost of 90% full-immunization coverage among children in each Indian district. We also estimated the cost of 90% coverage of new vaccines—RVV, PCV, pentavalent, IPV and MR, with pentavalent and MR replacing DPTCV and MCV, respectively.

## Methods

### Data

We used data from the fourth round of the NFHS-4, which was conducted in 2015–16 and represents the most recent data on childhood immunization in all Indian states and districts (International Institute for Population Sciences, 2017). NFHS-4 collected data on a variety of health, educational, socioeconomic and other metrics from a representative sample of 723 875 women from 601 509 households in all Indian states and territories. The survey covered all 640 districts of India at the time of the survey in 2015–16. We matched newer districts that were created after the survey and the new union territory of Ladakh (created from Jammu and Kashmir in 2019) retrospectively with NFHS-4 district for this analysis. The fifth round of the survey (NFHS-5) is being completed in 2021, and data are not yet available for all states and districts in India.

District-wise vaccination rates for BCG, OPV, DPTCV, MCV and hepatitis B were constructed from these data as the proportion of 12- to 23-month-old children whose mothers or vaccination cards indicated they had received each vaccine. Full immunization was defined as the receipt of all vaccines in the Expanded Programme on Immunization (EPI) of the World Health Organization (WHO): one dose of the BCG and MCV vaccines, three doses of DPTCV and at least three doses of OPV. Although DPTCV has been recently replaced by the pentavalent vaccine, we included DPTCV in our analysis of EPI vaccines to demonstrate the cost of achieving full immunization coverage under the WHO definition (Ministry of Health and Family Welfare, Government of India, 2017). The rollout of the pentavalent vaccine began in selected states prior to NFHS-4 but it was uneven, and NFHS-4 collected data on DPTCV vaccination rather than vaccination with the pentavalent vaccine. We assumed that only DPTCV and not pentavalent vaccine was given in our baseline of already-vaccinated children. Additionally, we assumed zero percent coverage of PCV and RVV, which have recently been introduced into UIP for all Indian states.

Using NFHS-4 data, we estimated the size of the 12- to 23-month-old age cohort in each district as a proportion of the district and national populations. This relative cohort size was used in conjunction with 2020 national population estimates from the United Nations Population Division to estimate the number of children under the age 2 years in each district in 2020 (United Nations, Department of Economic and Social Affairs, Population Division, 2019).

Vaccination delivery cost estimates per dose and per fully immunized child were obtained from a field study (Chatterjee *et al.*, 2018a) that covered a representative sample of 255 primary, secondary and tertiary public health facilities across 24 districts in Bihar, Gujarat, Kerala, Meghalaya, Punjab, Uttar Pradesh and West Bengal. Microcost data on vaccines and supplies, personnel, training, transport, overhead expenses and capital expenditures, such as cold chain infrastructure and vehicles, were collected during 2013–14 from each facility. These data were used to estimate the cost per fully immunized child and cost per dose of delivering each vaccine (Chatterjee *et al.*, 2018a). The costs of social mobilization, communication and various demand-side activities were outside the scope of this study, as district-level unit costs of these activities have not been estimated.

We obtained prices for individual vaccines (BCG, DPTCV, OPV, MCV, hepatitis B, RVV, PCV, IPV, MR and pentavalent vaccines) from the 2018–22 cMYP (Ministry of Health and Family Welfare, Government of India, 2017). We also obtained wastage rates for each vaccine from cMYP 2018–22 (Ministry of Health and Family Welfare, Government of India, 2017) and assumed these to be uniform throughout India. Costs were converted to January 2020 INR using consumer price index data (Organization for Economic Co-operation and Development, 2020) and to USD using the International Monetary Fund’s reported exchange rate (US$1 = INR 71.5) on 31 January 2020 (International Monetary Fund, 2020).

### Statistical analysis

We used propensity score matching to pair 616 districts for which vaccine delivery unit costs were not available with one of the 24 districts covered under an earlier field study (Rosenbaum and Rubin, 1983; Heckman *et al.*, 1997; Dehejia and Wahba, 1999; 2002; Chatterjee *et al.*, 2018a). In observational studies, matching based on propensity scores is a widely used technique for reducing potential differences in the characteristics of two groups (e.g. intervention and control groups in a cohort study), which might affect the outcome. Matching over the entire joint distribution of characteristics (or covariates) could lead to the so-called dimensionality problem where some subgroups of the data may have very few or no observations. For example, a within-state matching of Chatterjee *et al.*’s (2018a) study districts with the remaining districts would not be possible for states not covered in the study. Propensity score matching reduces the dimension from many covariates to a single indicator—the predicted probability of intervention assignment [in our case, inclusion in the Chatterjee *et al.*’s (2018a) study]. The predicted probability—known as the propensity score—can be used to match districts in the Chatterjee *et al.*’s (2018a) study to the remaining districts of India, which were observationally similar but not included in the study.

First, we created a binary indicator to identify districts in the field study. This indicator was then regressed, using a probit model, on a set of district-level indicators: average household size, age of the household head and proportions of households from rural areas, those belonging to socioeconomically disadvantaged groups (Scheduled Caste, Scheduled Tribe, and Other Backwards Classes), religion (Hindu, Muslim, Christian and Sikh), those without access to sanitation and those with a female household head. Household standard of living was captured through quintiles of a composite national index of ownership of assets such as TV, radio, bicycle and car (Pollitt *et al.*, 1993; Filmer and Pritchett, 2001). Covariates also included average age of the district population, proportion of women in the district, average years of schooling completed by mothers of under-five children, proportion of under-two children who were fully immunized and proportion of children who received most vaccines in a government facility. For each district not included in Chatterjee *et al.*’s (2018a) study, we used the predicted probability from this regression (propensity score) to match it with the district in Chatterjee *et al.*’s (2018a) study that had the closest propensity score (one-to-one nearest neighbour matching with replacement). After matching, we assumed that the unit cost of delivering vaccines in that district was the same as that in its matched counterpart from Chatterjee *et al.*’s (2018a) study.

We estimated four cost indicators at the district and state levels for 2020: (1) the cost of delivering only vaccines (excluding all other supplies, personnel and equipment) to 90% of 12- to 23-month-old children (target children); (2) the incremental cost of delivering vaccines to undervaccinated children, where the rate of undervaccination was defined as the difference between 90% coverage and the district’s vaccination coverage level for a given vaccine; (3) the full cost (inclusive of supplies, personnel, etc.) of vaccinating 90% of the target children in each district and (4) the incremental cost of fully vaccinating the undervaccinated children in each district.

We estimated the four cost indicators for the EPI vaccines (BCG, DPTCV, MCV and OPV). In additional analyses, we estimated these indicators after including RVV and PCV (i.e. those vaccines currently being rolled out nationwide) along with the EPI vaccines. Finally, we computed a third set of cost estimates by including the full set of vaccines that were due to be included under UIP nationwide by the end of 2019 (cMYP 2018–22) (Ministry of Health and Family Welfare, Government of India, 2017): one dose of BCG, four doses of OPV, one dose of hepatitis B, three doses of pentavalent, two doses of IPV, three doses of RVV, three doses of PCV and one dose of MR vaccine (Table 1). All vaccine prices paid by UIP were taken from cMYP 2018–22 (Ministry of Health and Family Welfare, Government of India, 2017) and converted to 2020 US$. In calculating the costs of providing only vaccines, we included the cost of each vaccine and its wastage rate.

**Table 1.** Vaccination scenarios

EPI vaccines only	EPI vaccines plus RVV and PCV	cMYP 2018–22 schedule vaccines (new schedule vaccines)
BCG (one dose)	BCG (one dose)	BCG (one dose)
OPV (four doses)	OPV (four doses)	OPV (four doses)
MCV (one dose)	MCV (one dose)	MR vaccine (one dose)
DPTCV (three doses)	DPTCV (three doses)	Pentavalent vaccine (three doses)
	RVV (three doses)	RVV (three doses)
	PCV (three doses)	PCV (three doses)
		IPV (two doses)
		Birth dose hepatitis B (one dose)

Note: BCG = Bacillus Calmette–Guérin; OPV = oral polio vaccine; MCV = measles conjugate vaccine; DPTCV = diphtheria, pertussis, tetanus conjugate vaccine; RVV = rotavirus vaccine; IPV = inactivated polio vaccine; MR = measles–rubella; PCV *= *pneumococcal conjugate vaccine. Pentavalent vaccine includes diphtheria, pertussis, tetanus, hepatitis B and Hib.

Delivery costs (without vaccine price) were taken from the 2018 field study, and they included cost per fully immunized child and cost per dose of vaccine delivered in each district (Chatterjee *et al.*, 2018a). We added delivery cost per fully immunized child to the price of EPI vaccines to construct vaccine and delivery costs for EPI vaccines. For other scenarios, we used delivery cost per dose and individual vaccine prices to calculate the additional cost of vaccinating children with RVV, PCV and IPV. Construction of vaccine only costs is illustrated in Supplementary Appendix Tables A1 and A2, and construction of vaccine and delivery costs is shown in Supplementary Appendix Tables A2 and A3.

For constructing incremental costs under the cMYP 2018–22 schedule (full set of new vaccines), we assumed that there would be no additional delivery cost to replace DPTCV with pentavalent vaccine, i.e. that the delivery cost for a dose of pentavalent vaccine is equivalent to that for DPTCV. Therefore, we took baseline coverage rates for the first, second and third doses of DPTCV from NFHS-4 and assumed the cost difference between pentavalent vaccine and DPTCV would be the only additional cost in replacing DPTCV with pentavalent in the population that has received DPTCV. Similarly, we assumed the only additional cost of replacing MCV with MR vaccine would be the price difference between MCV and MR vaccines, and we used the baseline coverage rate for MCV to calculate incremental costs for MR vaccine. For RVV, PCV and IPV, we assumed a baseline coverage of zero. We did not include Japanese encephalitis vaccine in our analysis because it is given to children in endemic districts only (Muniaraj and Rajamannar, 2019). Additional details of cost construction are presented in Supplementary Appendix Tables A1–A4.

## Results

National cost estimates are presented in Table 2. Detailed state-level estimates are presented in Supplementary Appendix Tables A5 through A8, and district-level estimates are shown in Supplementary Appendix Tables A9 through A11. All cost estimates are presented in 2020 US$.

**Table 2.** Estimated cost (2020 US$) of 90% coverage of vaccines among 12- to 23-month-old Indian children in all districts

Scenario	Cost of 90% coverage, vaccine only	Cost of 90% coverage, vaccine and delivery	Incremental cost, current coverage to 90%, vaccine only	Incremental cost, current coverage to 90%, vaccine and delivery
EPI vaccines only	21 432 537	784 910 044	2 136 163	51 217 951
EPI vaccines, RVV and PCV	354 917 164	1 466 697 524	335 620 801	733 005 419
New cMYP schedule vaccines	502 548 302	1 730 429 635	471 543 840	1 055 728 860

Note: EPI vaccines include one dose each of BCG and MCV, three doses of DPTCV and at least three doses of OPV. New cMYP schedule vaccines include one dose of BCG, four doses of OPV, one dose of hepatitis B, three doses of pentavalent, two doses of IPV, three doses of RVV, three doses of PCV and one dose of measles–rubella vaccine. Current coverage was calculated from NFHS-4 (2015–16) data.

### District vaccination rates

Vaccination rates varied widely across districts, even within the same state. The small northeastern state of Arunachal Pradesh contained the districts with the lowest district-level vaccination rates for BCG (28%), DPTCV1 (25%), DPTCV2 (19%) and measles (19%) vaccines; the large northern state of Uttar Pradesh accounted for districts with the lowest district vaccination rate for DPTCV3 (14%).

### Estimated cost of achieving 90% coverage (vaccines only)

**Figure 1.**
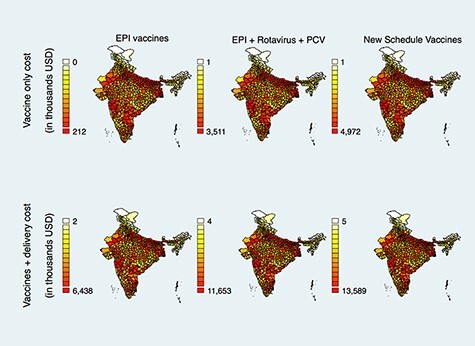
Estimated cost (2020 US$) of 90% coverage of routine vaccines among 12- to 23-month-old Indian children, by district

Note: Estimates are in 2020 US$, with reference to a baseline of no coverage. EPI vaccines include BCG, DPTCV, OPV and MCV. New schedule vaccines include BCG, OPV, IPV, PCV, MR, RVV, Hep B birth dose and Hib-containing pentavalent.

Considering only the price of vaccines (including wastage factors) and excluding personnel, equipment and other expenses associated with their delivery, we estimated the cost of providing EPI (BCG, OPV, DPTCV and measles) vaccines to 90% of target children in every district at a total of $21.43 million (Table 2, Column 2). District-level costs ranged from $56 in Dibang Valley district, Arunachal Pradesh, to $212 024 in Thane district, Maharashtra, and were driven by a large variation in population between districts (Figure 1 and Supplementary Appendix Table A9).

When RVV and PCV were included, the national cost of 90% vaccine coverage was estimated to be $354.92 million, and the cost of 90% vaccine coverage of the full cMYP 2018–22 schedule of vaccines was estimated at $502.55 million (Table 2, Column 2). The cost of 90% vaccine coverage of the full cMYP schedule ranged from $1305 in Dibang Valley district, Arunachal Pradesh, to $4.97 million in Thane district, Maharashtra (Figure 1 and Supplementary Appendix Table A11).

### Incremental cost of achieving 90% coverage (vaccines only)

**Figure 2.**
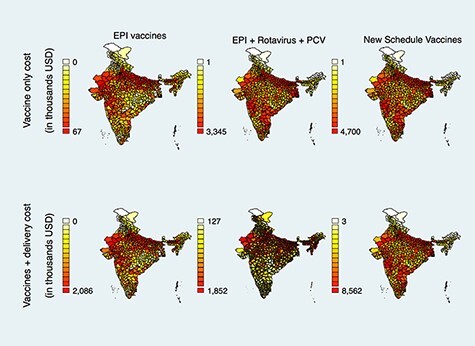
Estimated cost (2020 US$) of 90% incremental coverage of routine vaccines among 12- to 23-month-old Indian children, by district

Note: Estimates are in 2020 US$, with reference to a baseline of no coverage. EPI vaccines include BCG, DPTCV, OPV and MCV. New schedule vaccines include BCG, OPV, IPV, PCV, MR, RVV, Hep B birth dose and Hib-containing pentavalent.

Again considering only the price of vaccines (including wastage factors) and excluding expenses associated with delivery, the national estimated cost of increasing coverage of EPI vaccines from NFHS-4 levels to 90% in each Indian district was $2.14 million (Table 2, Column 4). In 59 districts, vaccination rates for all four vaccines were already above 90%, and there was no unmet need for EPI vaccines. (This does not necessarily translate to a full immunization coverage rate of 90% in these districts, since children may have received some vaccines and not others.) Of districts with vaccination rates below 90%, the estimated cost of vaccines was the highest at $66 961 in Bahraich district, Uttar Pradesh, which had a full immunization coverage rate of 9% and an estimated unmet need for 560 398 doses of BCG, DPTCV, MCV and OPV vaccines (Figure 2 and Supplementary Appendix Table A9). With the addition of RVV and PCV, the national incremental cost from a zero baseline was $335.62 million (Table 2, Column 4). The incremental national cost for the full cMYP 2018–22 schedule of vaccines was $471.54 million (Table 2, Column 4), which ranged from $1232 in Dibang Valley district, Arunachal Pradesh, to $4.7 million in Thane, Maharashtra (Figure 2 and Supplementary Appendix Table A11.

### Estimated cost of achieving 90% coverage (vaccines plus delivery)

The weighted average cost of vaccination per fully immunized child (inclusive of vaccines, supplies, personnel and other expenses) varied widely among districts, from $12.43 to $55.36. Assuming the same unit costs for the matched pairs, the national cost of vaccinating 90% of children with the EPI vaccines was estimated to be $784.91 million (Table 2, Column 3). This cost ranged from $1968 in Upper Siang district, Arunachal Pradesh, which had an average cost of $20.23 to vaccinate a child with EPI vaccines, to $6.44 million in Allahabad (now Prayagraj) district, Uttar Pradesh, with an average unit cost of $53.65 (Figure 1 and Supplementary Appendix Table A9). When we included vaccine and delivery costs for RVV and PCV in the analysis, the national cost of 90% coverage was estimated to be $1.47 billion (Table 2, Column 3). The cost of vaccinating 90% of children in all districts with the cMYP 2018–22 schedule of vaccines was estimated at $1.73 billion nationally (Table 2, Column 3) and ranged from $5311 in Upper Siang district, Arunachal Pradesh, with a cost of $54.58 per child, to $13.59 million in Allahabad (Prayagraj) district, Uttar Pradesh, with a unit cost of $113.23 per child (Figure 1 and Supplementary Appendix Table A11).

### Incremental cost of achieving 90% coverage (vaccines plus delivery)

Weighted average delivery cost per dose ranged from $1.00 in Lucknow district, Uttar Pradesh, to $4.82 in Banaskantha district, Gujarat, in the 2018 field study (Chatterjee *et al.*, 2018a). We estimated the national cost of raising EPI vaccination rates to 90% from the NFHS-4 baseline in every district at $51.22 million (Table 2, Column 5). Excluding the 59 districts that had vaccination rates above 90%, this cost ranged from $11 in North District, Sikkim, to $2.09 million in Bahraich district, Uttar Pradesh (Figure 2 and Supplementary Appendix Table A9). When RVV and PCV were included, the estimated national cost of closing the vaccination gap was $733 million (Table 2, Column 5). The estimated cost of closing the vaccination gap for the cMYP 2018–22 schedule was $1.06 billion (Table 2, Column 5), ranging from $3385 in Dibang Valley district, Arunachal Pradesh, to $8.56 million in Allahabad (Prayagraj) district, Uttar Pradesh (Figure 2 and Supplementary Appendix Table A11).

### Sensitivity analysis: exchange rates and scaling up

The INR–USD exchange rate increased from 71.5 in January 2020 to 75.1 in April 2020 (rate of the last day of the month), coinciding with the coronavirus disease 2019 (COVID-19) pandemic. Using the April rate, national costs (vaccines and delivery) to achieve 90% vaccination coverage in all Indian districts for EPI vaccines only, EPI vaccines with PCV and RVV, and the new schedule of vaccines would be $742.83 million, $1.39 billion and $1.64 billion, respectively. Incremental national costs (vaccines and delivery) to achieve 90% vaccination coverage from NFHS-4 rates for EPI vaccines only, EPI vaccines with PCV and RVV, and the new schedule of vaccines were $48.47 million, $693.71 million and $999.13 million, respectively.

We also analysed the cost of closing the vaccination gap over 4 years, using NFHS-4 coverage rates and 2020 Indian population estimates as a baseline. Using predicted population growth in each Indian state and union territory from 2020 to 2024, we calculated the undervaccinated population for each dose of the EPI and new vaccine schedules. We then estimated the cost of vaccination (vaccines and delivery) to 25% of this population in 2021, 50% in 2022, 75% in 2023 and the entire undervaccinated population in 2024. The total cost from 2021 to 2024 was $131.83 million for EPI vaccines and $2.88 billion for the new schedule vaccines, using the January 2020 exchange rate (Supplementary Appendix Table A8).

## Discussion

Barriers to vaccination vary widely throughout India, and geographic and socioeconomic disparities in vaccination rates persist across localities, even within states (Barman and Partha, 2017). Various studies have found that socioeconomic status, membership in a religious or ethnic minority and living in a rural or slum area are associated with the lower likelihood of vaccination (Singh, 2013; Shrivastwa *et al.*, 2015; Zuhair and Roy, 2017; Francis *et al.*, 2018). Immunization special drives such as Mission Indradhanush and Intensified Mission Indradhanush have succeeded in increasing vaccination rates in underserved districts (Gurnani *et al.*, 2018; Clarke-Deelder *et al.*, 2021; Summan *et al.*, 2021). A study of IMI costs in five Indian states found very high per-dose and per-vaccinated child costs, particularly in the large state of Maharashtra (Chatterjee *et al.*, 2021). Factors driving these high costs compared with routine immunization include large labour costs for healthcare workers, extensive household surveys to locate missed children, local planning meetings to set up temporary vaccination sites and transportation to areas such as islands and plantations that are often inaccessible (Chatterjee *et al.*, 2021).

MI and IMI have demonstrated that vaccination rates can be improved in the short term through concerted effort and coordinated activities (Summan *et al.*, 2021). However, the cost of achieving these short-term goals is high, given the cost of labour and planning to reach missed children, as well as the logistical challenges of reallocating resources across states (Chatterjee *et al.*, 2021; Clarke-Deelder *et al.*, 2021). Achieving and sustaining high levels of immunization coverage will require investments such as the establishment of permanent or seasonal clinics in underserved areas. Cost-effective demand-side activities are also essential, as the high costs of locating children with missed vaccine doses are not feasible in a routine immunization programme. Fixed costs of establishing local clinics, training staff and generating demand are not included in our analysis but will play an essential role in gradually expanding immunization coverage in India.

The vaccines included in the EPI schedule are basic and inexpensive. The total price of vaccine doses needed to vaccinate one child with the entire EPI schedule (one dose of BCG, three doses of DPTCV, four doses of OPV and one dose of MCV) adds up to <$1. Furthermore, 59 districts had already achieved vaccination rates above 90% for these vaccines and had an incremental cost of zero for EPI vaccines. These factors result in a relatively low cost ($2.1 million) to achieve 90% vaccination rates for EPI vaccines in all districts. Comparatively, the newly introduced vaccines PCV and RVV cost $3.10 per dose and $1.19 per dose, respectively, and the UIP schedule necessitates three doses of each vaccine. We assumed a baseline coverage rate of zero for these vaccines as they have been introduced recently. Therefore, the introduction of PCV alone will cost $227 million for the entire target population, and the cost of providing PCV and RVV to 90% of children in all districts constitutes a large majority of the incremental national cost for EPI vaccines, RVV and PCV. In addition to the high costs of RVV and PCV vaccines, the new cMYP schedule has introduced two doses of IPV, which costs $1.51 per dose, and replaces DPTCV and MCV with the more expensive pentavalent and MR vaccines. Delivery costs for the introduction of eight new doses (three doses of RVV, three doses of PCV and two doses of IPV) account for an additional $464 million for the national population. Although expensive, these vaccines are expected to have a large benefit in a country where in 2015, 192 000 children under 5 years of age were estimated to have died of pneumococcal pneumonia (Wahl *et al.*, 2020).

We found substantial variation in district-level costs driven by differences in population, delivery costs and vaccination rates. The population of Indian children aged 0–6 years (2011 census) ranged from 1084 in Dibang Valley district, Arunachal Pradesh, (Directorate of Census Operations, Arunachal Pradesh, 2014) to 1 327 146 in Thane district, Maharashtra (Directorate of Census Operations, Maharashtra, 2014), and these districts had the lowest and highest costs, respectively, in our vaccine-only scenarios. When we also considered the cost of delivery, the varying costs of delivery per fully immunized child and per dose affected the district-level estimates, but the population continued to play a substantial role in variation between districts. Upper Siang district, Arunachal Pradesh, was the second-least populated district and had the lowest costs for vaccines and delivery. However, Allahabad district, Uttar Pradesh, had the highest costs for vaccines and delivery despite its status as the 15th most populated district in the country. This was due to the high per-dose and per-fully-immunized child cost of delivery in the district—although other districts were more populous than Allahabad, low unit costs of vaccine delivery resulted in lower aggregate costs in those districts. This variation highlights the importance of regional and district-level differences in vaccine delivery costs, as they can play a large role in funding needs for large districts.

Total baseline UIP expenditure in 2017–18, inclusive of adult vaccination, supplemental immunization activities and social mobilization, was $1.13 billion (2020 US$), calculated with an assumed national average cost of $25 (2017 US$) per child. Although projected UIP expenditure increased to $1.58 billion for 2020, we found the portion of this expenditure dedicated to routine immunization of children under 12 months ($1.17 billion, 2020 US$) to be insufficient for vaccinating children against the nine VPDs evaluated here. By our estimates, fully vaccinating 90% of children in all Indian districts with BCG, hepatitis B, pentavalent, OPV, IPV, MR, RVV and PCV vaccines will cost ∼ $1.73 billion for the 2020 cohort.

UIP funding from Gavi is projected to fall from 21% of 2017–18 UIP expenditure to <3% of 2022 UIP expenditure. Expenditures on routine child and maternal vaccines (including EPI vaccines; booster doses; tetanus toxoid for pregnant women; Japanese encephalitis vaccine in endemic districts; and PCV, RVV and MR in states where they had been introduced) accounted for $208 million in 2017, of which Gavi and other partners contributed 47% (Ministry of Health and Family Welfare, Government of India, 2017). The high cost of new vaccines such as PCV will necessitate higher spending by the Indian government as partner support is reduced. This is reflected in cMYP 2018–22, which projects a 111% increase in expenditures on vaccines and an 80% increase in total immunization expenditures between 2017 and 2022 (Ministry of Health and Family Welfare, Government of India, 2017).

Our estimates for the cost of vaccinating 90% of target children are higher than projected government expenditure because we used district-level cost estimates rather than national or state estimates. Whereas the national per-dose cost of vaccine delivery was $2.37 (2020 US$), district unit costs ranged from $0.97 in Lucknow, Uttar Pradesh, to $4.73 in Banaskantha, Gujarat (Chatterjee *et al.*, 2018a). Although only one-third of the 24 districts with cost estimates available had per-dose delivery costs above the national average, more than half of all districts were assigned district costs above the national average through propensity score matching. The large proportion of matched districts with high delivery costs suggests that previous national estimates of vaccination costs may underestimate the true cost of vaccine delivery in many districts.

Our analysis was limited by the availability of data on both vaccination rates and the variable cost of vaccination. District-level estimates of vaccination coverage in India are based on large and standardized household surveys such as the NFHS, which are available at 5-yearly or longer intervals. Vaccination policies and programmes in India have evolved since the NFHS-4 survey in 2015–16, with the introduction of several new vaccines and the introduction of Mission Indradhanush and Intensified Mission Indradhanush (Ministry of Health and Family Welfare, Government of India, 2017). The fifth round of NFHS, planned for 2018–19, has been delayed due to the COVID-19 pandemic but is likely to provide a more accurate estimate of the current state of the child vaccination coverage in India. At present, NFHS-5 summary statistics on immunization coverage have been released for 22 states and Union Territories and are therefore not available for an aggregate analysis of district-level costs in all Indian districts. NFHS-5 summary sheets do not provide data on every dose of vaccine administered and do not provide estimates of children who received one or two doses of OPV, DPTCV, pentavalent vaccine, etc. or estimates of PCV or IPV coverage. Additionally, district-level information on the socioeconomic characteristics we used for matching districts are not yet available from NFHS-5. Given these constraints, NFHS-4 is the most recent available complete data set of vaccination in all Indian districts. Our baseline district-wise vaccination rates could be underestimates because of the potential gains made between 2016 and 2020, as estimated by two recent survey studies of Mission Indradhanush (Pramanik *et al.*, 2016) and Intensified Mission Indradhanush (Gurnani *et al.*, 2018).

Previous studies have estimated the cost of vaccination in one Indian locality or state, presenting estimates that vary substantially based on geography and facility type (Rheingans *et al.*, 2014; Prinja *et al.*, 2017; 2018). The 2018 study by Chatterjee *et al.* used and cited in our paper is the most comprehensive effort to date, estimating vaccination costs across several locations and facility types and stratified according to geographic location and level of development (Chatterjee *et al.*, 2018a). However, those estimates were limited in scope: they included only 7 states and 24 districts from India’s 29 states and 640 districts. Transportation costs, density of immunization facilities, accessibility of healthcare workers and other factors have a large influence on vaccination costs (Chatterjee *et al.*, 2018b), but accurate and representative data for these costs across India are lacking.

Analysis of the determinants of vaccination cost has suggested that the costs of scaling up immunization coverage do not follow a predictable curve and that the marginal cost of increasing coverage may vary based on local factors (Chatterjee *et al.*, 2021). Furthermore, the introduction of new vaccines into UIP will likely necessitate additional costs in the form of social mobilization, advocacy, training of healthcare workers and communication to effectively increase the uptake of the new vaccines. The cost of training has been built into delivery costs in our estimates, but the cost of specialized training and social mobilization for new vaccines specifically has not yet been fully quantified at the subnational level in India. A new study from five Indian states has estimated the cost of communication and social mobilization under the Intensified Mission Indradhanush campaign to range from 12.6% to 35.6% of the total cost (Clarke-Deelder *et al.*, 2021). If we assumed similar additional costs for increasing coverage under the UIP, the national cost of 90% coverage of EPI vaccines (including delivery) would be $883.81 million–$1.06 billion, while 90% coverage of cMYP 2018–22 schedule of vaccines would cost $1.95–$2.35 billion. Additional microcosting studies may be required to gain a more comprehensive national and subnational cost of UIP.

Studies of immunization financing in other low- and middle-income countries (LMICs) show that vaccines constitute at least 40% expenses and variable costs that also include labour and supplies account for up to 98% of immunization costs (Sarker *et al.*, 2018; Sim *et al.*, 2021). However, analyses vary in their inclusion of facility costs, and many programmes rely on temporary immunization facilities rather than healthcare facilities. An analysis of six LMICs found that small-scale vaccination sites had substantial variation in average costs and had higher average costs than large-scale sites, indicating a larger fixed cost (e.g. building health facilities) required for reaching underserved or rural communities (Menzies *et al.*, 2012). Our analysis partially incorporates the additional fixed costs of scaling up routine immunization in areas that may require infrastructural investments. Chatterjee *et al.*’s (2018a) study covered a range of districts that had full immunization rates from below 50% (e.g. in Bihar) to over 90% (e.g. in Punjab). The unit costs from the high-coverage districts in Chatterjee *et al.*’s (2018a) study and their matched counterparts among the remaining districts of India may already reflect past infrastructural investments. Due to the lack of data, we could not model these fixed costs separately in our analysis. Initially, the fixed costs may increase the cost of immunization in underserved areas, although such investments will likely decrease the unit cost of vaccination over the long term. Improvements in digital recordkeeping of vaccine stocks through the Electronic Vaccine Intelligence Network (Ministry of Health and Family Welfare, Government of India, 2018) and other health system–strengthening initiatives will likely reduce marginal costs to some degree, although this may be offset by increased costs of last-mile delivery, as indicated by the high costs of IMI (Chatterjee *et al.*, 2021).

Finally, the COVID-19 pandemic has increased the cost of routine vaccination. WHO-recommended practices for conducting vaccinations during the pandemic include training on infection prevention and control, fewer children per immunization session, screening for possible illness and personal protective equipment (World Health Organization, 2020). Implementing such modifications is estimated to cost $6000–$15 000 per facility for the first year of the pandemic (Portnoy and Resch, 2020). While the long-term impact of the COVID-19 pandemic on immunization programmes is unknown, the combination of high costs for recommended interventions and the large number of children requiring catch-up vaccinations (UNICEF, 2021) will present challenges for national immunization programmes in the coming years. Lessons from IMI may prove useful in increasing catch-up vaccination rates after the COVID-19 pandemic wanes (Gurnani *et al.*, 2018).

## Conclusion

Our study is the first to provide comprehensive national, state and district-level estimates of the government cost of providing a near-universal 90% coverage of child vaccinations in India. We found that the expenditure necessary to achieve this target in all districts ($1.73 billion) exceeds the 2020 budget for India’s immunization programme. Future UIP budgets should consider the high levels of variation in the district-level cost of delivering vaccines.

## Supplementary Material

czab114_Supp

## Data Availability

The data underlying this article are publicly available from the Demographic and Health Surveys (https://dhsprogram.com/data/) and https://dx.doi.org/10.1136/bmjgh-2018-000794.
