# Supplementary material for: Public finance of universal routine childhood immunization in India: district-level cost estimates
Source: Health Policy Plan. 2021 Sep 14;37(2):200–8. doi: 10.1093/heapol/czab114 (PMC8826633; doi:10.1093/heapol/czab114)
Supplement: czab114_Supp [file czab114_supp.zip › Supplementary appendix.docx]

**Supplementary Appendix**

Table A1: Estimated cost of achieving 90% coverage (vaccines only)

|  | Vaccine dose | Number of doses | Wastage factor | Target Population |
| --- | --- | --- | --- | --- |
| EPI Vaccines Only | BCG | 1 | 2 | 90% of children aged 12-23 months by district. |
|  | OPV | 3 | 1.11 |  |
|  | MCV | 1 | 1.33 |  |
|  | DPTCV | 3 | 1.11 |  |
| EPI Vaccines plus RVV and PCV | BCG | 1 | 2 |  |
|  | OPV | 3 | 1.11 |  |
|  | MCV | 1 | 1.33 |  |
|  | DPTCV | 3 | 1.11 |  |
|  | RVV | 3 | 1.33 |  |
|  | PCV | 3 | 1.11 |  |
| cMYP 2018–2022 Schedule Vaccines (New Schedule Vaccines) | BCG | 1 | 2 |  |
|  | OPV | 4 | 1.11 |  |
|  | MR vaccine | 1 | 1.33 |  |
|  | Pentavalent vaccine | 3 | 1.11 |  |
|  | RVV | 3 | 1.33 |  |
|  | PCV | 3 | 1.11 |  |
|  | IPV | 2 | 1.11 |  |
|  | Birth dose hepatitis B | 1 | 1.11 |  |

Note: Vaccine prices, number of doses, and wastage factors were obtained from the Government of India Ministry of Health and Family Welfare 2018-2022 Comprehensive Multi-Year Plan (Ministry of Health and Family Welfare, Government of India, 2018). The target population was calculated using National Family Health Survey (NFHS-4) (International Institute for Population Sciences and ICF, 2017) and United Nations Populations Division estimates (United Nations, 2020). District-wise costs for each vaccine were calculated by multiplying the price of the vaccine, the number of doses required, the wastage factor, and the population of 12-23-month-old children in each district (represented by columns). National costs for each vaccination scenario were calculated by adding the vaccine costs for each district

Table A2: Incremental cost of achieving 90% coverage (vaccines only)

|  | Vaccine dose | Wastage factor | Proportion of Undervaccinated Children | Total Population |
| --- | --- | --- | --- | --- |
| EPI Vaccines Only | BCG | 2 | 90% - district-specific coverage rate for BCG | Population of children aged 12-23 months by district. |
|  | OPV | 1.11 | 90% - district-specific coverage rate for OPV0 |  |
|  |  |  | 90% - district-specific coverage rate for OPV1 |  |
|  |  |  | 90% - district-specific coverage rate for OPV2 |  |
|  | MCV | 1.33 | 90% - district-specific coverage rate for MCV1 |  |
|  | DPTCV | 1.11 | 90% - district-specific coverage rate for DPTCV1 |  |
|  |  |  | 90% - district-specific coverage rate for DPTCV2 |  |
|  |  |  | 90% - district-specific coverage rate for DPTCV3 |  |
| EPI Vaccines plus RVV and PCV | BCG | 2 | 90% - district-specific coverage rate for BCG |  |
|  | OPV | 1.11 | 90% - district-specific coverage rate for OPV0 |  |
|  |  |  | 90% - district-specific coverage rate for OPV1 |  |
|  |  |  | 90% - district-specific coverage rate for OPV2 |  |
|  | MCV | 1.33 | 90% - district-specific coverage rate for MCV |  |
|  | DPTCV | 1.11 | 90% - district-specific coverage rate for DPTCV1 |  |
|  |  |  | 90% - district-specific coverage rate for DPTCV2 |  |
|  |  |  | 90% - district-specific coverage rate for DPTCV3 |  |
|  | RVV | 1.33 | 90% (baseline zero coverage for RVV1) |  |
|  |  |  | 90% (baseline zero coverage for RVV2) |  |
|  |  |  | 90% (baseline zero coverage for RVV3) |  |
|  | PCV | 1.11 | 90% (baseline zero coverage for PCV1) |  |
|  |  |  | 90% (baseline zero coverage for PCV2) |  |
|  |  |  | 90% (baseline zero coverage for PCV3) |  |
| cMYP 2018–2022 Schedule Vaccines (New Schedule Vaccines) | BCG | 2 | 90% - district-specific coverage rate for BCG |  |
|  | OPV | 1.11 | 90% - district-specific coverage rate for OPV0 |  |
|  |  |  | 90% - district-specific coverage rate for OPV1 |  |
|  |  |  | 90% - district-specific coverage rate for OPV2 |  |
|  |  |  | 90% - district-specific coverage rate for OPV3 |  |
|  | MR | 1.33 | 90% - district-specific coverage rate for MCV |  |
|  | Replacing MCV with MR | 1.33 | District-specific coverage rate for MCV* |  |
|  | Pentavalent | 1.11 | 90% - district-specific coverage rate for DPTCV1 |  |
|  |  |  | 90% - district-specific coverage rate for DPTCV2 |  |
|  |  |  | 90% - district-specific coverage rate for DPTCV3 |  |
|  | Replacing DPT with pentavalent |  | District-specific coverage rate for DPT1* |  |
|  |  |  | District-specific coverage rate for DPT2* |  |
|  |  |  | District-specific coverage rate for DPT3* |  |
|  | RVV | 1.33 | 90% (baseline zero coverage for RVV1) |  |
|  |  |  | 90% (baseline zero coverage for RVV2) |  |
|  |  |  | 90% (baseline zero coverage for RVV3) |  |
|  | PCV | 1.11 | 90% (baseline zero coverage for PCV1) |  |
|  |  |  | 90% (baseline zero coverage for PCV2) |  |
|  |  |  | 90% (baseline zero coverage for PCV3) |  |
|  | IPV | 1.11 | 90% (baseline zero coverage for IPV1) |  |
|  |  |  | 90% (baseline zero coverage for IPV2) |  |
|  | Birth dose hepatitis B | 1.11 | 90% - district-specific coverage rate for hepB0 |  |

Note: Vaccine prices, number of doses, and wastage factors were obtained from the Government of India Ministry of Health and Family Welfare 2018-2022 Comprehensive Multi-Year Plan (Ministry of Health and Family Welfare, Government of India, 2018). The target population was calculated using National Family Health Survey (NFHS-4) (International Institute for Population Sciences and ICF, 2017) and United Nations Populations Division estimates (United Nations, 2020). District-specific coverage rates of individual vaccines were obtained from NFHS-4 data (International Institute for Population Sciences and ICF, 2017). District-wise costs for each vaccine dose were calculated by multiplying the price of the vaccine, the wastage factor, the district-specific coverage rate for the dose subtracted from 90%, and the population of 12-23-month-old children in each district (represented by columns). Newly introduced vaccines were assumed to have a baseline of zero, and coverage rates for DPTCV and MCV were used to calculate the cost of replacing these vaccines with pentavalent vaccine and MR vaccine, respectively. National costs for each vaccination scenario were calculated by adding the vaccine costs for each district.

Table A3: Estimated cost of achieving 90% coverage (vaccines and delivery)

|  | Cost of Vaccines | | | | Cost of Delivery | | |
| --- | --- | --- | --- | --- | --- | --- | --- |
|  | Vaccine dose | Number of doses | Wastage factor | Target Population | Delivery Cost | Number of doses | Target Population |
| EPI Vaccines Only | BCG | 1 | 2 | 90% of children aged 12-23 months by district. | District-wise cost of delivery for fully-immunized child | 1 | 90% of children aged 12-23 months by district. |
|  | OPV | 3 | 1.11 |  |  | 3 |  |
|  | MCV | 1 | 1.33 |  |  | 1 |  |
|  | DPTCV | 3 | 1.11 |  |  | 3 |  |
| EPI Vaccines plus RVV and PCV | BCG | 1 | 2 |  | District-wise cost of delivery for fully-immunized child | 1 |  |
|  | OPV | 3 | 1.11 |  |  | 3 |  |
|  | MCV | 1 | 1.33 |  |  | 1 |  |
|  | DPTCV | 3 | 1.11 |  |  | 3 |  |
|  | RVV | 3 | 1.33 |  | District-wise cost of delivery for one dose of vaccine | 3 |  |
|  | PCV | 3 | 1.11 |  |  | 3 |  |
| cMYP 2018–2022 Schedule Vaccines (New Schedule Vaccines) | BCG | 1 | 2 |  | District-wise cost of delivery for fully-immunized child | 1 |  |
|  | OPV | 4 | 1.11 |  |  | 4 |  |
|  | MR vaccine | 1 | 1.33 |  |  | 1 |  |
|  | Pentavalent vaccine | 3 | 1.11 |  |  | 3 |  |
|  | RVV | 3 | 1.33 |  | District-wise cost of delivery for one dose of vaccine | 3 |  |
|  | PCV | 3 | 1.11 |  |  | 3 |  |
|  | IPV | 2 | 1.11 |  |  | 2 |  |
|  | Birth dose hepatitis B | 1 | 1.11 |  |  | 1 |  |

Note: Vaccine prices, number of doses, and wastage factors were obtained from the Government of India Ministry of Health and Family Welfare 2018-2022 Comprehensive Multi-Year Plan (Ministry of Health and Family Welfare, Government of India, 2018). The target population was calculated using National Family Health Survey (NFHS-4) (International Institute for Population Sciences and ICF, 2017) and United Nations Populations Division estimates (United Nations, 2020). Cost of delivery per dose and per fully-vaccinated child were taken from a costing study of immunization services in India (Chatterjee *et al.*, 2018) and matched to districts based on the result of propensity score matching. District-wise vaccine costs were calculated by multiplying the price of the vaccine, the number of doses required, the wastage factor, and the population of 12-23-month-old children in each district (represented by the columns under “Cost of vaccines”). District-wise delivery costs were calculated by multiplying district-wise cost of vaccine delivery, the number of doses required, and 90% of the population of 12-23-month-old children in each district (represented by the columns under “Cost of delivery”). National costs for each vaccination scenario were calculated by adding vaccine and delivery costs for each district.

Table A4: Incremental cost of achieving 90% coverage (vaccines and delivery)

|  | Cost of Vaccines | | |  | Cost of Delivery | | |
| --- | --- | --- | --- | --- | --- | --- | --- |
|  | Vaccine dose cost | Wastage factor | Proportion of Undervaccinated Children | Target Population | Delivery Cost | Proportion of Undervaccinated Children | Target Population |
| EPI Vaccines Only | BCG | 2 | 90% - district-specific coverage rate for BCG | Population of children aged 12-23 months by district. | District-wise cost of delivery for one dose of vaccine | 90% - district-specific coverage rate for BCG | Population of children aged 12-23 months by district. |
|  | OPV | 1.11 | 90% - district-specific coverage rate for OPV0 |  |  | 90% - district-specific coverage rate for OPV0 |  |
|  |  |  | 90% - district-specific coverage rate for OPV1 |  |  | 90% - district-specific coverage rate for OPV1 |  |
|  |  |  | 90% - district-specific coverage rate for OPV2 |  |  | 90% - district-specific coverage rate for OPV2 |  |
|  | MCV | 1.33 | 90% - district-specific coverage rate for MCV1 |  |  | 90% - district-specific coverage rate for MCV1 |  |
|  | DPTCV | 1.11 | 90% - district-specific coverage rate for DPTCV1 |  |  | 90% - district-specific coverage rate for DPTCV1 |  |
|  |  |  | 90% - district-specific coverage rate for DPTCV2 |  |  | 90% - district-specific coverage rate for DPTCV2 |  |
|  |  |  | 90% - district-specific coverage rate for DPTCV3 |  |  | 90% - district-specific coverage rate for DPTCV3 |  |
| EPI Vaccines plus RVV and PCV | BCG | 2 | 90% - district-specific coverage rate for BCG |  |  | 90% - district-specific coverage rate for BCG |  |
|  | OPV | 1.11 | 90% - district-specific coverage rate for OPV0 |  |  | 90% - district-specific coverage rate for OPV0 |  |
|  |  |  | 90% - district-specific coverage rate for OPV1 |  |  | 90% - district-specific coverage rate for OPV1 |  |
|  |  |  | 90% - district-specific coverage rate for OPV2 |  |  | 90% - district-specific coverage rate for OPV2 |  |
|  | MCV | 1.33 | 90% - district-specific coverage rate for MCV |  |  | 90% - district-specific coverage rate for MCV |  |
|  | DPTCV | 1.11 | 90% - district-specific coverage rate for DPTCV1 |  |  | 90% - district-specific coverage rate for DPTCV1 |  |
|  |  |  | 90% - district-specific coverage rate for DPTCV2 |  |  | 90% - district-specific coverage rate for DPTCV2 |  |
|  |  |  | 90% - district-specific coverage rate for DPTCV3 |  |  | 90% - district-specific coverage rate for DPTCV3 |  |
|  | RVV | 1.33 | 90% (baseline zero coverage for RVV1) |  |  | 90% (baseline zero coverage for RVV1) |  |
|  |  |  | 90% (baseline zero coverage for RVV2) |  |  | 90% (baseline zero coverage for RVV2) |  |
|  |  |  | 90% (baseline zero coverage for RVV3) |  |  | 90% (baseline zero coverage for RVV3) |  |
|  | PCV | 1.11 | 90% (baseline zero coverage for PCV1) |  |  | 90% (baseline zero coverage for PCV1) |  |
|  |  |  | 90% (baseline zero coverage for PCV2) |  |  | 90% (baseline zero coverage for PCV2) |  |
|  |  |  | 90% (baseline zero coverage for PCV3) |  |  | 90% (baseline zero coverage for PCV3) |  |
| cMYP 2018–2022 Schedule Vaccines (New Schedule Vaccines) | BCG | 2 | 90% - district-specific coverage rate for BCG |  |  | 90% - district-specific coverage rate for BCG |  |
|  | OPV | 1.11 | 90% - district-specific coverage rate for OPV0 |  |  | 90% - district-specific coverage rate for OPV0 |  |
|  |  |  | 90% - district-specific coverage rate for OPV1 |  |  | 90% - district-specific coverage rate for OPV1 |  |
|  |  |  | 90% - district-specific coverage rate for OPV2 |  |  | 90% - district-specific coverage rate for OPV2 |  |
|  |  |  | 90% - district-specific coverage rate for OPV3 |  |  | 90% - district-specific coverage rate for OPV3 |  |
|  | MR | 1.33 | 90% - district-specific coverage rate for MCV |  |  | 90% - district-specific coverage rate for MCV |  |
|  | MR - MCV | 1.33 | District-specific coverage rate for MCV* |  |  | District-specific coverage rate for MCV* |  |
|  | Pentavalent | 1.11 | 90% - district-specific coverage rate for DPTCV1 |  |  | 90% - district-specific coverage rate for DPTCV1 |  |
|  |  |  | 90% - district-specific coverage rate for DPTCV2 |  |  | 90% - district-specific coverage rate for DPTCV2 |  |
|  |  |  | 90% - district-specific coverage rate for DPTCV3 |  |  | 90% - district-specific coverage rate for DPTCV3 |  |
|  | Pentavalent - DPT |  | District-specific coverage rate for DPT1* |  |  | District-specific coverage rate for DPT1* |  |
|  |  |  | District-specific coverage rate for DPT2* |  |  | District-specific coverage rate for DPT2* |  |
|  |  |  | District-specific coverage rate for DPT3* |  |  | District-specific coverage rate for DPT3* |  |
|  | RVV | 1.33 | 90% (baseline zero coverage for RVV1) |  |  | 90% (baseline zero coverage for RVV1) |  |
|  |  |  | 90% (baseline zero coverage for RVV2) |  |  | 90% (baseline zero coverage for RVV2) |  |
|  |  |  | 90% (baseline zero coverage for RVV3) |  |  | 90% (baseline zero coverage for RVV3) |  |
|  | PCV | 1.11 | 90% (baseline zero coverage for PCV1) |  |  | 90% (baseline zero coverage for PCV1) |  |
|  |  |  | 90% (baseline zero coverage for PCV2) |  |  | 90% (baseline zero coverage for PCV2) |  |
|  |  |  | 90% (baseline zero coverage for PCV3) |  |  | 90% (baseline zero coverage for PCV3) |  |
|  | IPV | 1.11 | 90% (baseline zero coverage for IPV1) |  |  | 90% (baseline zero coverage for IPV1) |  |
|  |  |  | 90% (baseline zero coverage for IPV2) |  |  | 90% (baseline zero coverage for IPV2) |  |
|  | Birth dose hepatitis B | 1.11 | 90% - district-specific coverage rate for hepB0 |  |  | 90% - district-specific coverage rate for hepB0 |  |

Note: Vaccine prices, number of doses, and wastage factors were obtained from the Government of India Ministry of Health and Family Welfare 2018-2022 Comprehensive Multi-Year Plan (Ministry of Health and Family Welfare, Government of India, 2018). The target population was calculated using National Family Health Survey (NFHS-4) (International Institute for Population Sciences and ICF, 2017) and United Nations Populations Division estimates (United Nations, 2020). District-specific coverage rates of individual vaccines were obtained from NFHS-4 data (International Institute for Population Sciences and ICF, 2017). Cost of delivery per dose and per fully-vaccinated child were taken from a costing study of immunization services in India (Chatterjee *et al.*, 2018) and matched to districts based on the result of propensity score matching. District-wise vaccine costs were calculated by multiplying the price of the vaccine, the number of doses required, the wastage factor, and the population of 12-23-month-old children in each district (represented by the columns under “Cost of vaccines”). District-wise delivery costs were calculated by multiplying district-wise cost of vaccine delivery, the number of doses required, and 90% of the population of 12-23-month-old children in each district (represented by the columns under “Cost of delivery”). Newly introduced vaccines were assumed to have a baseline of zero, and coverage rates for DPTCV and MCV were used to calculate the cost of replacing these vaccines with pentavalent vaccine and MR vaccine, respectively. National costs for each vaccination scenario were calculated by adding vaccine and delivery costs for each district.

Table A5: Estimated cost (2020 US$) of 90% coverage of EPI vaccines (BCG, DPTCV, OPV, and MCV) among 12- to 23-month-old Indian children, by state

| State | Cost of 90% coverage, vaccine only | Cost of 90% coverage, vaccine and delivery | Incremental cost, current coverage to 90%, vaccine only | Incremental cost, current coverage to 90%, vaccine and delivery |
| --- | --- | --- | --- | --- |
| Andaman and Nicobar Islands | 4,895 | 84,519 | 394 | 3,813 |
| Andhra Pradesh | 742,246 | 27,988,171 | 26,198 | 759,031 |
| Arunachal Pradesh | 16,295 | 754,731 | 5,812 | 173,651 |
| Assam | 518,816 | 17,920,838 | 113,292 | 2,957,167 |
| Bihar | 2,601,114 | 88,590,483 | 260,670 | 5,192,796 |
| Chandigarh | 12,242 | 430,468 | 122 | 2,485 |
| Chhattisgarh | 490,876 | 17,552,306 | 7,235 | 198,015 |
| Dadra and Nagar Haveli | 4,957 | 234,530 | 437 | 14,027 |
| Daman and Diu | 2,298 | 109,138 | 305 | 9,579 |
| Goa | 19,896 | 792,563 | 0 | 0 |
| Gujarat | 860,291 | 32,288,051 | 128,976 | 3,498,070 |
| Haryana | 478,704 | 17,375,201 | 60,659 | 1,785,361 |
| Himachal Pradesh | 99,416 | 3,366,494 | 4,131 | 80,939 |
| Jammu and Kashmir | 189,137 | 6,150,145 | 7,419 | 165,938 |
| Jharkhand | 667,760 | 29,668,645 | 53,575 | 1,594,771 |
| Karnataka | 912,608 | 32,938,552 | 71,175 | 1,369,069 |
| Kerala | 369,547 | 11,722,238 | 4,781 | 71,634 |
| Lakshadweep | 1,054 | 37,980 | 0 | 0 |
| Madhya Pradesh | 1,412,071 | 53,600,672 | 157,492 | 4,327,047 |
| Maharashtra | 1,800,173 | 66,976,397 | 191,644 | 4,343,847 |
| Manipur | 44,111 | 1,494,168 | 6,640 | 130,210 |
| Meghalaya | 63,039 | 2,146,909 | 10,219 | 183,745 |
| Mizoram | 17,546 | 647,962 | 5,163 | 110,309 |
| Nagaland | 28,111 | 789,692 | 10,708 | 179,707 |
| Delhi | 267,439 | 8,025,366 | 9,968 | 181,420 |
| Odisha | 687,131 | 21,013,160 | 21,196 | 383,985 |
| Puducherry | 16,049 | 792,114 | 1 | 12 |
| Punjab | 351,812 | 12,827,824 | 1,846 | 23,483 |
| Rajasthan | 1,281,401 | 45,125,445 | 177,042 | 4,240,087 |
| Sikkim | 5,151 | 187,760 | 11 | 321 |
| Tamil Nadu | 1,207,468 | 53,299,000 | 44,937 | 1,211,432 |
| Tripura | 48,282 | 1,219,218 | 9,419 | 147,604 |
| Uttar Pradesh | 3,831,505 | 143,958,033 | 680,012 | 16,446,426 |
| Uttarakhand | 169,422 | 6,536,554 | 15,725 | 424,824 |
| West Bengal | 1,568,740 | 51,772,435 | 32,937 | 537,300 |
| Telangana | 640,937 | 26,492,282 | 16,033 | 469,848 |
| India | 21,432,540 | 784,910,044 | 2,136,174 | 51,217,953 |

Table A6: Estimated cost (2020 US$) of 90% coverage of EPI (BCG, DPTCV, OPV, and MCV), RVV, and PCV vaccines among 12- to 23-month-old Indian children, by state

| State | Cost of 90% coverage, vaccine only | Cost of 90% coverage, vaccine and delivery | Incremental cost, current coverage to 90%, vaccine only | Incremental cost, current coverage to 90%, vaccine and delivery |
| --- | --- | --- | --- | --- |
| Andaman and Nicobar Islands | 81,053 | 196,401 | 76,553 | 115,694 |
| Andhra Pradesh | 12,291,389 | 52,428,438 | 11,575,341 | 25,199,296 |
| Arunachal Pradesh | 269,836 | 1,360,581 | 259,354 | 779,502 |
| Assam | 8,591,462 | 34,514,742 | 8,185,938 | 19,551,070 |
| Bihar | 43,073,758 | 163,885,585 | 40,733,314 | 80,487,901 |
| Chandigarh | 202,732 | 773,174 | 190,612 | 345,191 |
| Chhattisgarh | 8,128,776 | 33,187,216 | 7,645,134 | 15,832,925 |
| Dadra and Nagar Haveli | 82,086 | 422,357 | 77,565 | 201,853 |
| Daman and Diu | 38,057 | 193,999 | 36,064 | 94,440 |
| Goa | 329,467 | 1,490,775 | 309,571 | 698,212 |
| Gujarat | 14,246,188 | 60,031,887 | 13,514,873 | 31,241,907 |
| Haryana | 7,927,208 | 32,709,931 | 7,509,163 | 17,120,090 |
| Himachal Pradesh | 1,646,294 | 6,148,721 | 1,551,010 | 2,863,166 |
| Jammu and Kashmir | 3,132,057 | 11,726,111 | 2,950,339 | 5,741,904 |
| Jharkhand | 11,057,927 | 54,109,431 | 10,443,742 | 26,035,556 |
| Karnataka | 15,112,553 | 60,869,599 | 14,271,120 | 29,300,117 |
| Kerala | 6,119,595 | 22,508,144 | 5,754,829 | 10,857,540 |
| Lakshadweep | 17,448 | 68,874 | 16,394 | 30,894 |
| Madhya Pradesh | 23,383,515 | 100,861,795 | 22,128,936 | 51,588,168 |
| Maharashtra | 29,810,388 | 125,134,094 | 28,201,860 | 62,501,541 |
| Manipur | 730,464 | 2,841,790 | 692,993 | 1,477,831 |
| Meghalaya | 1,043,915 | 4,002,598 | 991,095 | 2,039,434 |
| Mizoram | 290,550 | 1,188,452 | 278,167 | 650,799 |
| Nagaland | 465,519 | 1,548,823 | 448,116 | 938,838 |
| Delhi | 4,428,724 | 15,422,940 | 4,171,253 | 7,578,994 |
| Odisha | 11,378,715 | 40,685,966 | 10,712,779 | 20,056,790 |
| Puducherry | 265,772 | 1,427,238 | 249,723 | 635,135 |
| Punjab | 5,825,919 | 23,092,418 | 5,475,953 | 10,288,076 |
| Rajasthan | 21,219,656 | 86,187,520 | 20,115,298 | 45,302,162 |
| Sikkim | 85,307 | 362,384 | 80,167 | 174,945 |
| Tamil Nadu | 19,995,352 | 96,868,292 | 18,832,821 | 44,780,723 |
| Tripura | 799,531 | 2,488,844 | 760,668 | 1,417,230 |
| Uttar Pradesh | 63,448,704 | 270,965,780 | 60,297,212 | 143,454,168 |
| Uttarakhand | 2,805,588 | 12,086,332 | 2,651,891 | 5,974,602 |
| West Bengal | 25,977,917 | 95,946,229 | 24,442,113 | 44,711,095 |
| Telangana | 10,613,739 | 48,960,063 | 9,988,836 | 22,937,627 |
| India | 354,917,161 | 1,466,697,524 | 335,620,797 | 733,005,416 |

Table A7: Estimated cost (2020 US$) of 90% coverage of BCG, HepB birth dose, pentavalent, OPV, IPV, MR, PCV, and RVV vaccines

| State | Cost of 90% coverage, vaccine only | Cost of 90% coverage, vaccine and delivery | Incremental cost, current coverage to 90%, vaccine only | Incremental cost, current coverage to 90%, vaccine and delivery |
| --- | --- | --- | --- | --- |
| Andaman and Nicobar Islands | 114,767 | 242,023 | 107,551 | 172,553 |
| Andhra Pradesh | 17,404,108 | 61,838,198 | 16,245,200 | 36,381,150 |
| Arunachal Pradesh | 382,077 | 1,590,258 | 364,876 | 1,069,925 |
| Assam | 12,165,162 | 40,928,860 | 11,505,204 | 27,632,747 |
| Bihar | 60,990,692 | 193,410,010 | 57,224,185 | 117,022,291 |
| Chandigarh | 287,061 | 908,241 | 267,479 | 507,334 |
| Chhattisgarh | 11,510,016 | 39,234,127 | 10,733,516 | 23,127,379 |
| Dadra and Nagar Haveli | 116,230 | 493,401 | 108,975 | 290,151 |
| Daman and Diu | 53,888 | 226,197 | 50,671 | 132,756 |
| Goa | 466,512 | 1,757,366 | 434,491 | 1,008,805 |
| Gujarat | 20,172,024 | 70,743,705 | 18,997,075 | 44,496,389 |
| Haryana | 11,224,604 | 38,636,069 | 10,551,297 | 24,342,117 |
| Himachal Pradesh | 2,331,086 | 7,245,295 | 2,178,104 | 4,218,563 |
| Jammu and Kashmir | 4,434,865 | 13,906,602 | 4,141,999 | 8,358,524 |
| Jharkhand | 15,657,576 | 63,392,618 | 14,676,617 | 37,652,411 |
| Karnataka | 21,398,762 | 71,732,843 | 20,038,760 | 42,225,845 |
| Kerala | 8,665,098 | 26,732,266 | 8,083,210 | 16,025,581 |
| Lakshadweep | 24,705 | 80,964 | 23,013 | 45,398 |
| Madhya Pradesh | 33,110,108 | 119,018,282 | 31,073,517 | 73,288,491 |
| Maharashtra | 42,210,300 | 147,583,167 | 39,617,550 | 89,500,390 |
| Manipur | 1,034,307 | 3,366,056 | 974,968 | 2,169,463 |
| Meghalaya | 1,478,142 | 4,728,429 | 1,394,089 | 2,977,786 |
| Mizoram | 411,407 | 1,398,471 | 391,131 | 913,922 |
| Nagaland | 659,156 | 1,849,701 | 630,642 | 1,313,825 |
| Delhi | 6,270,894 | 18,343,873 | 5,857,068 | 11,141,846 |
| Odisha | 16,111,798 | 48,412,792 | 15,040,636 | 29,423,399 |
| Puducherry | 376,322 | 1,666,255 | 350,509 | 910,462 |
| Punjab | 8,249,265 | 27,112,593 | 7,685,861 | 15,087,648 |
| Rajasthan | 30,046,171 | 102,055,308 | 28,257,584 | 64,507,982 |
| Sikkim | 120,792 | 429,358 | 112,549 | 254,317 |
| Tamil Nadu | 28,312,607 | 113,446,020 | 26,444,860 | 64,294,720 |
| Tripura | 1,132,103 | 2,994,208 | 1,069,703 | 2,068,996 |
| Uttar Pradesh | 89,840,791 | 319,821,382 | 84,810,380 | 204,725,213 |
| Uttarakhand | 3,972,599 | 14,224,547 | 3,725,569 | 8,630,432 |
| West Bengal | 36,783,676 | 113,340,194 | 34,357,390 | 66,831,573 |
| Telangana | 15,028,624 | 57,539,942 | 14,017,610 | 32,978,458 |
| India | 502,548,295 | 1,730,429,621 | 471,543,839 | 1,055,728,842 |

Table A8: Estimated cost (2020 US$) of closing vaccination gap from 2021 to 2024

|  | 2021 | | | 2022 | | | 2023 | | | 2024 | | |
| --- | --- | --- | --- | --- | --- | --- | --- | --- | --- | --- | --- | --- |
| State | EPI vaccines only | New schedule vaccines (BCG, OPV, IPV, pentavalent, MR, rotavirus, PCV) | EPI vaccines only | | New schedule vaccines (BCG, OPV, IPV, pentavalent, MR, rotavirus, PCV) | EPI vaccines only | | New schedule vaccines (BCG, OPV, IPV, pentavalent, MR, rotavirus, PCV) | EPI vaccines only | | New schedule vaccines (BCG, OPV, IPV, pentavalent, MR, rotavirus, PCV) |  |
| Andaman and Nicobar Islands | 960 | 62,911 | 1,934 | | 100,592 | 2,922 | | 138,812 | 3,924 | | 177,577 |  |
| Andhra Pradesh | 190,991 | 12,268,985 | 383,166 | | 20,448,393 | 576,531 | | 28,678,395 | 771,091 | | 36,959,226 |  |
| Arunachal Pradesh | 43,725 | 316,913 | 88,080 | | 574,592 | 133,072 | | 835,966 | 178,707 | | 1,101,074 |  |
| Assam | 744,763 | 8,805,557 | 1,500,994 | | 15,266,098 | 2,268,828 | | 21,825,610 | 3,048,397 | | 28,485,234 |  |
| Bihar | 1,321,826 | 40,201,251 | 2,679,077 | | 67,409,542 | 4,072,466 | | 95,339,799 | 5,502,716 | | 124,006,482 |  |
| Chandigarh | 626 | 179,925 | 1,260 | | 292,369 | 1,904 | | 406,423 | 2,557 | | 522,104 |  |
| Chhattisgarh | 50,083 | 7,942,770 | 100,927 | | 13,194,021 | 152,541 | | 18,524,633 | 204,934 | | 23,935,508 |  |
| Dadra and Nagar Haveli | 3,532 | 93,179 | 7,115 | | 160,681 | 10,749 | | 229,151 | 14,435 | | 298,598 |  |
| Daman and Diu | 2,412 | 41,840 | 4,858 | | 72,986 | 7,340 | | 104,578 | 9,857 | | 136,621 |  |
| Goa | 45,813 | 3,924,769 | 92,194 | | 6,407,454 | 139,149 | | 8,920,773 | 186,682 | | 11,465,011 |  |
| Gujarat | - | 338,814 | - | | 568,630 | - | | 801,738 | - | | 1,038,174 |  |
| Haryana | 884,312 | 14,441,229 | 1,781,181 | | 24,800,579 | 2,690,742 | | 35,306,303 | 3,613,128 | | 45,959,959 |  |
| Himachal Pradesh | 451,161 | 7,950,597 | 908,638 | | 13,596,583 | 1,372,497 | | 19,321,224 | 1,842,806 | | 25,125,343 |  |
| Jammu and Kashmir | 20,364 | 1,476,187 | 40,960 | | 2,412,988 | 61,791 | | 3,360,421 | 82,857 | | 4,318,575 |  |
| Jharkhand | 41,850 | 2,889,136 | 84,386 | | 4,775,625 | 127,616 | | 6,692,859 | 171,551 | | 8,641,214 |  |
| Karnataka | 404,235 | 12,273,432 | 816,311 | | 21,110,350 | 1,236,344 | | 30,117,552 | 1,664,449 | | 39,297,506 |  |
| Kerala | 344,834 | 14,237,591 | 692,151 | | 23,757,795 | 1,041,964 | | 33,346,359 | 1,394,287 | | 43,003,654 |  |
| Lakshadweep | 18,027 | 5,589,924 | 36,216 | | 9,144,699 | 54,568 | | 12,731,355 | 73,085 | | 16,350,104 |  |
| Madhya Pradesh | - | 15,922 | - | | 26,042 | - | | 36,308 | - | | 46,720 |  |
| Maharashtra | 1,096,474 | 24,055,933 | 2,210,710 | | 41,129,498 | 3,342,926 | | 58,478,077 | 4,493,338 | | 76,105,005 |  |
| Manipur | 1,093,889 | 29,394,480 | 2,196,967 | | 49,856,087 | 3,309,291 | | 70,489,053 | 4,430,921 | | 91,294,457 |  |
| Meghalaya | 32,787 | 717,550 | 66,046 | | 1,215,416 | 99,782 | | 1,720,415 | 134,000 | | 2,232,622 |  |
| Mizoram | 46,267 | 987,220 | 93,200 | | 1,669,825 | 140,807 | | 2,362,209 | 189,094 | | 3,064,477 |  |
| Nagaland | 27,776 | 286,907 | 55,951 | | 501,692 | 84,531 | | 719,555 | 113,520 | | 940,529 |  |
| Delhi | 45,250 | 409,264 | 91,152 | | 719,078 | 137,712 | | 1,033,332 | 184,938 | | 1,352,073 |  |
| Odisha | 96,783 | 10,290,553 | 194,786 | | 16,854,108 | 294,020 | | 23,499,956 | 394,496 | | 30,228,872 |  |
| Puducherry | 3 | 297,656 | 6 | | 507,740 | 9 | | 720,833 | 12 | | 936,968 |  |
| Punjab | 5,912 | 5,293,119 | 11,874 | | 8,629,763 | 17,886 | | 11,994,341 | 23,949 | | 15,387,029 |  |
| Rajasthan | 1,075,604 | 21,185,806 | 2,171,860 | | 36,287,670 | 3,289,065 | | 51,677,537 | 4,427,520 | | 67,359,545 |  |
| Sikkim | 81 | 85,986 | 163 | | 143,734 | 246 | | 202,309 | 330 | | 261,720 |  |
| Tamil Nadu | 304,554 | 21,113,579 | 610,631 | | 35,716,378 | 918,236 | | 50,392,060 | 1,227,376 | | 65,140,896 |  |
| Tripura | 118,402 | 11,021,146 | 238,059 | | 18,525,863 | 358,981 | | 26,109,819 | 481,178 | | 33,773,644 |  |
| Uttar Pradesh | 37,167 | 699,824 | 74,868 | | 1,169,541 | 113,111 | | 1,645,985 | 151,901 | | 2,129,230 |  |
| Uttarakhand | 4,172,458 | 65,715,294 | 8,422,524 | | 114,113,387 | 12,751,281 | | 163,406,002 | 17,159,824 | | 213,605,589 |  |
| West Bengal | 107,587 | 2,870,907 | 216,680 | | 4,861,944 | 327,295 | | 6,880,715 | 439,448 | | 8,927,511 |  |
| Telangana | 135,279 | 23,459,616 | 271,910 | | 38,265,478 | 409,905 | | 53,218,811 | 549,272 | | 68,320,724 |  |
| India | 12,965,787 | 350,935,772 | 26,146,835 | | 594,287,221 | 39,546,108 | | 841,269,268 | 53,166,580 | | 1,091,929,575 |  |

Table A9: Estimated cost (2020 US$) of 90% coverage of EPI (BCG, DPTCV, OPV, and MCV) among 12- to 23-month-old Indian children, by district

| State | District | Cost of 90% coverage, vaccine only | Cost of 90% coverage, vaccine and delivery | Incremental cost, current coverage to 90%, vaccine only | Incremental cost, current coverage to 90%, vaccine and delivery |
| --- | --- | --- | --- | --- | --- |
| Andaman and Nicobar Islands | Nicobars | 329 | 6,869 | 146 | 2,000 |
| Andaman and Nicobar Islands | North and Middle Andaman | 1,367 | 36,547 | 4 | 42 |
| Andaman and Nicobar Islands | South Andaman | 3,199 | 41,103 | 244 | 1,771 |
| Andhra Pradesh | Anantapur | 72,309 | 3,421,218 | 734 | 26,334 |
| Andhra Pradesh | Chittoor | 72,507 | 2,553,836 | 1,335 | 18,471 |
| Andhra Pradesh | East Godavari | 76,088 | 3,599,984 | 5,091 | 159,194 |
| Andhra Pradesh | Guntur | 69,455 | 3,286,166 | 2,544 | 92,453 |
| Andhra Pradesh | Krishna | 61,215 | 3,392,624 | 1,209 | 56,600 |
| Andhra Pradesh | Kurnool | 83,531 | 1,073,374 | 1,361 | 14,189 |
| Andhra Pradesh | Prakasam | 50,258 | 1,255,530 | 1,426 | 33,004 |
| Andhra Pradesh | Sri Potti Sriramulu Nellore | 36,687 | 1,735,812 | 6,550 | 194,260 |
| Andhra Pradesh | Srikakulam | 34,602 | 1,637,163 | 1,291 | 42,881 |
| Andhra Pradesh | Visakhapatnam | 45,959 | 2,174,500 | 1,130 | 40,544 |
| Andhra Pradesh | Vizianagaram | 36,881 | 1,744,990 | 1,279 | 46,101 |
| Andhra Pradesh | West Godavari | 46,064 | 928,328 | 1,037 | 15,311 |
| Andhra Pradesh | Y.S.R. | 56,688 | 1,184,645 | 1,210 | 19,690 |
| Arunachal Pradesh | Anjaw | 283 | 13,386 | 107 | 3,286 |
| Arunachal Pradesh | Changlang | 1,785 | 84,454 | 192 | 6,073 |
| Arunachal Pradesh | Dibang Valley | 56 | 2,634 | 10 | 303 |
| Arunachal Pradesh | East Kameng | 1,402 | 66,350 | 1,173 | 36,499 |
| Arunachal Pradesh | East Siang | 542 | 25,650 | 100 | 3,403 |
| Arunachal Pradesh | Kurung Kumey | 1,426 | 67,469 | 802 | 24,529 |
| Arunachal Pradesh | Lohit | 2,144 | 101,429 | 507 | 14,931 |
| Arunachal Pradesh | Lower Dibang Valley | 653 | 30,875 | 164 | 5,078 |
| Arunachal Pradesh | Lower Subansiri | 770 | 36,426 | 273 | 8,272 |
| Arunachal Pradesh | Papumpare | 2,755 | 130,340 | 690 | 20,194 |
| Arunachal Pradesh | Tawang | 419 | 19,819 | 195 | 6,154 |
| Arunachal Pradesh | Tirap | 1,038 | 35,374 | 328 | 5,602 |
| Arunachal Pradesh | Upper Siang | 94 | 1,968 | 27 | 326 |
| Arunachal Pradesh | Upper Subansiri | 1,046 | 49,489 | 538 | 16,543 |
| Arunachal Pradesh | West Kameng | 947 | 44,805 | 430 | 13,314 |
| Arunachal Pradesh | West Siang | 935 | 44,262 | 278 | 9,145 |
| Assam | Baksa | 15,876 | 400,128 | 1,309 | 17,351 |
| Assam | Barpeta | 27,632 | 1,307,390 | 9,630 | 310,252 |
| Assam | Bongaigaon | 10,351 | 258,584 | 1,549 | 33,287 |
| Assam | Cachar | 26,972 | 563,647 | 5,918 | 82,715 |
| Assam | Chirang | 8,844 | 418,420 | 2,170 | 68,561 |
| Assam | Darrang | 15,294 | 723,630 | 4,261 | 129,492 |
| Assam | Dhemaji | 11,743 | 555,623 | 1,208 | 37,141 |
| Assam | Dhubri | 44,408 | 2,101,106 | 25,765 | 790,445 |
| Assam | Dibrugarh | 17,405 | 465,270 | 489 | 7,990 |
| Assam | Dima Hasao | 3,346 | 158,300 | 281 | 9,098 |
| Assam | Goalpara | 17,689 | 369,650 | 3,401 | 49,132 |
| Assam | Golaghat | 16,087 | 336,185 | 736 | 12,179 |
| Assam | Hailakandi | 15,589 | 737,550 | 5,305 | 163,557 |
| Assam | Jorhat | 16,402 | 413,389 | 462 | 7,190 |
| Assam | Kamrup | 20,499 | 428,383 | 6,550 | 92,314 |
| Assam | Kamrup Metropolitan | 17,548 | 442,274 | 461 | 7,199 |
| Assam | Karbi Anglong | 12,460 | 589,536 | 4,891 | 151,654 |
| Assam | Karimganj | 30,366 | 634,565 | 6,433 | 86,326 |
| Assam | Kokrajhar | 13,918 | 771,367 | 2,726 | 107,501 |
| Assam | Lakhimpur | 16,580 | 784,442 | 1,904 | 58,161 |
| Assam | Morigaon | 17,890 | 846,447 | 2,482 | 81,960 |
| Assam | Nagaon | 58,414 | 1,459,286 | 14,616 | 298,455 |
| Assam | Nalbari | 12,021 | 568,778 | 1,436 | 42,768 |
| Assam | Sivasagar | 12,921 | 322,789 | 565 | 12,612 |
| Assam | Sonitpur | 26,753 | 1,482,695 | 6,340 | 263,200 |
| Assam | Tinsukia | 20,007 | 534,818 | 1,249 | 19,827 |
| Assam | Udalguri | 11,800 | 246,583 | 1,154 | 16,797 |
| Bihar | Araria | 80,967 | 1,692,002 | 11,839 | 158,025 |
| Bihar | Arwal | 17,196 | 346,546 | 196 | 3,080 |
| Bihar | Aurangabad | 41,624 | 1,418,345 | 614 | 9,796 |
| Bihar | Banka | 49,752 | 1,039,701 | 2,327 | 34,200 |
| Bihar | Begusarai | 80,126 | 2,303,127 | 1,180 | 21,066 |
| Bihar | Bhagalpur | 98,832 | 2,703,610 | 4,295 | 50,117 |
| Bihar | Bhojpur | 57,955 | 2,037,785 | 3,048 | 46,267 |
| Bihar | Buxar | 34,637 | 1,744,037 | 2,738 | 59,304 |
| Bihar | Darbhanga | 120,878 | 4,118,969 | 18,312 | 318,005 |
| Bihar | Gaya | 128,111 | 4,365,417 | 7,380 | 130,198 |
| Bihar | Gopalganj | 54,106 | 1,843,689 | 3,559 | 56,148 |
| Bihar | Jamui | 42,082 | 2,332,252 | 9,301 | 368,765 |
| Bihar | Jehanabad | 22,678 | 1,141,871 | 774 | 19,883 |
| Bihar | Kaimur (Bhabua) | 32,597 | 821,534 | 1,224 | 15,457 |
| Bihar | Katihar | 69,934 | 1,409,397 | 3,375 | 46,538 |
| Bihar | Khagaria | 52,472 | 1,788,000 | 2,206 | 36,030 |
| Bihar | Kishanganj | 43,987 | 1,175,851 | 6,779 | 86,755 |
| Bihar | Lakhisarai | 20,224 | 553,253 | 2,607 | 28,881 |
| Bihar | Madhepura | 52,733 | 1,854,192 | 3,477 | 51,576 |
| Bihar | Madhubani | 112,641 | 3,838,290 | 15,455 | 260,035 |
| Bihar | Munger | 30,422 | 613,099 | 1,445 | 17,196 |
| Bihar | Muzaffarpur | 103,210 | 5,196,799 | 11,483 | 282,761 |
| Bihar | Nalanda | 55,973 | 1,968,125 | 2,860 | 47,605 |
| Bihar | Nawada | 46,793 | 1,645,341 | 3,397 | 57,273 |
| Bihar | Pashchim Champaran | 94,946 | 4,492,220 | 26,612 | 769,586 |
| Bihar | Patna | 116,559 | 2,349,034 | 6,174 | 79,456 |
| Bihar | Purba Champaran | 153,678 | 4,203,971 | 38,881 | 460,111 |
| Bihar | Purnia | 93,673 | 3,293,696 | 2,530 | 47,698 |
| Bihar | Rohtas | 56,135 | 1,535,606 | 1,140 | 13,900 |
| Bihar | Saharsa | 52,404 | 1,400,862 | 1,905 | 29,064 |
| Bihar | Samastipur | 110,898 | 6,146,132 | 13,180 | 509,789 |
| Bihar | Saran | 94,501 | 5,404,716 | 12,987 | 448,665 |
| Bihar | Sheikhpura | 13,329 | 488,522 | 940 | 17,154 |
| Bihar | Sheohar | 21,002 | 738,461 | 2,085 | 33,908 |
| Bihar | Sitamarhi | 110,851 | 3,777,296 | 13,532 | 250,225 |
| Bihar | Siwan | 68,168 | 2,396,904 | 10,913 | 194,964 |
| Bihar | Supaul | 72,416 | 1,935,817 | 4,377 | 57,360 |
| Bihar | Vaishali | 92,624 | 2,476,014 | 5,542 | 75,953 |
| Chandigarh | Chandigarh | 12,242 | 430,468 | 122 | 2,485 |
| Chhattisgarh | Bastar | 30,508 | 637,533 | 414 | 6,741 |
| Chhattisgarh | Bijapur | 2,990 | 141,460 | 0 | 0 |
| Chhattisgarh | Bilaspur | 56,292 | 2,056,349 | 0 | 0 |
| Chhattisgarh | Dakshin Bastar Dantewada | 13,197 | 624,377 | 283 | 8,145 |
| Chhattisgarh | Dhamtari | 13,011 | 475,276 | 0 | 0 |
| Chhattisgarh | Durg | 52,682 | 1,316,082 | 0 | 0 |
| Chhattisgarh | Janjgir – Champa | 26,839 | 560,877 | 562 | 9,289 |
| Chhattisgarh | Jashpur | 15,724 | 743,945 | 1,084 | 34,723 |
| Chhattisgarh | Kabirdham | 17,827 | 372,545 | 803 | 13,361 |
| Chhattisgarh | Korba | 24,814 | 1,174,039 | 138 | 4,933 |
| Chhattisgarh | Korea (Koriya) | 13,302 | 629,354 | 314 | 9,110 |
| Chhattisgarh | Mahasamund | 23,272 | 819,676 | 209 | 2,852 |
| Chhattisgarh | Narayanpur | 2,077 | 98,267 | 119 | 3,649 |
| Chhattisgarh | Raigarh | 27,961 | 1,549,652 | 465 | 21,771 |
| Chhattisgarh | Raipur | 79,621 | 2,006,691 | 896 | 13,730 |
| Chhattisgarh | Rajnandgaon | 25,628 | 1,420,348 | 90 | 4,207 |
| Chhattisgarh | Surguja | 52,248 | 2,472,026 | 1,784 | 64,499 |
| Chhattisgarh | Uttar Bastar Kanker | 12,884 | 453,807 | 74 | 1,007 |
| Dadra and Nagar Haveli | Dadra and Nagar Haveli | 4,957 | 234,530 | 437 | 14,027 |
| Daman and Diu | Daman | 1,757 | 83,136 | 288 | 9,282 |
| Daman and Diu | Diu | 541 | 26,002 | 17 | 296 |
| Delhi | Central | 4,910 | 167,302 | 237 | 3,646 |
| Delhi | East | 20,282 | 542,170 | 847 | 9,635 |
| Delhi | New Delhi | 1,027 | 49,345 | 38 | 968 |
| Delhi | North | 11,998 | 664,928 | 847 | 37,673 |
| Delhi | North East | 42,427 | 1,069,296 | 3,812 | 55,096 |
| Delhi | North West | 44,421 | 570,814 | 712 | 7,058 |
| Delhi | South | 47,923 | 1,207,790 | 2,983 | 45,710 |
| Delhi | South West | 71,299 | 2,429,528 | 0 | 0 |
| Delhi | West | 23,153 | 1,324,193 | 494 | 21,633 |
| Goa | North Goa | 10,279 | 306,199 | 0 | 0 |
| Goa | South Goa | 9,617 | 486,364 | 0 | 0 |
| Gujarat | Ahmadabad | 98,932 | 2,644,649 | 10,416 | 129,796 |
| Gujarat | Amreli | 11,625 | 408,746 | 1,072 | 19,940 |
| Gujarat | Anand | 30,070 | 1,666,497 | 1,207 | 51,698 |
| Gujarat | Banaskantha | 55,458 | 3,073,577 | 16,554 | 674,776 |
| Gujarat | Bharuch | 25,406 | 893,312 | 1,642 | 22,245 |
| Gujarat | Bhavnagar | 26,356 | 898,100 | 5,025 | 83,145 |
| Gujarat | Dohad | 42,512 | 2,011,384 | 18,653 | 588,808 |
| Gujarat | Gandhinagar | 23,623 | 476,068 | 3,950 | 52,335 |
| Gujarat | Jamnagar | 32,387 | 1,103,598 | 495 | 8,963 |
| Gujarat | Junagadh | 24,750 | 517,223 | 2,016 | 26,666 |
| Gujarat | Kachchh | 27,476 | 967,758 | 4,277 | 53,233 |
| Gujarat | Kheda | 40,182 | 1,467,849 | 10,386 | 236,724 |
| Gujarat | Mahesana | 33,947 | 1,606,165 | 1,800 | 51,003 |
| Gujarat | Narmada | 10,563 | 266,218 | 295 | 2,937 |
| Gujarat | Navsari | 18,100 | 232,580 | 381 | 3,416 |
| Gujarat | Panchmahal | 33,588 | 1,861,512 | 14,009 | 573,613 |
| Gujarat | Patan | 17,651 | 978,233 | 5,987 | 241,759 |
| Gujarat | Porbandar | 5,338 | 181,909 | 161 | 2,904 |
| Gujarat | Rajkot | 48,898 | 1,021,842 | 2,230 | 37,474 |
| Gujarat | Sabarkantha | 40,202 | 1,902,083 | 2,982 | 81,022 |
| Gujarat | Surat | 89,033 | 4,212,469 | 11,420 | 333,939 |
| Gujarat | Surendranagar | 26,625 | 536,579 | 8,083 | 103,003 |
| Gujarat | Tapi | 10,204 | 482,808 | 162 | 6,176 |
| Gujarat | The Dangs | 4,164 | 196,998 | 478 | 14,438 |
| Gujarat | Vadodara | 60,100 | 2,047,944 | 3,601 | 75,923 |
| Gujarat | Valsad | 23,101 | 631,949 | 1,695 | 22,134 |
| Haryana | Ambala | 15,006 | 313,596 | 0 | 0 |
| Haryana | Bhiwani | 30,732 | 642,215 | 1,836 | 20,964 |
| Haryana | Faridabad | 45,230 | 2,139,979 | 10,361 | 302,592 |
| Haryana | Fatehabad | 19,195 | 654,072 | 27 | 542 |
| Haryana | Gurgaon | 35,940 | 1,700,454 | 7,464 | 239,358 |
| Haryana | Hisar | 24,222 | 610,471 | 561 | 4,689 |
| Haryana | Jhajjar | 15,004 | 709,901 | 1,455 | 36,856 |
| Haryana | Jind | 27,403 | 1,296,552 | 0 | 0 |
| Haryana | Kaithal | 19,265 | 911,500 | 0 | 0 |
| Haryana | Karnal | 32,118 | 809,458 | 0 | 0 |
| Haryana | Kurukshetra | 16,542 | 212,571 | 2 | 21 |
| Haryana | Mahendragarh | 13,433 | 635,548 | 84 | 3,010 |
| Haryana | Mewat | 41,583 | 1,967,456 | 25,059 | 770,190 |
| Haryana | Palwal | 23,554 | 1,114,406 | 9,772 | 302,311 |
| Haryana | Panchkula | 8,162 | 218,194 | 0 | 0 |
| Haryana | Panipat | 14,130 | 352,991 | 332 | 5,407 |
| Haryana | Rewari | 14,023 | 663,465 | 1,862 | 58,735 |
| Haryana | Rohtak | 28,149 | 588,241 | 953 | 13,999 |
| Haryana | Sirsa | 21,317 | 583,134 | 239 | 3,530 |
| Haryana | Sonipat | 20,701 | 979,430 | 651 | 23,159 |
| Haryana | Yamunanagar | 12,995 | 271,566 | 0 | 0 |
| Himachal Pradesh | Bilaspur | 5,198 | 182,782 | 433 | 8,888 |
| Himachal Pradesh | Chamba | 7,948 | 400,186 | 674 | 16,807 |
| Himachal Pradesh | Hamirpur | 7,943 | 198,438 | 1,017 | 20,109 |
| Himachal Pradesh | Kangra | 19,835 | 675,874 | 381 | 4,629 |
| Himachal Pradesh | Kinnaur | 780 | 16,300 | 14 | 224 |
| Himachal Pradesh | Kullu | 6,693 | 134,894 | 205 | 3,238 |
| Himachal Pradesh | Lahul and Spiti | 268 | 12,698 | 17 | 616 |
| Himachal Pradesh | Mandi | 16,254 | 444,648 | 251 | 3,688 |
| Himachal Pradesh | Shimla | 9,596 | 337,430 | 88 | 1,815 |
| Himachal Pradesh | Sirmaur | 6,415 | 175,493 | 139 | 2,031 |
| Himachal Pradesh | Solan | 8,781 | 299,211 | 357 | 7,223 |
| Himachal Pradesh | Una | 9,703 | 488,540 | 555 | 11,670 |
| Jammu and Kashmir | Anantnag | 15,394 | 562,335 | 101 | 2,700 |
| Jammu and Kashmir | Badgam | 9,806 | 334,149 | 18 | 359 |
| Jammu and Kashmir | Bandipore | 5,689 | 118,884 | 241 | 4,144 |
| Jammu and Kashmir | Baramula | 14,233 | 355,559 | 82 | 1,889 |
| Jammu and Kashmir | Doda | 6,531 | 163,163 | 1,623 | 31,987 |
| Jammu and Kashmir | Ganderbal | 5,039 | 184,086 | 1 | 31 |
| Jammu and Kashmir | Jammu | 18,586 | 534,222 | 599 | 8,344 |
| Jammu and Kashmir | Kargil | 1,660 | 78,539 | 3 | 92 |
| Jammu and Kashmir | Kathua | 9,316 | 327,579 | 0 | 0 |
| Jammu and Kashmir | Kishtwar | 4,766 | 127,400 | 187 | 2,186 |
| Jammu and Kashmir | Kulgam | 7,697 | 205,751 | 0 | 0 |
| Jammu and Kashmir | Kupwara | 11,483 | 306,961 | 59 | 684 |
| Jammu and Kashmir | Leh | 1,549 | 73,275 | 4 | 150 |
| Jammu and Kashmir | Pulwama | 7,124 | 143,569 | 0 | 0 |
| Jammu and Kashmir | Punch | 11,414 | 287,666 | 0 | 0 |
| Jammu and Kashmir | Rajouri | 14,037 | 664,157 | 2,444 | 65,945 |
| Jammu and Kashmir | Ramban | 5,995 | 149,775 | 826 | 14,838 |
| Jammu and Kashmir | Reasi | 6,610 | 312,759 | 263 | 4,927 |
| Jammu and Kashmir | Samba | 4,973 | 235,277 | 740 | 23,702 |
| Jammu and Kashmir | Shupiyan | 4,306 | 203,743 | 71 | 2,610 |
| Jammu and Kashmir | Srinagar | 14,371 | 489,682 | 0 | 0 |
| Jammu and Kashmir | Udhampur | 8,558 | 291,614 | 157 | 1,352 |
| Jharkhand | Bokaro | 28,395 | 1,343,462 | 1,534 | 49,896 |
| Jharkhand | Chatra | 30,888 | 1,461,419 | 6,925 | 211,421 |
| Jharkhand | Deoghar | 38,607 | 806,783 | 2,556 | 33,561 |
| Jharkhand | Dhanbad | 40,715 | 1,926,374 | 2,176 | 63,158 |
| Jharkhand | Dumka | 24,573 | 1,162,615 | 697 | 21,448 |
| Jharkhand | Garhwa | 38,328 | 1,813,453 | 5,625 | 176,221 |
| Jharkhand | Giridih | 58,228 | 2,754,963 | 6,438 | 211,716 |
| Jharkhand | Godda | 29,296 | 1,386,096 | 2,064 | 57,322 |
| Jharkhand | Gumla | 18,067 | 854,796 | 1,614 | 54,080 |
| Jharkhand | Hazaribagh | 39,483 | 825,099 | 1,060 | 17,238 |
| Jharkhand | Jamtara | 21,987 | 1,040,270 | 2,187 | 59,093 |
| Jharkhand | Khunti | 8,888 | 420,522 | 119 | 4,274 |
| Jharkhand | Kodarma | 16,942 | 938,926 | 594 | 21,356 |
| Jharkhand | Latehar | 13,171 | 623,145 | 2,243 | 69,725 |
| Jharkhand | Lohardaga | 8,090 | 382,785 | 504 | 11,943 |
| Jharkhand | Pakur | 21,122 | 999,373 | 696 | 25,091 |
| Jharkhand | Palamu | 48,140 | 2,277,678 | 5,609 | 171,879 |
| Jharkhand | Pashchimi Singhbhum | 25,419 | 1,202,658 | 4,154 | 120,304 |
| Jharkhand | Purbi Singhbhum | 33,208 | 1,571,211 | 411 | 14,704 |
| Jharkhand | Ramgarh | 14,689 | 695,002 | 508 | 18,384 |
| Jharkhand | Ranchi | 49,618 | 2,347,627 | 1,583 | 47,153 |
| Jharkhand | Sahibganj | 22,521 | 1,065,562 | 2,336 | 66,971 |
| Jharkhand | Saraikela Kharsawan | 26,563 | 1,256,783 | 1,119 | 40,121 |
| Jharkhand | Simdega | 10,822 | 512,043 | 821 | 27,713 |
| Karnataka | Bagalkot | 33,669 | 841,110 | 397 | 9,158 |
| Karnataka | Bangalore | 180,569 | 4,550,883 | 30,092 | 423,236 |
| Karnataka | Bangalore Rural | 13,122 | 620,869 | 690 | 18,477 |
| Karnataka | Belgaum | 75,730 | 1,908,612 | 2,508 | 30,167 |
| Karnataka | Bellary | 47,720 | 2,402,773 | 803 | 23,646 |
| Karnataka | Bidar | 22,894 | 478,429 | 1,277 | 16,427 |
| Karnataka | Bijapur | 29,493 | 743,319 | 4,191 | 51,330 |
| Karnataka | Chamarajanagar | 13,477 | 637,644 | 783 | 23,683 |
| Karnataka | Chikkaballapura | 13,635 | 645,122 | 951 | 21,935 |
| Karnataka | Chikmagalur | 12,014 | 568,428 | 902 | 29,452 |
| Karnataka | Chitradurga | 20,352 | 425,297 | 2,327 | 26,424 |
| Karnataka | Dakshina Kannada | 22,001 | 1,219,347 | 639 | 30,891 |
| Karnataka | Davanagere | 34,284 | 1,726,239 | 131 | 3,758 |
| Karnataka | Dharwad | 24,589 | 1,362,776 | 967 | 36,326 |
| Karnataka | Gadag | 14,326 | 288,718 | 1,469 | 15,833 |
| Karnataka | Gulbarga | 41,973 | 1,478,374 | 1,820 | 21,911 |
| Karnataka | Hassan | 22,576 | 1,068,132 | 531 | 14,777 |
| Karnataka | Haveri | 22,191 | 810,640 | 1,321 | 36,624 |
| Karnataka | Kodagu | 8,683 | 410,818 | 313 | 5,846 |
| Karnataka | Kolar | 21,586 | 451,101 | 240 | 3,902 |
| Karnataka | Koppal | 21,987 | 749,218 | 296 | 6,097 |
| Karnataka | Mandya | 29,031 | 1,373,579 | 1,489 | 50,562 |
| Karnataka | Mysore | 50,134 | 2,372,033 | 8,175 | 249,572 |
| Karnataka | Raichur | 23,445 | 824,378 | 1,332 | 26,083 |
| Karnataka | Ramanagara | 13,837 | 654,685 | 686 | 26,010 |
| Karnataka | Shimoga | 20,443 | 967,212 | 2,669 | 64,474 |
| Karnataka | Tumkur | 28,208 | 1,334,612 | 1,533 | 40,051 |
| Karnataka | Udupi | 10,975 | 385,911 | 373 | 7,869 |
| Karnataka | Uttara Kannada | 19,153 | 652,639 | 433 | 8,128 |
| Karnataka | Yadgir | 20,510 | 985,654 | 1,837 | 46,419 |
| Kerala | Alappuzha | 15,294 | 773,439 | 0 | 0 |
| Kerala | Ernakulam | 23,516 | 587,470 | 646 | 8,580 |
| Kerala | Idukki | 9,830 | 198,105 | 0 | 0 |
| Kerala | Kannur | 36,279 | 1,475,605 | 33 | 825 |
| Kerala | Kasaragod | 16,737 | 588,513 | 27 | 549 |
| Kerala | Kollam | 24,089 | 485,464 | 75 | 1,175 |
| Kerala | Kottayam | 26,222 | 961,047 | 0 | 0 |
| Kerala | Kozhikode | 37,139 | 1,356,681 | 1,216 | 21,254 |
| Kerala | Malappuram | 56,160 | 1,173,606 | 2,224 | 25,062 |
| Kerala | Palakkad | 41,282 | 1,451,562 | 0 | 0 |
| Kerala | Pathanamthitta | 13,244 | 757,468 | 0 | 0 |
| Kerala | Thiruvananthapuram | 33,251 | 427,278 | 263 | 3,012 |
| Kerala | Thrissur | 26,510 | 932,149 | 0 | 0 |
| Kerala | Wayanad | 9,993 | 553,850 | 298 | 11,177 |
| Lakshadweep | Lakshadweep | 1,054 | 37,980 | 0 | 0 |
| Madhya Pradesh | Alirajpur | 18,448 | 872,864 | 6,582 | 210,029 |
| Madhya Pradesh | Anuppur | 13,860 | 655,768 | 746 | 27,322 |
| Madhya Pradesh | Ashoknagar | 13,736 | 649,887 | 3,809 | 114,840 |
| Madhya Pradesh | Balaghat | 36,221 | 1,713,722 | 942 | 29,084 |
| Madhya Pradesh | Barwani | 39,474 | 1,867,674 | 11,055 | 339,829 |
| Madhya Pradesh | Betul | 30,531 | 1,444,542 | 819 | 29,381 |
| Madhya Pradesh | Bhind | 35,377 | 1,960,641 | 2,845 | 115,054 |
| Madhya Pradesh | Bhopal | 42,028 | 878,283 | 1,262 | 20,649 |
| Madhya Pradesh | Burhanpur | 14,908 | 705,340 | 2,562 | 75,839 |
| Madhya Pradesh | Chhatarpur | 32,899 | 1,556,560 | 6,570 | 200,618 |
| Madhya Pradesh | Chhindwara | 45,468 | 2,151,275 | 1,185 | 42,945 |
| Madhya Pradesh | Damoh | 24,254 | 1,147,528 | 2,144 | 63,868 |
| Madhya Pradesh | Datia | 13,882 | 769,376 | 1,472 | 59,873 |
| Madhya Pradesh | Dewas | 31,053 | 775,758 | 1,942 | 32,929 |
| Madhya Pradesh | Dhar | 44,350 | 926,804 | 1,331 | 16,599 |
| Madhya Pradesh | Dindori | 14,118 | 667,987 | 1,127 | 37,774 |
| Madhya Pradesh | Guna | 32,618 | 822,065 | 2,697 | 36,316 |
| Madhya Pradesh | Gwalior | 36,628 | 765,436 | 3,701 | 51,892 |
| Madhya Pradesh | Harda | 10,274 | 258,943 | 1,018 | 15,747 |
| Madhya Pradesh | Hoshangabad | 21,096 | 998,145 | 2,049 | 62,326 |
| Madhya Pradesh | Indore | 58,640 | 1,225,434 | 4,785 | 70,633 |
| Madhya Pradesh | Jabalpur | 41,227 | 861,533 | 1,073 | 17,137 |
| Madhya Pradesh | Jhabua | 25,671 | 1,214,606 | 6,916 | 228,247 |
| Madhya Pradesh | Katni | 23,982 | 1,134,662 | 1,332 | 43,256 |
| Madhya Pradesh | Khandwa (East Nimar) | 24,646 | 1,365,913 | 1,141 | 55,822 |
| Madhya Pradesh | Khargone (West Nimar) | 33,639 | 847,802 | 2,670 | 39,979 |
| Madhya Pradesh | Mandla | 17,059 | 807,121 | 659 | 24,364 |
| Madhya Pradesh | Mandsaur | 29,788 | 744,146 | 6,708 | 126,671 |
| Madhya Pradesh | Morena | 33,883 | 682,852 | 3,707 | 51,332 |
| Madhya Pradesh | Narsimhapur | 17,186 | 813,139 | 1,134 | 26,837 |
| Madhya Pradesh | Neemuch | 18,103 | 378,301 | 2,597 | 36,434 |
| Madhya Pradesh | Panna | 16,004 | 757,197 | 6,367 | 191,762 |
| Madhya Pradesh | Raisen | 27,481 | 967,956 | 197 | 2,684 |
| Madhya Pradesh | Rajgarh | 36,179 | 1,711,775 | 4,792 | 134,586 |
| Madhya Pradesh | Ratlam | 25,367 | 1,200,185 | 3,666 | 106,935 |
| Madhya Pradesh | Rewa | 45,354 | 2,145,874 | 3,249 | 98,885 |
| Madhya Pradesh | Sagar | 40,963 | 1,938,094 | 8,502 | 268,712 |
| Madhya Pradesh | Satna | 35,545 | 1,681,746 | 2,168 | 67,091 |
| Madhya Pradesh | Sehore | 20,999 | 438,823 | 932 | 13,763 |
| Madhya Pradesh | Seoni | 20,970 | 992,152 | 753 | 27,004 |
| Madhya Pradesh | Shahdol | 22,262 | 1,053,296 | 2,575 | 84,694 |
| Madhya Pradesh | Shajapur | 25,140 | 525,365 | 1,024 | 17,039 |
| Madhya Pradesh | Sheopur | 13,950 | 660,023 | 1,586 | 53,503 |
| Madhya Pradesh | Shivpuri | 40,784 | 852,278 | 3,523 | 48,161 |
| Madhya Pradesh | Sidhi | 27,859 | 1,318,088 | 7,521 | 232,311 |
| Madhya Pradesh | Singrauli | 31,108 | 1,471,852 | 5,667 | 181,166 |
| Madhya Pradesh | Tikamgarh | 25,451 | 1,204,179 | 6,033 | 191,477 |
| Madhya Pradesh | Ujjain | 37,769 | 943,544 | 2,583 | 60,276 |
| Madhya Pradesh | Umaria | 16,142 | 763,742 | 545 | 16,373 |
| Madhya Pradesh | Vidisha | 27,696 | 1,310,395 | 7,227 | 226,999 |
| Maharashtra | Ahmadnagar | 81,750 | 3,867,888 | 11,103 | 355,839 |
| Maharashtra | Akola | 27,805 | 1,315,546 | 2,727 | 92,547 |
| Maharashtra | Amravati | 41,588 | 1,967,684 | 3,163 | 110,924 |
| Maharashtra | Aurangabad | 50,344 | 2,381,971 | 2,388 | 74,742 |
| Maharashtra | Bhandara | 12,869 | 608,874 | 136 | 2,277 |
| Maharashtra | Bid | 49,635 | 1,037,258 | 4,944 | 68,330 |
| Maharashtra | Buldana | 36,719 | 767,345 | 3,680 | 49,532 |
| Maharashtra | Chandrapur | 25,977 | 1,229,063 | 586 | 21,020 |
| Maharashtra | Dhule | 34,644 | 1,920,006 | 3,434 | 153,083 |
| Maharashtra | Gadchiroli | 11,319 | 535,554 | 135 | 2,941 |
| Maharashtra | Gondiya | 19,665 | 930,410 | 508 | 14,434 |
| Maharashtra | Hingoli | 22,053 | 460,859 | 1,189 | 18,456 |
| Maharashtra | Jalgaon | 52,508 | 2,910,092 | 9,277 | 427,283 |
| Maharashtra | Jalna | 31,022 | 648,274 | 1,329 | 21,652 |
| Maharashtra | Kolhapur | 50,197 | 1,265,112 | 5,678 | 79,389 |
| Maharashtra | Latur | 44,420 | 2,461,848 | 2,024 | 88,070 |
| Maharashtra | Mumbai | 43,138 | 2,172,084 | 8,841 | 233,849 |
| Maharashtra | Mumbai Suburban | 117,666 | 2,458,924 | 27,790 | 394,062 |
| Maharashtra | Nagpur | 58,403 | 3,236,767 | 242 | 11,322 |
| Maharashtra | Nanded | 72,003 | 3,990,520 | 7,161 | 299,140 |
| Maharashtra | Nandurbar | 29,404 | 1,391,197 | 11,232 | 370,298 |
| Maharashtra | Nashik | 148,526 | 5,231,394 | 15,760 | 193,129 |
| Maharashtra | Osmanabad | 21,662 | 1,200,521 | 1,685 | 71,238 |
| Maharashtra | Parbhani | 32,985 | 1,828,075 | 1,915 | 90,953 |
| Maharashtra | Pune | 142,473 | 3,559,202 | 757 | 17,462 |
| Maharashtra | Raigarh | 50,755 | 2,401,387 | 2,537 | 91,556 |
| Maharashtra | Ratnagiri | 24,073 | 820,310 | 878 | 19,037 |
| Maharashtra | Sangli | 42,777 | 2,023,953 | 5,320 | 181,852 |
| Maharashtra | Satara | 53,160 | 1,872,404 | 4,404 | 54,712 |
| Maharashtra | Sindhudurg | 6,832 | 240,225 | 85 | 1,847 |
| Maharashtra | Solapur | 73,017 | 1,471,522 | 2,846 | 41,015 |
| Maharashtra | Thane | 212,024 | 5,343,645 | 45,744 | 635,206 |
| Maharashtra | Wardha | 13,300 | 737,132 | 302 | 9,378 |
| Maharashtra | Washim | 15,434 | 322,539 | 497 | 4,108 |
| Maharashtra | Yavatmal | 50,024 | 2,366,814 | 1,344 | 43,166 |
| Manipur | Bishnupur | 3,053 | 169,215 | 241 | 9,817 |
| Manipur | Chandel | 2,469 | 51,597 | 617 | 8,139 |
| Manipur | Churachandpur | 4,390 | 88,468 | 1,028 | 13,678 |
| Manipur | Imphal East | 8,090 | 216,265 | 711 | 9,823 |
| Manipur | Imphal West | 9,326 | 317,773 | 485 | 9,606 |
| Manipur | Senapati (excluding 3 subdivisions) | 3,722 | 47,826 | 723 | 6,296 |
| Manipur | Tamenglong | 2,485 | 31,932 | 707 | 6,057 |
| Manipur | Thoubal | 7,606 | 421,562 | 1,080 | 41,688 |
| Manipur | Ukhrul | 2,970 | 149,530 | 1,047 | 25,106 |
| Meghalaya | East Garo Hills | 8,460 | 176,792 | 3,395 | 46,689 |
| Meghalaya | East Khasi Hills | 16,885 | 580,454 | 1,851 | 29,942 |
| Meghalaya | Jaintia Hills | 10,553 | 314,374 | 1,243 | 23,574 |
| Meghalaya | Ribhoi | 5,810 | 279,241 | 1,028 | 26,184 |
| Meghalaya | South Garo Hills | 1,568 | 74,199 | 24 | 465 |
| Meghalaya | West Garo Hills | 9,876 | 246,730 | 1,696 | 30,071 |
| Meghalaya | West Khasi Hills | 9,886 | 475,121 | 982 | 26,819 |
| Mizoram | Aizawl | 6,188 | 297,395 | 1,496 | 35,707 |
| Mizoram | Champhai | 2,589 | 52,181 | 797 | 10,931 |
| Mizoram | Kolasib | 1,266 | 44,530 | 490 | 8,565 |
| Mizoram | Lawngtlai | 2,019 | 95,525 | 847 | 25,799 |
| Mizoram | Lunglei | 2,212 | 28,424 | 628 | 5,747 |
| Mizoram | Mamit | 1,415 | 66,970 | 534 | 16,560 |
| Mizoram | Saiha | 1,009 | 20,340 | 211 | 2,971 |
| Mizoram | Serchhip | 846 | 42,598 | 160 | 4,028 |
| Nagaland | Dimapur | 5,796 | 121,132 | 2,100 | 27,813 |
| Nagaland | Kiphire | 1,693 | 34,120 | 724 | 9,789 |
| Nagaland | Kohima | 2,468 | 65,986 | 351 | 4,403 |
| Nagaland | Longleng | 704 | 39,018 | 389 | 15,529 |
| Nagaland | Mokokchung | 1,681 | 48,308 | 247 | 3,891 |
| Nagaland | Mon | 5,203 | 177,295 | 3,277 | 57,258 |
| Nagaland | Peren | 1,355 | 34,148 | 448 | 5,708 |
| Nagaland | Phek | 2,686 | 77,204 | 758 | 10,893 |
| Nagaland | Tuensang | 3,540 | 94,630 | 1,093 | 15,135 |
| Nagaland | Wokha | 969 | 24,214 | 434 | 8,940 |
| Nagaland | Zunheboto | 2,016 | 73,636 | 886 | 20,348 |
| Odisha | Anugul | 25,309 | 528,885 | 0 | 0 |
| Odisha | Balangir | 30,321 | 1,680,457 | 0 | 0 |
| Odisha | Baleshwar | 28,547 | 713,148 | 313 | 3,128 |
| Odisha | Bargarh | 26,330 | 550,234 | 278 | 3,260 |
| Odisha | Baudh | 8,171 | 170,753 | 0 | 0 |
| Odisha | Bhadrak | 27,068 | 922,341 | 59 | 1,202 |
| Odisha | Cuttack | 31,284 | 855,791 | 533 | 7,759 |
| Odisha | Debagarh | 5,020 | 237,535 | 109 | 3,073 |
| Odisha | Dhenkanal | 19,831 | 938,252 | 59 | 2,101 |
| Odisha | Gajapati | 9,459 | 258,769 | 2,533 | 32,391 |
| Odisha | Ganjam | 52,232 | 1,428,844 | 4,064 | 45,423 |
| Odisha | Jagatsinghapur | 14,995 | 400,844 | 65 | 1,041 |
| Odisha | Jajapur | 26,485 | 340,336 | 0 | 0 |
| Odisha | Jharsuguda | 8,636 | 180,475 | 83 | 1,352 |
| Odisha | Kalahandi | 25,088 | 626,734 | 99 | 2,278 |
| Odisha | Kandhamal | 13,713 | 500,938 | 354 | 6,652 |
| Odisha | Kendrapara | 25,849 | 520,932 | 1,287 | 17,064 |
| Odisha | Kendujhar | 39,300 | 1,384,222 | 761 | 6,830 |
| Odisha | Khordha | 32,890 | 662,843 | 2,119 | 26,988 |
| Odisha | Koraput | 25,124 | 1,188,726 | 2,907 | 78,477 |
| Odisha | Malkangiri | 17,043 | 429,534 | 269 | 2,828 |
| Odisha | Mayurbhanj | 38,882 | 1,839,648 | 2,957 | 99,820 |
| Odisha | Nabarangapur | 26,577 | 1,257,452 | 656 | 20,522 |
| Odisha | Nayagarh | 14,230 | 501,222 | 0 | 0 |
| Odisha | Nuapada | 12,581 | 595,246 | 252 | 6,668 |
| Odisha | Puri | 24,670 | 317,010 | 0 | 0 |
| Odisha | Rayagada | 19,891 | 544,129 | 916 | 7,810 |
| Odisha | Sambalpur | 17,971 | 491,602 | 415 | 4,860 |
| Odisha | Subarnapur | 10,742 | 224,478 | 0 | 0 |
| Odisha | Sundargarh | 28,892 | 721,778 | 107 | 2,460 |
| Puducherry | Karaikal | 2,835 | 157,092 | 0 | 0 |
| Puducherry | Mahe | 377 | 21,543 | 0 | 0 |
| Puducherry | Puducherry | 12,090 | 572,040 | 0 | 0 |
| Puducherry | Yanam | 748 | 41,439 | 1 | 12 |
| Punjab | Amritsar | 33,257 | 955,933 | 0 | 0 |
| Punjab | Barnala | 5,961 | 301,468 | 0 | 0 |
| Punjab | Bathinda | 14,121 | 678,624 | 0 | 0 |
| Punjab | Faridkot | 10,056 | 289,037 | 0 | 0 |
| Punjab | Fatehgarh Sahib | 8,046 | 386,684 | 1 | 29 |
| Punjab | Firozpur | 30,165 | 806,370 | 72 | 498 |
| Punjab | Gurdaspur | 27,225 | 782,555 | 0 | 0 |
| Punjab | Hoshiarpur | 14,895 | 523,727 | 0 | 0 |
| Punjab | Jalandhar | 28,852 | 1,386,560 | 0 | 0 |
| Punjab | Kapurthala | 8,695 | 353,647 | 0 | 0 |
| Punjab | Ludhiana | 38,386 | 1,308,028 | 1,316 | 16,814 |
| Punjab | Mansa | 10,209 | 358,980 | 0 | 0 |
| Punjab | Moga | 10,987 | 528,009 | 0 | 0 |
| Punjab | Muktsar | 11,046 | 449,286 | 0 | 0 |
| Punjab | Patiala | 29,600 | 850,806 | 0 | 0 |
| Punjab | Rupnagar | 8,225 | 301,446 | 0 | 0 |
| Punjab | Sahibzada Ajit Singh Nagar | 15,498 | 780,340 | 0 | 0 |
| Punjab | Sangrur | 7,220 | 260,263 | 19 | 428 |
| Punjab | Shahid Bhagat Singh Nagar | 26,172 | 891,821 | 439 | 5,713 |
| Punjab | Tarn Taran | 13,197 | 634,241 | 0 | 0 |
| Rajasthan | Ajmer | 41,076 | 827,801 | 1,683 | 19,901 |
| Rajasthan | Alwar | 68,225 | 1,425,724 | 13,120 | 197,696 |
| Rajasthan | Banswara | 42,281 | 2,000,453 | 4,286 | 129,276 |
| Rajasthan | Baran | 23,892 | 602,137 | 647 | 10,284 |
| Rajasthan | Barmer | 53,364 | 2,524,819 | 20,990 | 631,721 |
| Rajasthan | Bharatpur | 65,277 | 1,744,973 | 16,237 | 233,873 |
| Rajasthan | Bhilwara | 46,556 | 1,163,038 | 1,367 | 27,871 |
| Rajasthan | Bikaner | 43,816 | 1,094,592 | 7,232 | 142,845 |
| Rajasthan | Bundi | 19,153 | 652,643 | 1,409 | 26,873 |
| Rajasthan | Chittaurgarh | 22,325 | 557,726 | 3,237 | 67,947 |
| Rajasthan | Churu | 33,273 | 1,844,020 | 2,556 | 106,324 |
| Rajasthan | Dausa | 27,807 | 581,099 | 2,341 | 34,430 |
| Rajasthan | Dhaulpur | 26,459 | 930,331 | 2,992 | 49,634 |
| Rajasthan | Dungarpur | 31,308 | 654,269 | 1,992 | 29,445 |
| Rajasthan | Ganganagar | 39,524 | 1,443,802 | 114 | 3,063 |
| Rajasthan | Hanumangarh | 33,307 | 1,575,879 | 2,000 | 72,566 |
| Rajasthan | Jaipur | 100,830 | 5,588,140 | 6,928 | 311,461 |
| Rajasthan | Jaisalmer | 15,626 | 866,042 | 4,653 | 181,172 |
| Rajasthan | Jalor | 43,879 | 884,292 | 16,074 | 216,997 |
| Rajasthan | Jhalawar | 19,361 | 390,191 | 263 | 4,227 |
| Rajasthan | Jhunjhunun | 36,471 | 762,144 | 1,171 | 18,267 |
| Rajasthan | Jodhpur | 66,277 | 3,673,142 | 11,414 | 469,731 |
| Rajasthan | Karauli | 29,160 | 797,701 | 3,863 | 50,046 |
| Rajasthan | Kota | 30,716 | 774,140 | 1,084 | 13,488 |
| Rajasthan | Nagaur | 53,775 | 2,544,308 | 10,058 | 319,886 |
| Rajasthan | Pali | 36,147 | 1,231,717 | 4,682 | 79,267 |
| Rajasthan | Pratapgarh | 18,426 | 871,787 | 1,033 | 29,846 |
| Rajasthan | Rajsamand | 21,180 | 272,158 | 1,704 | 15,499 |
| Rajasthan | Sawai Madhopur | 30,171 | 1,519,151 | 5,794 | 146,044 |
| Rajasthan | Sikar | 50,041 | 1,045,724 | 5,356 | 79,723 |
| Rajasthan | Sirohi | 26,294 | 529,898 | 6,392 | 84,086 |
| Rajasthan | Tonk | 23,811 | 837,255 | 237 | 4,839 |
| Rajasthan | Udaipur | 61,596 | 2,914,350 | 14,135 | 431,759 |
| Sikkim | East District | 2,507 | 62,617 | 5 | 127 |
| Sikkim | North District | 360 | 17,011 | 0 | 11 |
| Sikkim | South District | 1,318 | 62,341 | 0 | 0 |
| Sikkim | West District | 968 | 45,792 | 5 | 182 |
| Tamil Nadu | Ariyalur | 9,855 | 466,296 | 601 | 16,825 |
| Tamil Nadu | Chennai | 51,535 | 2,438,286 | 0 | 0 |
| Tamil Nadu | Coimbatore | 60,371 | 2,856,348 | 289 | 10,379 |
| Tamil Nadu | Cuddalore | 38,904 | 1,840,702 | 1,683 | 41,839 |
| Tamil Nadu | Dharmapuri | 25,506 | 1,206,775 | 1,471 | 41,650 |
| Tamil Nadu | Dindigul | 50,509 | 2,389,753 | 135 | 4,839 |
| Tamil Nadu | Erode | 49,139 | 1,730,788 | 54 | 323 |
| Tamil Nadu | Kancheepuram | 53,585 | 2,535,287 | 3,559 | 72,090 |
| Tamil Nadu | Kanniyakumari | 37,027 | 1,751,900 | 2,533 | 71,378 |
| Tamil Nadu | Karur | 23,451 | 490,067 | 0 | 0 |
| Tamil Nadu | Krishnagiri | 35,601 | 1,684,416 | 342 | 13,075 |
| Tamil Nadu | Madurai | 36,625 | 1,732,854 | 2,349 | 64,666 |
| Tamil Nadu | Nagapattinam | 26,030 | 1,231,551 | 6,475 | 186,753 |
| Tamil Nadu | Namakkal | 23,490 | 1,111,406 | 984 | 25,265 |
| Tamil Nadu | Perambalur | 10,229 | 483,962 | 443 | 12,884 |
| Tamil Nadu | Pudukkottai | 24,310 | 1,150,175 | 2,275 | 65,442 |
| Tamil Nadu | Ramanathapuram | 21,765 | 1,029,774 | 1,409 | 48,333 |
| Tamil Nadu | Salem | 79,425 | 3,757,893 | 1,546 | 51,249 |
| Tamil Nadu | Sivaganga | 17,282 | 957,785 | 537 | 26,049 |
| Tamil Nadu | Thanjavur | 41,035 | 857,534 | 193 | 3,145 |
| Tamil Nadu | The Nilgiris | 11,089 | 524,652 | 32 | 994 |
| Tamil Nadu | Theni | 20,274 | 959,246 | 1,411 | 40,419 |
| Tamil Nadu | Thiruvallur | 53,030 | 2,509,017 | 537 | 16,213 |
| Tamil Nadu | Thiruvarur | 26,487 | 1,253,186 | 494 | 18,862 |
| Tamil Nadu | Thoothukkudi | 19,145 | 400,092 | 2,680 | 41,283 |
| Tamil Nadu | Tiruchirappalli | 45,407 | 2,148,358 | 1,250 | 46,164 |
| Tamil Nadu | Tirunelveli | 44,192 | 923,494 | 2,096 | 36,123 |
| Tamil Nadu | Tiruppur | 50,512 | 2,389,900 | 0 | 0 |
| Tamil Nadu | Tiruvannamalai | 40,866 | 1,933,505 | 1,955 | 45,813 |
| Tamil Nadu | Vellore | 86,155 | 4,076,283 | 1,906 | 42,834 |
| Tamil Nadu | Viluppuram | 62,121 | 2,939,153 | 1,847 | 36,422 |
| Tamil Nadu | Virudhunagar | 32,518 | 1,538,563 | 3,851 | 130,120 |
| Telangana | Adilabad | 37,915 | 487,204 | 1,354 | 10,717 |
| Telangana | Hyderabad | 121,695 | 5,757,832 | 2,425 | 75,024 |
| Telangana | Karimnagar | 55,214 | 2,612,354 | 3 | 114 |
| Telangana | Khammam | 44,907 | 938,454 | 998 | 16,500 |
| Telangana | Mahbubnagar | 61,660 | 2,917,345 | 4,119 | 139,617 |
| Telangana | Medak | 43,317 | 2,049,463 | 0 | 0 |
| Telangana | Nalgonda | 50,603 | 2,394,185 | 1,661 | 52,526 |
| Telangana | Nizamabad | 50,718 | 1,059,872 | 1,146 | 18,647 |
| Telangana | Rangareddy | 118,054 | 5,585,542 | 2,083 | 74,735 |
| Telangana | Warangal | 56,855 | 2,690,031 | 2,243 | 81,968 |
| Tripura | Dhalai | 4,660 | 220,500 | 1,308 | 40,276 |
| Tripura | North Tripura | 11,052 | 278,552 | 2,779 | 36,360 |
| Tripura | South Tripura | 9,188 | 231,555 | 2,051 | 26,785 |
| Tripura | West Tripura | 23,381 | 488,610 | 3,280 | 44,183 |
| Uttar Pradesh | Agra | 87,515 | 2,205,637 | 8,878 | 125,235 |
| Uttar Pradesh | Aligarh | 80,063 | 2,190,177 | 6,032 | 80,317 |
| Uttar Pradesh | Allahabad | 116,162 | 6,437,878 | 23,208 | 898,761 |
| Uttar Pradesh | Ambedkar Nagar | 48,788 | 2,308,330 | 5,778 | 181,707 |
| Uttar Pradesh | Auraiya | 25,345 | 1,199,142 | 7,968 | 252,936 |
| Uttar Pradesh | Azamgarh | 64,032 | 3,548,773 | 15,602 | 589,189 |
| Uttar Pradesh | Baghpat | 26,531 | 709,220 | 1,562 | 18,104 |
| Uttar Pradesh | Bahraich | 90,020 | 4,259,177 | 66,961 | 2,085,914 |
| Uttar Pradesh | Ballia | 60,448 | 1,263,215 | 13,158 | 170,852 |
| Uttar Pradesh | Balrampur | 48,822 | 1,020,269 | 31,209 | 438,667 |
| Uttar Pradesh | Banda | 21,830 | 1,032,850 | 3,591 | 112,873 |
| Uttar Pradesh | Bara Banki | 53,418 | 2,527,404 | 11,813 | 346,748 |
| Uttar Pradesh | Bareilly | 82,116 | 2,069,574 | 16,337 | 204,729 |
| Uttar Pradesh | Basti | 46,837 | 943,916 | 4,846 | 67,282 |
| Uttar Pradesh | Bijnor | 67,379 | 3,734,215 | 2,649 | 118,448 |
| Uttar Pradesh | Budaun | 87,461 | 4,847,205 | 15,379 | 590,037 |
| Uttar Pradesh | Bulandshahr | 68,801 | 2,513,305 | 8,704 | 199,482 |
| Uttar Pradesh | Chandauli | 42,333 | 2,002,924 | 5,016 | 157,815 |
| Uttar Pradesh | Chitrakoot | 19,705 | 932,293 | 1,026 | 29,553 |
| Uttar Pradesh | Deoria | 54,664 | 1,461,280 | 4,992 | 62,383 |
| Uttar Pradesh | Etah | 36,911 | 1,300,083 | 7,635 | 85,169 |
| Uttar Pradesh | Etawah | 27,710 | 1,311,040 | 4,990 | 149,576 |
| Uttar Pradesh | Faizabad | 37,465 | 1,772,626 | 6,269 | 172,864 |
| Uttar Pradesh | Farrukhabad | 36,886 | 770,817 | 9,482 | 126,878 |
| Uttar Pradesh | Fatehpur | 41,364 | 1,957,059 | 7,235 | 223,167 |
| Uttar Pradesh | Firozabad | 49,827 | 1,041,260 | 5,605 | 74,327 |
| Uttar Pradesh | Gautam Buddha Nagar | 43,104 | 2,039,419 | 4,670 | 133,990 |
| Uttar Pradesh | Ghaziabad | 88,638 | 4,193,788 | 12,047 | 374,003 |
| Uttar Pradesh | Ghazipur | 59,567 | 1,244,808 | 10,243 | 145,642 |
| Uttar Pradesh | Gonda | 63,100 | 1,576,352 | 23,244 | 453,924 |
| Uttar Pradesh | Gorakhpur | 82,450 | 3,011,890 | 3,803 | 93,881 |
| Uttar Pradesh | Hamirpur | 12,041 | 569,725 | 2,618 | 83,356 |
| Uttar Pradesh | Hardoi | 69,572 | 3,291,686 | 16,600 | 492,598 |
| Uttar Pradesh | Jalaun | 21,763 | 1,029,683 | 4,299 | 132,489 |
| Uttar Pradesh | Jaunpur | 81,883 | 2,045,579 | 14,756 | 289,739 |
| Uttar Pradesh | Jhansi | 28,077 | 586,735 | 1,780 | 23,097 |
| Uttar Pradesh | Jyotiba Phule Nagar | 37,642 | 2,086,180 | 3,057 | 126,979 |
| Uttar Pradesh | Kannauj | 31,442 | 657,052 | 6,492 | 90,139 |
| Uttar Pradesh | Kanpur Dehat | 31,973 | 1,512,772 | 3,444 | 99,122 |
| Uttar Pradesh | Kanpur Nagar | 75,883 | 3,590,293 | 9,433 | 263,110 |
| Uttar Pradesh | Kanshiram Nagar | 33,300 | 1,845,560 | 7,332 | 272,026 |
| Uttar Pradesh | Kaushambi | 28,015 | 1,325,468 | 6,204 | 185,342 |
| Uttar Pradesh | Kheri | 101,285 | 4,792,166 | 12,113 | 355,779 |
| Uttar Pradesh | Kushinagar | 75,652 | 2,763,574 | 18,246 | 405,482 |
| Uttar Pradesh | Lalitpur | 22,882 | 1,082,606 | 1,352 | 41,545 |
| Uttar Pradesh | Lucknow | 49,077 | 630,642 | 5,402 | 48,351 |
| Uttar Pradesh | Mahamaya Nagar | 32,489 | 811,640 | 5,064 | 94,065 |
| Uttar Pradesh | Mahoba | 12,305 | 257,143 | 1,040 | 13,935 |
| Uttar Pradesh | Mahrajganj | 48,211 | 2,671,898 | 9,174 | 357,822 |
| Uttar Pradesh | Mainpuri | 31,173 | 1,727,664 | 3,892 | 158,463 |
| Uttar Pradesh | Mathura | 59,596 | 1,201,053 | 9,329 | 127,280 |
| Uttar Pradesh | Mau | 43,122 | 901,133 | 7,666 | 109,030 |
| Uttar Pradesh | Meerut | 85,508 | 2,136,134 | 12,103 | 222,987 |
| Uttar Pradesh | Mirzapur | 52,690 | 2,492,958 | 6,765 | 205,191 |
| Uttar Pradesh | Moradabad | 98,326 | 3,463,240 | 20,198 | 244,004 |
| Uttar Pradesh | Muzaffarnagar | 105,430 | 2,633,824 | 13,290 | 243,953 |
| Uttar Pradesh | Pilibhit | 36,484 | 919,494 | 2,299 | 28,856 |
| Uttar Pradesh | Pratapgarh | 45,334 | 913,629 | 5,337 | 74,035 |
| Uttar Pradesh | Rae Bareli | 52,960 | 2,505,734 | 3,617 | 99,536 |
| Uttar Pradesh | Rampur | 52,639 | 1,100,027 | 3,342 | 40,322 |
| Uttar Pradesh | Saharanpur | 73,671 | 4,082,949 | 5,360 | 230,127 |
| Uttar Pradesh | Sant Kabir Nagar | 37,906 | 1,908,625 | 5,811 | 135,337 |
| Uttar Pradesh | Sant Ravidas Nagar (Bhadohi) | 38,854 | 970,628 | 8,717 | 171,279 |
| Uttar Pradesh | Shahjahanpur | 58,754 | 3,256,206 | 5,013 | 179,560 |
| Uttar Pradesh | Shrawasti | 32,076 | 1,517,619 | 18,073 | 558,419 |
| Uttar Pradesh | Siddharth Nagar | 50,063 | 1,760,287 | 16,988 | 298,185 |
| Uttar Pradesh | Sitapur | 103,062 | 4,876,232 | 16,033 | 498,726 |
| Uttar Pradesh | Sonbhadra | 42,753 | 2,022,790 | 11,629 | 350,578 |
| Uttar Pradesh | Sultanpur | 60,388 | 2,126,988 | 12,431 | 153,866 |
| Uttar Pradesh | Unnao | 54,696 | 1,143,004 | 7,790 | 99,028 |
| Uttar Pradesh | Varanasi | 63,237 | 1,321,490 | 5,991 | 78,232 |
| Uttarakhand | Almora | 7,728 | 263,326 | 392 | 6,670 |
| Uttarakhand | Bageshwar | 4,075 | 82,130 | 417 | 5,723 |
| Uttarakhand | Chamoli | 6,473 | 177,079 | 594 | 7,608 |
| Uttarakhand | Champawat | 3,874 | 195,076 | 137 | 3,376 |
| Uttarakhand | Dehradun | 28,340 | 714,264 | 1,071 | 16,212 |
| Uttarakhand | Garhwal | 9,454 | 238,281 | 439 | 6,114 |
| Uttarakhand | Hardwar | 42,701 | 2,020,316 | 7,073 | 211,350 |
| Uttarakhand | Nainital | 14,324 | 793,843 | 1,131 | 43,179 |
| Uttarakhand | Pithoragarh | 6,439 | 226,423 | 120 | 2,453 |
| Uttarakhand | Rudraprayag | 4,365 | 91,211 | 221 | 3,226 |
| Uttarakhand | Tehri Garhwal | 9,127 | 333,423 | 795 | 18,386 |
| Uttarakhand | Udham Singh Nagar | 27,316 | 1,292,408 | 3,107 | 96,828 |
| Uttarakhand | Uttarkashi | 5,205 | 108,774 | 227 | 3,699 |
| West Bengal | Bankura | 53,115 | 1,867,630 | 0 | 0 |
| West Bengal | Barddhaman | 121,195 | 4,441,854 | 2,949 | 65,762 |
| West Bengal | Birbhum | 63,840 | 2,175,356 | 0 | 0 |
| West Bengal | Dakshin Dinajpur | 25,464 | 895,372 | 744 | 12,863 |
| West Bengal | Darjiling | 24,466 | 833,700 | 5 | 44 |
| West Bengal | Haora | 73,754 | 1,971,589 | 2,643 | 31,730 |
| West Bengal | Hugli | 88,171 | 4,237,312 | 391 | 11,533 |
| West Bengal | Jalpaiguri | 61,189 | 2,151,515 | 151 | 3,091 |
| West Bengal | Koch Bihar | 48,846 | 1,231,065 | 905 | 13,876 |
| West Bengal | Kolkata | 43,674 | 1,167,492 | 1,526 | 19,173 |
| West Bengal | Maldah | 89,214 | 2,248,468 | 5,320 | 74,479 |
| West Bengal | Murshidabad | 187,449 | 4,682,795 | 2,806 | 64,686 |
| West Bengal | Nadia | 95,241 | 3,245,360 | 0 | 0 |
| West Bengal | North Twenty Four Parganas | 108,959 | 3,530,887 | 469 | 9,577 |
| West Bengal | Paschim Medinipur | 104,803 | 3,685,066 | 0 | 0 |
| West Bengal | Purba Medinipur | 73,985 | 2,601,449 | 2,047 | 41,792 |
| West Bengal | Puruliya | 63,267 | 3,185,614 | 627 | 17,995 |
| West Bengal | South Twenty Four Parganas | 171,106 | 5,830,486 | 2,491 | 50,382 |
| West Bengal | Uttar Dinajpur | 71,001 | 1,789,428 | 9,861 | 120,319 |
| India |  | 21,432,537 | 784,910,044 | 2,136,163 | 51,217,951 |

Table A10: Estimated cost (2020 US$) of 90% coverage of EPI (BCG, DPTCV, OPV, and MCV), RVV, and PCV vaccines among 12- to 23-month-old Indian children, by district

| State | District | Cost of 90% coverage, vaccine only | Cost of 90% coverage, vaccine and delivery | Incremental cost, current coverage to 90%, vaccine only | Incremental cost, current coverage to 90%, vaccine and delivery |
| --- | --- | --- | --- | --- | --- |
| Andaman and Nicobar Islands | Nicobars | 5,443 | 15,197 | 5,260 | 10,329 |
| Andaman and Nicobar Islands | North & Middle Andaman | 22,640 | 71,023 | 21,277 | 34,518 |
| Andaman and Nicobar Islands | South Andaman | 52,970 | 110,180 | 50,015 | 70,848 |
| Andhra Pradesh | Anantapur | 1,197,426 | 6,161,143 | 1,125,850 | 2,766,258 |
| Andhra Pradesh | Chittoor | 1,200,689 | 4,268,435 | 1,129,517 | 1,733,070 |
| Andhra Pradesh | East Godavari | 1,259,994 | 6,483,076 | 1,188,997 | 3,042,286 |
| Andhra Pradesh | Guntur | 1,150,157 | 5,917,932 | 1,083,246 | 2,724,218 |
| Andhra Pradesh | Krishna | 1,013,704 | 6,140,749 | 953,698 | 2,804,724 |
| Andhra Pradesh | Kurnool | 1,383,253 | 2,877,261 | 1,301,083 | 1,818,076 |
| Andhra Pradesh | Prakasam | 832,260 | 2,747,348 | 783,428 | 1,524,822 |
| Andhra Pradesh | Sri Potti Sriramulu Nellore | 607,534 | 3,125,959 | 577,397 | 1,584,406 |
| Andhra Pradesh | Srikakulam | 573,007 | 2,948,305 | 539,695 | 1,354,023 |
| Andhra Pradesh | Visakhapatnam | 761,074 | 3,915,974 | 716,245 | 1,782,018 |
| Andhra Pradesh | Vizianagaram | 610,746 | 3,142,486 | 575,143 | 1,443,597 |
| Andhra Pradesh | West Godavari | 762,803 | 2,078,786 | 717,776 | 1,165,769 |
| Andhra Pradesh | Y.S.R. | 938,743 | 2,620,984 | 883,265 | 1,456,028 |
| Arunachal Pradesh | Anjaw | 4,685 | 24,107 | 4,509 | 14,007 |
| Arunachal Pradesh | Changlang | 29,559 | 152,090 | 27,966 | 73,709 |
| Arunachal Pradesh | Dibang Valley | 922 | 4,743 | 877 | 2,412 |
| Arunachal Pradesh | East Kameng | 23,222 | 119,487 | 22,993 | 89,636 |
| Arunachal Pradesh | East Siang | 8,977 | 46,192 | 8,535 | 23,945 |
| Arunachal Pradesh | Kurung Kumey | 23,614 | 121,502 | 22,990 | 78,562 |
| Arunachal Pradesh | Lohit | 35,500 | 182,660 | 33,864 | 96,162 |
| Arunachal Pradesh | Lower Dibang Valley | 10,806 | 55,602 | 10,318 | 29,805 |
| Arunachal Pradesh | Lower Subansiri | 12,749 | 65,598 | 12,252 | 37,444 |
| Arunachal Pradesh | Papumpare | 45,619 | 234,725 | 43,554 | 124,579 |
| Arunachal Pradesh | Tawang | 6,937 | 35,692 | 6,713 | 22,027 |
| Arunachal Pradesh | Tirap | 17,191 | 64,308 | 16,481 | 34,536 |
| Arunachal Pradesh | Upper Siang | 1,560 | 4,355 | 1,492 | 2,712 |
| Arunachal Pradesh | Upper Subansiri | 17,321 | 89,123 | 16,813 | 56,177 |
| Arunachal Pradesh | West Kameng | 15,682 | 80,688 | 15,165 | 49,197 |
| Arunachal Pradesh | West Siang | 15,492 | 79,709 | 14,834 | 44,592 |
| Assam | Baksa | 262,906 | 792,810 | 248,339 | 410,033 |
| Assam | Barpeta | 457,586 | 2,354,429 | 439,584 | 1,357,292 |
| Assam | Bongaigaon | 171,409 | 565,834 | 162,607 | 340,537 |
| Assam | Cachar | 446,648 | 1,247,048 | 425,595 | 766,117 |
| Assam | Chirang | 146,447 | 753,517 | 139,774 | 403,658 |
| Assam | Darrang | 253,270 | 1,303,159 | 242,237 | 709,021 |
| Assam | Dhemaji | 194,468 | 1,000,601 | 183,933 | 482,119 |
| Assam | Dhubri | 735,386 | 3,783,803 | 716,743 | 2,473,141 |
| Assam | Dibrugarh | 288,222 | 904,168 | 271,307 | 446,888 |
| Assam | Dima Hasao | 55,405 | 285,077 | 52,340 | 135,875 |
| Assam | Goalpara | 292,920 | 817,837 | 278,633 | 497,319 |
| Assam | Golaghat | 266,402 | 743,798 | 251,051 | 419,792 |
| Assam | Hailakandi | 258,142 | 1,328,226 | 247,858 | 754,234 |
| Assam | Jorhat | 271,620 | 819,085 | 255,679 | 412,886 |
| Assam | Kamrup | 339,462 | 947,782 | 325,512 | 611,713 |
| Assam | Kamrup Metropolitan | 290,598 | 876,317 | 273,511 | 441,242 |
| Assam | Karbi Anglong | 206,338 | 1,061,674 | 198,768 | 623,792 |
| Assam | Karimganj | 502,845 | 1,403,951 | 478,912 | 855,712 |
| Assam | Kokrajhar | 230,482 | 1,396,196 | 219,290 | 732,330 |
| Assam | Lakhimpur | 274,554 | 1,412,672 | 259,879 | 686,390 |
| Assam | Morigaon | 296,256 | 1,524,334 | 280,848 | 759,847 |
| Assam | Nagaon | 967,325 | 3,193,206 | 923,527 | 2,032,376 |
| Assam | Nalbari | 199,072 | 1,024,292 | 188,487 | 498,281 |
| Assam | Sivasagar | 213,969 | 706,327 | 201,614 | 396,150 |
| Assam | Sonitpur | 443,024 | 2,683,722 | 422,611 | 1,464,226 |
| Assam | Tinsukia | 331,305 | 1,039,322 | 312,547 | 524,331 |
| Assam | Udalguri | 195,398 | 545,555 | 184,753 | 315,769 |
| Bihar | Araria | 1,340,785 | 3,743,492 | 1,271,657 | 2,209,514 |
| Bihar | Arwal | 284,755 | 776,014 | 267,756 | 432,548 |
| Bihar | Aurangabad | 689,278 | 2,578,502 | 648,269 | 1,169,953 |
| Bihar | Banka | 823,885 | 2,300,300 | 776,460 | 1,294,800 |
| Bihar | Begusarai | 1,326,863 | 4,410,693 | 1,247,917 | 2,128,631 |
| Bihar | Bhagalpur | 1,636,628 | 5,100,387 | 1,542,092 | 2,446,894 |
| Bihar | Bhojpur | 959,712 | 3,660,117 | 904,806 | 1,668,599 |
| Bihar | Buxar | 573,583 | 2,897,614 | 541,685 | 1,212,881 |
| Bihar | Darbhanga | 2,001,711 | 7,488,142 | 1,899,144 | 3,687,178 |
| Bihar | Gaya | 2,121,478 | 7,936,176 | 2,000,747 | 3,700,957 |
| Bihar | Gopalganj | 895,985 | 3,351,763 | 845,437 | 1,564,222 |
| Bihar | Jamui | 696,869 | 4,221,444 | 664,088 | 2,257,957 |
| Bihar | Jehanabad | 375,541 | 1,897,151 | 353,637 | 775,164 |
| Bihar | Kaimur (Bhabua) | 539,793 | 1,627,779 | 508,421 | 821,702 |
| Bihar | Katihar | 1,158,095 | 3,156,033 | 1,091,536 | 1,793,174 |
| Bihar | Khagaria | 868,921 | 3,250,521 | 818,655 | 1,498,551 |
| Bihar | Kishanganj | 728,407 | 2,285,052 | 691,200 | 1,195,957 |
| Bihar | Lakhisarai | 334,911 | 1,043,717 | 317,293 | 519,345 |
| Bihar | Madhepura | 873,247 | 3,330,361 | 823,991 | 1,527,745 |
| Bihar | Madhubani | 1,865,308 | 6,977,876 | 1,768,121 | 3,399,622 |
| Bihar | Munger | 503,780 | 1,372,899 | 474,803 | 776,996 |
| Bihar | Muzaffarpur | 1,709,137 | 8,634,174 | 1,617,410 | 3,720,136 |
| Bihar | Nalanda | 926,905 | 3,534,999 | 873,792 | 1,614,479 |
| Bihar | Nawada | 774,887 | 2,955,239 | 731,491 | 1,367,170 |
| Bihar | Pashchim Champaran | 1,572,276 | 8,089,870 | 1,503,942 | 4,367,235 |
| Bihar | Patna | 1,930,190 | 5,260,142 | 1,819,805 | 2,990,564 |
| Bihar | Purba Champaran | 2,544,870 | 7,930,832 | 2,430,073 | 4,186,972 |
| Bihar | Purnia | 1,551,194 | 5,915,889 | 1,460,051 | 2,669,891 |
| Bihar | Rohtas | 929,578 | 2,896,936 | 874,583 | 1,375,230 |
| Bihar | Saharsa | 867,796 | 2,722,321 | 817,297 | 1,350,522 |
| Bihar | Samastipur | 1,836,442 | 11,124,678 | 1,738,725 | 5,488,336 |
| Bihar | Saran | 1,564,918 | 9,464,646 | 1,483,404 | 4,508,595 |
| Bihar | Sheikhpura | 220,728 | 877,738 | 208,338 | 406,370 |
| Bihar | Sheohar | 347,784 | 1,326,368 | 328,867 | 621,815 |
| Bihar | Sitamarhi | 1,835,666 | 6,866,992 | 1,738,347 | 3,339,921 |
| Bihar | Siwan | 1,128,842 | 4,305,139 | 1,071,587 | 2,103,199 |
| Bihar | Supaul | 1,199,185 | 3,761,908 | 1,131,147 | 1,883,452 |
| Bihar | Vaishali | 1,533,823 | 4,811,684 | 1,446,741 | 2,411,622 |
| Chandigarh | Chandigarh | 202,732 | 773,174 | 190,612 | 345,191 |
| Chhattisgarh | Bastar | 505,197 | 1,410,518 | 475,104 | 779,726 |
| Chhattisgarh | Bijapur | 49,511 | 254,751 | 46,521 | 113,290 |
| Chhattisgarh | Bilaspur | 932,185 | 3,863,186 | 875,893 | 1,806,837 |
| Chhattisgarh | Dakshin Bastar Dantewada | 218,532 | 1,124,417 | 205,618 | 508,185 |
| Chhattisgarh | Dhamtari | 215,452 | 892,883 | 202,442 | 417,607 |
| Chhattisgarh | Durg | 872,399 | 2,879,849 | 819,717 | 1,563,767 |
| Chhattisgarh | Janjgir - Champa | 444,453 | 1,240,920 | 418,176 | 689,332 |
| Chhattisgarh | Jashpur | 260,381 | 1,339,742 | 245,741 | 630,520 |
| Chhattisgarh | Kabirdham | 295,214 | 824,241 | 278,190 | 465,057 |
| Chhattisgarh | Korba | 410,913 | 2,114,283 | 386,237 | 945,176 |
| Chhattisgarh | Korea (Koriya) | 220,274 | 1,133,380 | 207,286 | 513,136 |
| Chhattisgarh | Mahasamund | 385,372 | 1,369,991 | 362,309 | 553,167 |
| Chhattisgarh | Narayanpur | 34,394 | 176,966 | 32,435 | 82,347 |
| Chhattisgarh | Raigarh | 463,031 | 2,804,916 | 435,535 | 1,277,035 |
| Chhattisgarh | Raipur | 1,318,507 | 3,976,037 | 1,239,781 | 1,983,076 |
| Chhattisgarh | Rajnandgaon | 424,395 | 2,570,872 | 398,857 | 1,154,731 |
| Chhattisgarh | Surguja | 865,208 | 4,451,778 | 814,745 | 2,044,251 |
| Chhattisgarh | Uttar Bastar Kanker | 213,358 | 758,486 | 200,548 | 305,685 |
| Dadra and Nagar Haveli | Dadra & Nagar Haveli | 82,086 | 422,357 | 77,565 | 201,853 |
| Daman and Diu | Daman | 29,098 | 149,716 | 27,628 | 75,862 |
| Daman and Diu | Diu | 8,960 | 44,283 | 8,435 | 18,577 |
| Delhi | Central | 81,305 | 304,150 | 76,631 | 140,494 |
| Delhi | East | 335,860 | 1,053,610 | 316,425 | 521,074 |
| Delhi | New Delhi | 17,003 | 84,038 | 16,014 | 35,661 |
| Delhi | North | 198,678 | 1,203,539 | 187,527 | 576,284 |
| Delhi | North East | 702,586 | 2,118,691 | 663,970 | 1,104,491 |
| Delhi | North West | 735,606 | 1,530,110 | 691,896 | 966,354 |
| Delhi | South | 793,584 | 2,393,102 | 748,645 | 1,231,023 |
| Delhi | South West | 1,180,687 | 4,416,798 | 1,109,388 | 1,987,270 |
| Delhi | West | 383,416 | 2,318,904 | 360,756 | 1,016,344 |
| Goa | North Goa | 170,210 | 606,336 | 159,932 | 300,137 |
| Goa | South Goa | 159,257 | 884,439 | 149,640 | 398,074 |
| Gujarat | Ahmadabad | 1,638,287 | 5,139,394 | 1,549,771 | 2,624,541 |
| Gujarat | Amreli | 192,502 | 734,158 | 181,949 | 345,353 |
| Gujarat | Anand | 497,943 | 3,016,408 | 469,081 | 1,401,609 |
| Gujarat | Banaskantha | 918,374 | 5,563,264 | 879,470 | 3,164,463 |
| Gujarat | Bharuch | 420,713 | 1,604,501 | 396,949 | 733,434 |
| Gujarat | Bhavnagar | 436,453 | 1,632,715 | 415,122 | 817,759 |
| Gujarat | Dohad | 703,984 | 3,622,226 | 680,125 | 2,199,649 |
| Gujarat | Gandhinagar | 391,183 | 1,066,049 | 371,510 | 642,316 |
| Gujarat | Jamnagar | 536,320 | 2,006,303 | 504,427 | 911,668 |
| Gujarat | Junagadh | 409,861 | 1,144,336 | 387,126 | 653,780 |
| Gujarat | Kachchh | 454,992 | 1,617,492 | 431,793 | 702,967 |
| Gujarat | Kheda | 665,406 | 2,757,593 | 635,610 | 1,526,468 |
| Gujarat | Mahesana | 562,157 | 2,892,482 | 530,010 | 1,337,320 |
| Gujarat | Narmada | 174,920 | 527,482 | 164,652 | 264,201 |
| Gujarat | Navsari | 299,725 | 623,449 | 282,007 | 394,285 |
| Gujarat | Panchmahal | 556,213 | 3,369,390 | 536,634 | 2,081,492 |
| Gujarat | Patan | 292,293 | 1,770,631 | 280,629 | 1,034,157 |
| Gujarat | Porbandar | 88,403 | 330,704 | 83,226 | 151,699 |
| Gujarat | Rajkot | 809,734 | 2,260,789 | 763,066 | 1,276,420 |
| Gujarat | Sabarkantha | 665,729 | 3,425,390 | 628,509 | 1,604,328 |
| Gujarat | Surat | 1,474,363 | 7,586,076 | 1,396,750 | 3,707,546 |
| Gujarat | Surendranagar | 440,904 | 1,201,550 | 422,363 | 767,974 |
| Gujarat | Tapi | 168,983 | 869,470 | 158,940 | 392,839 |
| Gujarat | The Dangs | 68,949 | 354,766 | 65,264 | 172,206 |
| Gujarat | Vadodara | 995,247 | 3,723,092 | 938,747 | 1,751,070 |
| Gujarat | Valsad | 382,550 | 1,192,178 | 361,143 | 582,363 |
| Haryana | Ambala | 248,502 | 693,820 | 233,495 | 380,224 |
| Haryana | Bhiwani | 508,907 | 1,420,876 | 480,012 | 799,626 |
| Haryana | Faridabad | 748,992 | 3,853,808 | 714,124 | 2,016,420 |
| Haryana | Fatehabad | 317,862 | 1,189,080 | 298,694 | 535,550 |
| Haryana | Gurgaon | 595,158 | 3,062,283 | 566,682 | 1,601,187 |
| Haryana | Hisar | 401,113 | 1,209,582 | 377,452 | 603,799 |
| Haryana | Jhajjar | 248,465 | 1,278,434 | 234,916 | 605,389 |
| Haryana | Jind | 453,793 | 2,334,912 | 426,390 | 1,038,360 |
| Haryana | Kaithal | 319,025 | 1,641,485 | 299,760 | 729,986 |
| Haryana | Karnal | 531,858 | 1,603,851 | 499,741 | 794,393 |
| Haryana | Kurukshetra | 273,939 | 569,812 | 257,399 | 357,262 |
| Haryana | Mahendragarh | 222,442 | 1,144,534 | 209,093 | 511,996 |
| Haryana | Mewat | 688,609 | 3,543,118 | 672,085 | 2,345,851 |
| Haryana | Palwal | 390,042 | 2,006,892 | 376,261 | 1,194,797 |
| Haryana | Panchkula | 135,165 | 424,020 | 127,003 | 205,826 |
| Haryana | Panipat | 233,989 | 772,415 | 220,191 | 424,831 |
| Haryana | Rewari | 232,213 | 1,194,810 | 220,052 | 590,079 |
| Haryana | Rohtak | 466,137 | 1,301,462 | 438,942 | 727,219 |
| Haryana | Sirsa | 352,999 | 1,100,087 | 331,922 | 520,483 |
| Haryana | Sonipat | 342,800 | 1,763,818 | 322,750 | 807,547 |
| Haryana | Yamunanagar | 215,196 | 600,831 | 202,201 | 329,264 |
| Himachal Pradesh | Bilaspur | 86,083 | 328,299 | 81,317 | 154,405 |
| Himachal Pradesh | Chamba | 131,614 | 664,886 | 124,340 | 281,507 |
| Himachal Pradesh | Hamirpur | 131,540 | 434,221 | 124,614 | 255,892 |
| Himachal Pradesh | Kangra | 328,457 | 1,228,716 | 309,003 | 557,471 |
| Himachal Pradesh | Kinnaur | 12,917 | 36,064 | 12,150 | 19,987 |
| Himachal Pradesh | Kullu | 110,842 | 302,066 | 104,354 | 170,410 |
| Himachal Pradesh | Lahul and Spiti | 4,444 | 22,867 | 4,193 | 10,785 |
| Himachal Pradesh | Mandi | 269,167 | 838,833 | 253,164 | 397,873 |
| Himachal Pradesh | Shimla | 158,915 | 606,066 | 149,407 | 270,451 |
| Himachal Pradesh | Sirmaur | 106,234 | 331,069 | 99,959 | 157,607 |
| Himachal Pradesh | Solan | 145,409 | 543,955 | 136,985 | 251,967 |
| Himachal Pradesh | Una | 160,672 | 811,680 | 151,524 | 334,811 |
| Jammu and Kashmir | Anantnag | 254,918 | 1,056,437 | 239,625 | 496,802 |
| Jammu and Kashmir | Badgam | 162,388 | 607,471 | 152,599 | 273,681 |
| Jammu and Kashmir | Bandipore | 94,207 | 263,026 | 88,758 | 148,286 |
| Jammu and Kashmir | Baramula | 235,691 | 778,033 | 221,540 | 424,364 |
| Jammu and Kashmir | Doda | 108,157 | 357,034 | 103,249 | 225,858 |
| Jammu and Kashmir | Ganderbal | 83,450 | 345,835 | 78,412 | 161,781 |
| Jammu and Kashmir | Jammu | 307,773 | 1,023,083 | 289,786 | 497,204 |
| Jammu and Kashmir | Kargil | 27,489 | 141,439 | 25,831 | 62,991 |
| Jammu and Kashmir | Kathua | 154,276 | 588,373 | 144,960 | 260,794 |
| Jammu and Kashmir | Kishtwar | 78,921 | 247,579 | 74,342 | 122,365 |
| Jammu and Kashmir | Kulgam | 127,457 | 399,840 | 119,760 | 194,089 |
| Jammu and Kashmir | Kupwara | 190,154 | 596,523 | 178,730 | 290,247 |
| Jammu and Kashmir | Leh | 25,646 | 131,958 | 24,102 | 58,833 |
| Jammu and Kashmir | Pulwama | 117,970 | 321,490 | 110,846 | 177,922 |
| Jammu and Kashmir | Punch | 189,012 | 569,978 | 177,598 | 282,312 |
| Jammu and Kashmir | Rajouri | 232,455 | 1,196,056 | 220,862 | 597,843 |
| Jammu and Kashmir | Ramban | 99,282 | 327,738 | 94,113 | 192,800 |
| Jammu and Kashmir | Reasi | 109,466 | 563,236 | 103,118 | 255,404 |
| Jammu and Kashmir | Samba | 82,347 | 423,701 | 78,114 | 212,126 |
| Jammu and Kashmir | Shupiyan | 71,310 | 366,912 | 67,074 | 165,779 |
| Jammu and Kashmir | Srinagar | 237,973 | 890,225 | 223,602 | 400,543 |
| Jammu and Kashmir | Udhampur | 141,717 | 530,143 | 133,316 | 239,882 |
| Jharkhand | Bokaro | 470,211 | 2,419,390 | 443,351 | 1,125,824 |
| Jharkhand | Chatra | 511,496 | 2,631,814 | 487,533 | 1,381,816 |
| Jharkhand | Deoghar | 639,315 | 1,784,978 | 603,265 | 1,011,756 |
| Jharkhand | Dhanbad | 674,231 | 3,469,135 | 635,691 | 1,605,918 |
| Jharkhand | Dumka | 406,915 | 2,093,709 | 383,040 | 952,543 |
| Jharkhand | Garhwa | 634,708 | 3,265,778 | 602,005 | 1,628,547 |
| Jharkhand | Giridih | 964,236 | 4,961,309 | 912,447 | 2,418,062 |
| Jharkhand | Godda | 485,133 | 2,496,167 | 457,901 | 1,167,393 |
| Jharkhand | Gumla | 299,178 | 1,539,370 | 282,726 | 738,654 |
| Jharkhand | Hazaribagh | 653,829 | 1,825,500 | 615,406 | 1,017,640 |
| Jharkhand | Jamtara | 364,094 | 1,873,383 | 344,295 | 892,206 |
| Jharkhand | Khunti | 147,183 | 757,303 | 138,414 | 341,054 |
| Jharkhand | Kodarma | 280,548 | 1,699,484 | 264,200 | 781,914 |
| Jharkhand | Latehar | 218,100 | 1,122,198 | 207,173 | 568,778 |
| Jharkhand | Lohardaga | 133,975 | 689,343 | 126,388 | 318,501 |
| Jharkhand | Pakur | 349,780 | 1,799,734 | 329,354 | 825,451 |
| Jharkhand | Palamu | 797,187 | 4,101,784 | 754,655 | 1,995,985 |
| Jharkhand | Pashchimi Singhbhum | 420,930 | 2,165,820 | 399,665 | 1,083,467 |
| Jharkhand | Purbi Singhbhum | 549,924 | 2,829,535 | 517,126 | 1,273,028 |
| Jharkhand | Ramgarh | 243,251 | 1,251,604 | 229,070 | 574,985 |
| Jharkhand | Ranchi | 821,669 | 4,227,752 | 773,634 | 1,927,279 |
| Jharkhand | Sahibganj | 372,946 | 1,918,930 | 352,761 | 920,339 |
| Jharkhand | Saraikela Kharsawan | 439,874 | 2,263,292 | 414,429 | 1,046,630 |
| Jharkhand | Simdega | 179,215 | 922,118 | 169,214 | 437,788 |
| Karnataka | Bagalkot | 557,551 | 1,840,515 | 524,279 | 1,008,563 |
| Karnataka | Bangalore | 2,990,181 | 9,017,072 | 2,839,704 | 4,889,425 |
| Karnataka | Bangalore Rural | 217,304 | 1,118,099 | 204,871 | 515,707 |
| Karnataka | Belgaum | 1,254,063 | 3,781,704 | 1,180,841 | 1,903,259 |
| Karnataka | Bellary | 790,230 | 3,992,065 | 743,313 | 1,612,938 |
| Karnataka | Bidar | 379,120 | 1,058,508 | 357,503 | 596,505 |
| Karnataka | Bijapur | 488,401 | 1,472,804 | 463,098 | 780,815 |
| Karnataka | Chamarajanagar | 223,175 | 1,148,309 | 210,482 | 534,348 |
| Karnataka | Chikkaballapura | 225,792 | 1,161,776 | 213,109 | 538,588 |
| Karnataka | Chikmagalur | 198,950 | 1,023,660 | 187,838 | 484,684 |
| Karnataka | Chitradurga | 337,016 | 940,955 | 318,991 | 542,081 |
| Karnataka | Dakshina Kannada | 364,337 | 2,207,054 | 342,974 | 1,018,598 |
| Karnataka | Davanagere | 567,730 | 2,868,045 | 533,577 | 1,145,564 |
| Karnataka | Dharwad | 407,193 | 2,466,664 | 383,570 | 1,140,214 |
| Karnataka | Gadag | 237,238 | 646,519 | 224,381 | 373,635 |
| Karnataka | Gulbarga | 695,059 | 2,470,928 | 654,907 | 1,014,464 |
| Karnataka | Hassan | 373,846 | 1,923,558 | 351,801 | 870,204 |
| Karnataka | Haveri | 367,480 | 1,522,919 | 346,610 | 748,903 |
| Karnataka | Kodagu | 143,786 | 739,826 | 135,416 | 334,855 |
| Karnataka | Kolar | 357,464 | 998,044 | 336,117 | 550,846 |
| Karnataka | Koppal | 364,100 | 1,362,051 | 342,409 | 618,931 |
| Karnataka | Mandya | 480,752 | 2,473,627 | 453,210 | 1,150,610 |
| Karnataka | Mysore | 830,211 | 4,271,704 | 788,251 | 2,149,243 |
| Karnataka | Raichur | 388,247 | 1,480,685 | 366,134 | 682,390 |
| Karnataka | Ramanagara | 229,140 | 1,178,998 | 215,988 | 550,323 |
| Karnataka | Shimoga | 338,524 | 1,741,815 | 320,750 | 839,077 |
| Karnataka | Tumkur | 467,114 | 2,403,452 | 440,439 | 1,108,891 |
| Karnataka | Udupi | 181,748 | 693,145 | 171,146 | 315,102 |
| Karnataka | Uttara Kannada | 317,166 | 1,186,476 | 298,446 | 541,964 |
| Karnataka | Yadgir | 339,635 | 1,678,624 | 320,963 | 739,388 |
| Kerala | Alappuzha | 253,258 | 1,406,476 | 237,964 | 633,037 |
| Kerala | Ernakulam | 389,419 | 1,285,500 | 366,549 | 706,611 |
| Kerala | Idukki | 162,782 | 443,612 | 152,952 | 245,507 |
| Kerala | Kannur | 600,763 | 2,600,639 | 564,517 | 1,125,859 |
| Kerala | Kasaragod | 277,165 | 1,057,042 | 260,454 | 469,078 |
| Kerala | Kollam | 398,904 | 1,087,090 | 374,890 | 602,801 |
| Kerala | Kottayam | 434,227 | 1,726,733 | 408,005 | 765,686 |
| Kerala | Kozhikode | 615,011 | 2,548,746 | 579,088 | 1,213,318 |
| Kerala | Malappuram | 929,996 | 2,596,562 | 876,059 | 1,448,016 |
| Kerala | Palakkad | 683,625 | 2,607,187 | 642,343 | 1,155,625 |
| Kerala | Pathanamthitta | 219,322 | 1,326,465 | 206,078 | 568,997 |
| Kerala | Thiruvananthapuram | 550,631 | 1,145,351 | 517,643 | 721,084 |
| Kerala | Thrissur | 439,003 | 1,674,256 | 412,493 | 742,107 |
| Kerala | Wayanad | 165,489 | 1,002,485 | 155,793 | 459,812 |
| Lakshadweep | Lakshadweep | 17,448 | 68,874 | 16,394 | 30,894 |
| Madhya Pradesh | Alirajpur | 305,502 | 1,571,907 | 293,635 | 909,073 |
| Madhya Pradesh | Anuppur | 229,519 | 1,180,948 | 216,404 | 552,502 |
| Madhya Pradesh | Ashoknagar | 227,460 | 1,170,358 | 217,534 | 635,310 |
| Madhya Pradesh | Balaghat | 599,802 | 3,086,178 | 564,523 | 1,401,540 |
| Madhya Pradesh | Barwani | 653,685 | 3,363,422 | 625,266 | 1,835,578 |
| Madhya Pradesh | Betul | 505,589 | 2,601,422 | 475,877 | 1,186,260 |
| Madhya Pradesh | Bhind | 585,833 | 3,548,818 | 553,301 | 1,703,230 |
| Madhya Pradesh | Bhopal | 695,974 | 1,943,170 | 655,208 | 1,085,536 |
| Madhya Pradesh | Burhanpur | 246,869 | 1,270,220 | 234,523 | 640,719 |
| Madhya Pradesh | Chhatarpur | 544,796 | 2,803,150 | 518,467 | 1,447,208 |
| Madhya Pradesh | Chhindwara | 752,946 | 3,874,150 | 708,662 | 1,765,820 |
| Madhya Pradesh | Damoh | 401,634 | 2,066,539 | 379,524 | 982,879 |
| Madhya Pradesh | Datia | 229,887 | 1,392,592 | 217,476 | 683,090 |
| Madhya Pradesh | Dewas | 514,231 | 1,697,513 | 485,120 | 954,684 |
| Madhya Pradesh | Dhar | 734,423 | 2,050,520 | 691,405 | 1,140,314 |
| Madhya Pradesh | Dindori | 233,795 | 1,202,952 | 220,803 | 572,739 |
| Madhya Pradesh | Guna | 540,142 | 1,628,831 | 510,222 | 843,082 |
| Madhya Pradesh | Gwalior | 606,551 | 1,693,499 | 573,624 | 979,955 |
| Madhya Pradesh | Harda | 170,140 | 513,067 | 160,883 | 269,871 |
| Madhya Pradesh | Hoshangabad | 349,350 | 1,797,522 | 330,303 | 861,702 |
| Madhya Pradesh | Indore | 971,065 | 2,711,227 | 917,210 | 1,556,426 |
| Madhya Pradesh | Jabalpur | 682,701 | 1,906,110 | 642,547 | 1,061,714 |
| Madhya Pradesh | Jhabua | 425,112 | 2,187,338 | 406,357 | 1,200,979 |
| Madhya Pradesh | Katni | 397,131 | 2,043,370 | 374,482 | 951,963 |
| Madhya Pradesh | Khandwa (East Nimar) | 408,130 | 2,472,342 | 384,625 | 1,162,251 |
| Madhya Pradesh | Khargone (West Nimar) | 557,053 | 1,679,826 | 526,084 | 872,003 |
| Madhya Pradesh | Mandla | 282,492 | 1,453,514 | 266,092 | 670,757 |
| Madhya Pradesh | Mandsaur | 493,276 | 1,628,338 | 470,197 | 1,010,864 |
| Madhya Pradesh | Morena | 561,096 | 1,529,096 | 530,920 | 897,576 |
| Madhya Pradesh | Narsimhapur | 284,598 | 1,464,350 | 268,546 | 678,049 |
| Madhya Pradesh | Neemuch | 299,775 | 836,976 | 284,270 | 495,109 |
| Madhya Pradesh | Panna | 265,019 | 1,363,608 | 255,382 | 798,173 |
| Madhya Pradesh | Raisen | 455,086 | 1,617,824 | 427,801 | 652,552 |
| Madhya Pradesh | Rajgarh | 599,121 | 3,082,671 | 567,733 | 1,505,482 |
| Madhya Pradesh | Ratlam | 420,064 | 2,161,368 | 398,364 | 1,068,118 |
| Madhya Pradesh | Rewa | 751,055 | 3,864,424 | 708,951 | 1,817,435 |
| Madhya Pradesh | Sagar | 678,332 | 3,490,240 | 645,871 | 1,820,858 |
| Madhya Pradesh | Satna | 588,611 | 3,028,592 | 555,234 | 1,413,937 |
| Madhya Pradesh | Sehore | 347,735 | 970,880 | 327,668 | 545,820 |
| Madhya Pradesh | Seoni | 347,253 | 1,786,730 | 327,036 | 821,581 |
| Madhya Pradesh | Shahdol | 368,653 | 1,896,841 | 348,966 | 928,239 |
| Madhya Pradesh | Shajapur | 416,313 | 1,162,351 | 392,197 | 654,025 |
| Madhya Pradesh | Sheopur | 231,008 | 1,188,611 | 218,644 | 582,090 |
| Madhya Pradesh | Shivpuri | 675,366 | 1,885,633 | 638,106 | 1,081,516 |
| Madhya Pradesh | Sidhi | 461,331 | 2,373,696 | 440,993 | 1,287,918 |
| Madhya Pradesh | Singrauli | 515,148 | 2,650,604 | 489,707 | 1,359,917 |
| Madhya Pradesh | Tikamgarh | 421,462 | 2,168,560 | 402,045 | 1,155,858 |
| Madhya Pradesh | Ujjain | 625,453 | 2,064,662 | 590,266 | 1,181,393 |
| Madhya Pradesh | Umaria | 267,309 | 1,375,394 | 251,712 | 628,025 |
| Madhya Pradesh | Vidisha | 458,638 | 2,359,840 | 438,169 | 1,276,444 |
| Maharashtra | Ahmadnagar | 1,353,760 | 6,965,532 | 1,283,113 | 3,453,484 |
| Maharashtra | Akola | 460,441 | 2,369,117 | 435,363 | 1,146,117 |
| Maharashtra | Amravati | 688,689 | 3,543,527 | 650,263 | 1,686,767 |
| Maharashtra | Aurangabad | 833,689 | 4,289,602 | 785,733 | 1,982,372 |
| Maharashtra | Bhandara | 213,106 | 1,096,498 | 200,372 | 489,901 |
| Maharashtra | Bid | 821,949 | 2,294,894 | 777,258 | 1,325,967 |
| Maharashtra | Buldana | 608,064 | 1,697,723 | 575,024 | 979,910 |
| Maharashtra | Chandrapur | 430,172 | 2,213,374 | 404,781 | 1,005,330 |
| Maharashtra | Dhule | 573,691 | 3,475,267 | 542,482 | 1,708,344 |
| Maharashtra | Gadchiroli | 187,444 | 964,460 | 176,260 | 431,847 |
| Maharashtra | Gondiya | 325,643 | 1,675,539 | 306,486 | 759,564 |
| Maharashtra | Hingoli | 365,196 | 1,019,633 | 344,332 | 577,230 |
| Maharashtra | Jalgaon | 869,525 | 5,267,351 | 826,294 | 2,784,542 |
| Maharashtra | Jalna | 513,709 | 1,434,282 | 484,016 | 807,660 |
| Maharashtra | Kolhapur | 831,248 | 2,506,679 | 786,730 | 1,320,956 |
| Maharashtra | Latur | 735,591 | 4,456,016 | 693,195 | 2,082,238 |
| Maharashtra | Mumbai | 714,361 | 3,608,789 | 680,064 | 1,670,554 |
| Maharashtra | Mumbai Suburban | 1,948,514 | 5,440,280 | 1,858,638 | 3,375,417 |
| Maharashtra | Nagpur | 967,134 | 5,858,642 | 908,974 | 2,633,198 |
| Maharashtra | Nanded | 1,192,353 | 7,222,957 | 1,127,511 | 3,531,578 |
| Maharashtra | Nandurbar | 486,919 | 2,505,354 | 468,747 | 1,484,456 |
| Maharashtra | Nashik | 2,459,547 | 8,743,659 | 2,326,781 | 3,705,392 |
| Maharashtra | Osmanabad | 358,712 | 2,172,978 | 338,735 | 1,043,695 |
| Maharashtra | Parbhani | 546,222 | 3,308,870 | 515,153 | 1,571,747 |
| Maharashtra | Pune | 2,359,309 | 7,788,240 | 2,217,594 | 4,246,499 |
| Maharashtra | Raigarh | 840,485 | 4,324,568 | 792,267 | 2,014,736 |
| Maharashtra | Ratnagiri | 398,649 | 1,491,294 | 375,454 | 690,022 |
| Maharashtra | Sangli | 708,383 | 3,644,860 | 670,926 | 1,802,759 |
| Maharashtra | Satara | 880,313 | 3,129,502 | 831,557 | 1,311,809 |
| Maharashtra | Sindhudurg | 113,136 | 431,474 | 106,389 | 193,096 |
| Maharashtra | Solapur | 1,209,142 | 3,295,147 | 1,138,972 | 1,864,641 |
| Maharashtra | Thane | 3,511,069 | 10,587,843 | 3,344,789 | 5,879,404 |
| Maharashtra | Wardha | 220,252 | 1,334,230 | 207,253 | 606,476 |
| Maharashtra | Washim | 255,588 | 713,605 | 240,650 | 395,174 |
| Maharashtra | Yavatmal | 828,384 | 4,262,306 | 779,705 | 1,938,658 |
| Manipur | Bishnupur | 50,561 | 306,284 | 47,749 | 146,886 |
| Manipur | Chandel | 40,887 | 114,157 | 39,035 | 70,698 |
| Manipur | Churachandpur | 72,694 | 198,104 | 69,332 | 123,314 |
| Manipur | Imphal East | 133,970 | 420,273 | 126,591 | 213,830 |
| Manipur | Imphal West | 154,429 | 577,701 | 145,589 | 269,533 |
| Manipur | Senapati (excluding 3 subdivisions) | 61,633 | 128,202 | 58,635 | 86,672 |
| Manipur | Tamenglong | 41,150 | 85,595 | 39,372 | 59,721 |
| Manipur | Thoubal | 125,961 | 763,039 | 119,435 | 383,165 |
| Manipur | Ukhrul | 49,178 | 248,436 | 47,255 | 124,012 |
| Meghalaya | East Garo Hills | 140,094 | 391,145 | 135,029 | 261,043 |
| Meghalaya | East Khasi Hills | 279,609 | 1,030,694 | 264,575 | 480,183 |
| Meghalaya | Jaintia Hills | 174,755 | 622,524 | 165,445 | 331,724 |
| Meghalaya | Ribhoi | 96,220 | 475,562 | 91,438 | 222,505 |
| Meghalaya | South Garo Hills | 25,969 | 133,621 | 24,425 | 59,888 |
| Meghalaya | West Garo Hills | 163,551 | 539,893 | 155,370 | 323,235 |
| Meghalaya | West Khasi Hills | 163,716 | 809,158 | 154,812 | 360,856 |
| Mizoram | Aizawl | 102,476 | 506,480 | 97,784 | 244,792 |
| Mizoram | Champhai | 42,877 | 116,847 | 41,085 | 75,598 |
| Mizoram | Kolasib | 20,972 | 79,982 | 20,196 | 44,017 |
| Mizoram | Lawngtlai | 33,434 | 172,028 | 32,262 | 102,301 |
| Mizoram | Lunglei | 36,630 | 76,192 | 35,045 | 53,516 |
| Mizoram | Mamit | 23,439 | 120,603 | 22,558 | 70,194 |
| Mizoram | Saiha | 16,713 | 45,546 | 15,915 | 28,178 |
| Mizoram | Serchhip | 14,010 | 70,774 | 13,323 | 32,204 |
| Nagaland | Dimapur | 95,988 | 268,000 | 92,291 | 174,681 |
| Nagaland | Kiphire | 28,036 | 76,404 | 27,068 | 52,074 |
| Nagaland | Kohima | 40,877 | 128,232 | 38,759 | 66,648 |
| Nagaland | Longleng | 11,658 | 70,624 | 11,344 | 47,134 |
| Nagaland | Mokokchung | 27,831 | 92,515 | 26,398 | 48,098 |
| Nagaland | Mon | 86,161 | 322,316 | 84,235 | 202,279 |
| Nagaland | Peren | 22,437 | 67,660 | 21,530 | 39,220 |
| Nagaland | Phek | 44,478 | 147,853 | 42,551 | 81,541 |
| Nagaland | Tuensang | 58,621 | 183,897 | 56,174 | 104,402 |
| Nagaland | Wokha | 16,051 | 52,986 | 15,516 | 37,711 |
| Nagaland | Zunheboto | 33,381 | 138,337 | 32,251 | 85,049 |
| Odisha | Anugul | 419,102 | 1,170,140 | 393,794 | 641,254 |
| Odisha | Balangir | 502,115 | 3,041,676 | 471,793 | 1,361,219 |
| Odisha | Baleshwar | 472,728 | 1,560,508 | 444,495 | 850,489 |
| Odisha | Bargarh | 436,019 | 1,217,372 | 409,967 | 670,398 |
| Odisha | Baudh | 135,309 | 377,784 | 127,138 | 207,031 |
| Odisha | Bhadrak | 448,234 | 1,676,784 | 421,225 | 755,645 |
| Odisha | Cuttack | 518,052 | 1,614,457 | 487,301 | 766,426 |
| Odisha | Debagarh | 83,137 | 427,768 | 78,226 | 193,306 |
| Odisha | Dhenkanal | 328,388 | 1,689,663 | 308,616 | 753,512 |
| Odisha | Gajapati | 156,646 | 488,170 | 149,719 | 261,792 |
| Odisha | Ganjam | 864,949 | 2,695,528 | 816,782 | 1,312,107 |
| Odisha | Jagatsinghapur | 248,312 | 778,968 | 233,382 | 379,165 |
| Odisha | Jajapur | 438,590 | 912,297 | 412,104 | 571,961 |
| Odisha | Jharsuguda | 143,013 | 399,295 | 134,460 | 220,171 |
| Odisha | Kalahandi | 415,447 | 1,371,419 | 390,458 | 746,963 |
| Odisha | Kandhamal | 227,085 | 941,093 | 213,726 | 446,807 |
| Odisha | Kendrapara | 428,048 | 1,166,513 | 403,486 | 662,644 |
| Odisha | Kendujhar | 650,794 | 2,313,565 | 612,256 | 936,172 |
| Odisha | Khordha | 544,655 | 1,484,291 | 513,884 | 848,435 |
| Odisha | Koraput | 416,054 | 2,140,731 | 393,837 | 1,030,482 |
| Odisha | Malkangiri | 282,228 | 851,075 | 265,454 | 424,369 |
| Odisha | Mayurbhanj | 643,876 | 3,312,953 | 607,952 | 1,573,124 |
| Odisha | Nabarangapur | 440,108 | 2,264,498 | 414,187 | 1,027,568 |
| Odisha | Nayagarh | 235,650 | 837,734 | 221,420 | 336,512 |
| Odisha | Nuapada | 208,336 | 1,071,956 | 196,008 | 483,378 |
| Odisha | Puri | 408,529 | 849,768 | 383,859 | 532,759 |
| Odisha | Rayagada | 329,388 | 1,026,504 | 310,413 | 490,186 |
| Odisha | Sambalpur | 297,591 | 927,412 | 280,035 | 440,670 |
| Odisha | Subarnapur | 177,882 | 496,650 | 167,140 | 272,172 |
| Odisha | Sundargarh | 478,449 | 1,579,393 | 449,663 | 860,075 |
| Puducherry | Karaikal | 46,939 | 284,342 | 44,104 | 127,249 |
| Puducherry | Mahe | 6,238 | 37,726 | 5,861 | 16,183 |
| Puducherry | Puducherry | 200,214 | 1,030,165 | 188,123 | 458,125 |
| Puducherry | Yanam | 12,382 | 75,006 | 11,635 | 33,578 |
| Punjab | Amritsar | 550,726 | 1,830,696 | 517,469 | 874,763 |
| Punjab | Barnala | 98,714 | 548,211 | 92,753 | 246,743 |
| Punjab | Bathinda | 233,839 | 1,155,734 | 219,718 | 477,110 |
| Punjab | Faridkot | 166,518 | 553,531 | 156,462 | 264,494 |
| Punjab | Fatehgarh Sahib | 133,243 | 658,544 | 125,198 | 271,889 |
| Punjab | Firozpur | 499,524 | 1,567,033 | 469,430 | 761,162 |
| Punjab | Gurdaspur | 450,840 | 1,498,662 | 423,615 | 716,107 |
| Punjab | Hoshiarpur | 246,654 | 940,679 | 231,759 | 416,952 |
| Punjab | Jalandhar | 477,779 | 2,361,388 | 448,927 | 974,828 |
| Punjab | Kapurthala | 143,980 | 623,274 | 135,285 | 269,628 |
| Punjab | Ludhiana | 635,668 | 2,377,950 | 598,597 | 1,086,736 |
| Punjab | Mansa | 169,064 | 644,772 | 158,855 | 285,793 |
| Punjab | Moga | 181,941 | 899,229 | 170,954 | 371,220 |
| Punjab | Muktsar | 182,918 | 791,831 | 171,872 | 342,545 |
| Punjab | Patiala | 490,161 | 1,629,369 | 460,561 | 778,563 |
| Punjab | Rupnagar | 136,202 | 541,614 | 127,977 | 240,168 |
| Punjab | Sahibzada Ajit Singh Nagar | 256,640 | 1,296,488 | 241,142 | 516,148 |
| Punjab | Sangrur | 119,563 | 471,964 | 112,362 | 212,128 |
| Punjab | Shahid Bhagat Singh Nagar | 433,401 | 1,621,299 | 407,668 | 735,191 |
| Punjab | Tarn Taran | 218,546 | 1,080,148 | 205,348 | 445,907 |
| Rajasthan | Ajmer | 680,201 | 1,853,678 | 640,808 | 1,045,778 |
| Rajasthan | Alwar | 1,129,780 | 3,154,363 | 1,074,676 | 1,926,334 |
| Rajasthan | Banswara | 700,158 | 3,602,541 | 662,164 | 1,731,364 |
| Rajasthan | Baran | 395,637 | 1,193,069 | 372,393 | 601,215 |
| Rajasthan | Barmer | 883,686 | 4,546,851 | 851,312 | 2,653,753 |
| Rajasthan | Bharatpur | 1,080,963 | 3,391,039 | 1,031,924 | 1,879,938 |
| Rajasthan | Bhilwara | 770,950 | 2,544,957 | 725,760 | 1,409,791 |
| Rajasthan | Bikaner | 725,578 | 2,395,184 | 688,995 | 1,443,437 |
| Rajasthan | Bundi | 317,167 | 1,186,482 | 299,423 | 560,712 |
| Rajasthan | Chittaurgarh | 369,703 | 1,220,416 | 350,615 | 730,636 |
| Rajasthan | Churu | 550,987 | 3,337,731 | 520,270 | 1,600,034 |
| Rajasthan | Dausa | 460,477 | 1,285,659 | 435,011 | 738,991 |
| Rajasthan | Dhaulpur | 438,147 | 1,670,990 | 414,680 | 790,294 |
| Rajasthan | Dungarpur | 518,459 | 1,447,546 | 489,143 | 822,723 |
| Rajasthan | Ganganagar | 654,505 | 2,712,417 | 615,095 | 1,271,677 |
| Rajasthan | Hanumangarh | 551,557 | 2,837,941 | 520,250 | 1,334,628 |
| Rajasthan | Jaipur | 1,669,716 | 10,114,697 | 1,575,815 | 4,838,018 |
| Rajasthan | Jaisalmer | 258,770 | 1,567,560 | 247,796 | 882,691 |
| Rajasthan | Jalor | 726,619 | 1,980,176 | 698,814 | 1,312,881 |
| Rajasthan | Jhalawar | 320,618 | 873,746 | 301,519 | 487,783 |
| Rajasthan | Jhunjhunun | 603,942 | 1,686,215 | 568,642 | 942,339 |
| Rajasthan | Jodhpur | 1,097,522 | 6,648,494 | 1,042,660 | 3,445,083 |
| Rajasthan | Karauli | 482,888 | 1,504,870 | 457,590 | 757,215 |
| Rajasthan | Kota | 508,653 | 1,533,873 | 479,020 | 773,221 |
| Rajasthan | Nagaur | 890,507 | 4,581,948 | 846,789 | 2,357,525 |
| Rajasthan | Pali | 598,582 | 2,239,218 | 567,117 | 1,086,769 |
| Rajasthan | Pratapgarh | 305,125 | 1,569,968 | 287,732 | 728,027 |
| Rajasthan | Rajsamand | 350,728 | 729,539 | 331,253 | 472,881 |
| Rajasthan | Sawai Madhopur | 499,622 | 2,523,979 | 475,245 | 1,150,872 |
| Rajasthan | Sikar | 828,658 | 2,313,626 | 783,974 | 1,347,626 |
| Rajasthan | Sirohi | 435,415 | 1,186,590 | 415,514 | 740,778 |
| Rajasthan | Tonk | 394,312 | 1,503,814 | 370,738 | 671,398 |
| Rajasthan | Udaipur | 1,020,022 | 5,248,343 | 972,561 | 2,765,752 |
| Sikkim | East District | 41,507 | 137,018 | 39,006 | 74,528 |
| Sikkim | North District | 5,954 | 30,634 | 5,595 | 13,634 |
| Sikkim | South District | 21,819 | 112,268 | 20,502 | 49,927 |
| Sikkim | West District | 16,027 | 82,464 | 15,064 | 36,855 |
| Tamil Nadu | Ariyalur | 163,204 | 839,735 | 153,949 | 390,264 |
| Tamil Nadu | Chennai | 853,399 | 4,391,016 | 801,865 | 1,952,731 |
| Tamil Nadu | Coimbatore | 999,721 | 5,143,890 | 939,640 | 2,297,920 |
| Tamil Nadu | Cuddalore | 644,245 | 3,314,851 | 607,024 | 1,515,988 |
| Tamil Nadu | Dharmapuri | 422,371 | 2,173,235 | 398,336 | 1,008,110 |
| Tamil Nadu | Dindigul | 836,413 | 4,303,615 | 786,039 | 1,918,702 |
| Tamil Nadu | Erode | 813,732 | 2,892,808 | 764,647 | 1,162,343 |
| Tamil Nadu | Kancheepuram | 887,350 | 4,565,702 | 837,324 | 2,102,505 |
| Tamil Nadu | Kanniyakumari | 613,165 | 3,154,930 | 578,670 | 1,474,408 |
| Tamil Nadu | Karur | 388,342 | 1,084,256 | 364,891 | 594,189 |
| Tamil Nadu | Krishnagiri | 589,545 | 3,033,401 | 554,286 | 1,362,060 |
| Tamil Nadu | Madurai | 606,498 | 3,120,630 | 572,223 | 1,452,443 |
| Tamil Nadu | Nagapattinam | 431,042 | 2,217,853 | 411,487 | 1,173,055 |
| Tamil Nadu | Namakkal | 388,992 | 2,001,488 | 366,485 | 915,347 |
| Tamil Nadu | Perambalur | 169,387 | 871,549 | 159,601 | 400,471 |
| Tamil Nadu | Pudukkottai | 402,561 | 2,071,306 | 380,526 | 986,573 |
| Tamil Nadu | Ramanathapuram | 360,420 | 1,854,480 | 340,064 | 873,040 |
| Tamil Nadu | Salem | 1,315,262 | 6,767,448 | 1,237,382 | 3,060,803 |
| Tamil Nadu | Sivaganga | 286,183 | 1,733,619 | 269,438 | 801,883 |
| Tamil Nadu | Thanjavur | 679,532 | 1,897,263 | 638,690 | 1,042,874 |
| Tamil Nadu | The Nilgiris | 183,628 | 944,826 | 172,572 | 421,168 |
| Tamil Nadu | Theni | 335,736 | 1,727,470 | 316,872 | 808,643 |
| Tamil Nadu | Thiruvallur | 878,155 | 4,518,394 | 825,662 | 2,025,590 |
| Tamil Nadu | Thiruvarur | 438,615 | 2,256,816 | 412,622 | 1,022,492 |
| Tamil Nadu | Thoothukkudi | 317,043 | 885,188 | 300,577 | 526,380 |
| Tamil Nadu | Tiruchirappalli | 751,925 | 3,868,897 | 707,768 | 1,766,703 |
| Tamil Nadu | Tirunelveli | 731,800 | 2,043,197 | 689,705 | 1,155,826 |
| Tamil Nadu | Tiruppur | 836,464 | 4,303,880 | 785,952 | 1,913,980 |
| Tamil Nadu | Tiruvannamalai | 676,726 | 3,481,976 | 637,815 | 1,594,284 |
| Tamil Nadu | Vellore | 1,426,698 | 7,340,823 | 1,342,449 | 3,307,375 |
| Tamil Nadu | Viluppuram | 1,028,703 | 5,293,010 | 968,429 | 2,390,279 |
| Tamil Nadu | Virudhunagar | 538,497 | 2,770,740 | 509,830 | 1,362,297 |
| Telangana | Adilabad | 627,858 | 1,305,988 | 591,298 | 829,500 |
| Telangana | Hyderabad | 2,015,240 | 10,369,062 | 1,895,969 | 4,686,254 |
| Telangana | Karimnagar | 914,323 | 4,704,489 | 859,113 | 2,092,250 |
| Telangana | Khammam | 743,655 | 2,076,295 | 699,745 | 1,154,341 |
| Telangana | Mahbubnagar | 1,021,070 | 5,253,736 | 963,529 | 2,476,008 |
| Telangana | Medak | 717,311 | 3,690,800 | 673,995 | 1,641,337 |
| Telangana | Nalgonda | 837,964 | 4,311,597 | 789,023 | 1,969,938 |
| Telangana | Nizamabad | 839,870 | 2,344,928 | 790,298 | 1,303,704 |
| Telangana | Rangareddy | 1,954,938 | 10,058,792 | 1,838,968 | 4,547,984 |
| Telangana | Warangal | 941,510 | 4,844,376 | 886,898 | 2,236,312 |
| Tripura | Dhalai | 77,175 | 397,090 | 73,823 | 216,866 |
| Tripura | North Tripura | 183,024 | 551,921 | 174,751 | 309,729 |
| Tripura | South Tripura | 152,144 | 458,800 | 145,008 | 254,030 |
| Tripura | West Tripura | 387,187 | 1,081,033 | 367,086 | 636,605 |
| Uttar Pradesh | Agra | 1,449,225 | 4,370,225 | 1,370,588 | 2,289,824 |
| Uttar Pradesh | Aligarh | 1,325,821 | 4,131,790 | 1,251,790 | 2,021,930 |
| Uttar Pradesh | Allahabad | 1,923,616 | 11,652,749 | 1,830,661 | 6,113,631 |
| Uttar Pradesh | Ambedkar Nagar | 807,915 | 4,156,983 | 764,904 | 2,030,361 |
| Uttar Pradesh | Auraiya | 419,699 | 2,159,489 | 402,323 | 1,213,284 |
| Uttar Pradesh | Azamgarh | 1,060,361 | 6,423,383 | 1,011,931 | 3,463,799 |
| Uttar Pradesh | Baghpat | 439,342 | 1,378,239 | 414,373 | 687,124 |
| Uttar Pradesh | Bahraich | 1,490,711 | 7,670,190 | 1,467,652 | 5,496,928 |
| Uttar Pradesh | Ballia | 1,001,004 | 2,794,818 | 953,714 | 1,702,454 |
| Uttar Pradesh | Balrampur | 808,487 | 2,257,307 | 790,873 | 1,675,705 |
| Uttar Pradesh | Banda | 361,497 | 1,860,021 | 343,259 | 940,043 |
| Uttar Pradesh | Bara Banki | 884,591 | 4,551,506 | 842,985 | 2,370,850 |
| Uttar Pradesh | Bareilly | 1,359,824 | 4,100,632 | 1,294,045 | 2,235,788 |
| Uttar Pradesh | Basti | 775,611 | 2,113,691 | 733,620 | 1,237,057 |
| Uttar Pradesh | Bijnor | 1,115,770 | 6,759,039 | 1,051,041 | 3,143,272 |
| Uttar Pradesh | Budaun | 1,448,328 | 8,773,584 | 1,376,246 | 4,516,416 |
| Uttar Pradesh | Bulandshahr | 1,139,332 | 4,721,651 | 1,079,236 | 2,407,828 |
| Uttar Pradesh | Chandauli | 701,023 | 3,606,990 | 663,706 | 1,761,881 |
| Uttar Pradesh | Chitrakoot | 326,302 | 1,678,931 | 307,623 | 776,192 |
| Uttar Pradesh | Deoria | 905,222 | 2,839,731 | 855,550 | 1,440,834 |
| Uttar Pradesh | Etah | 611,236 | 2,172,936 | 581,960 | 958,021 |
| Uttar Pradesh | Etawah | 458,863 | 2,361,002 | 436,144 | 1,199,538 |
| Uttar Pradesh | Faizabad | 620,419 | 3,192,255 | 589,222 | 1,592,493 |
| Uttar Pradesh | Farrukhabad | 610,815 | 1,705,405 | 583,412 | 1,061,466 |
| Uttar Pradesh | Fatehpur | 684,970 | 3,524,393 | 650,842 | 1,790,502 |
| Uttar Pradesh | Firozabad | 825,121 | 2,303,749 | 780,898 | 1,336,816 |
| Uttar Pradesh | Gautam Buddha Nagar | 713,796 | 3,672,713 | 675,362 | 1,767,283 |
| Uttar Pradesh | Ghaziabad | 1,467,824 | 7,552,433 | 1,391,234 | 3,732,648 |
| Uttar Pradesh | Ghazipur | 986,417 | 2,754,092 | 937,093 | 1,654,926 |
| Uttar Pradesh | Gonda | 1,044,926 | 3,449,371 | 1,005,069 | 2,326,943 |
| Uttar Pradesh | Gorakhpur | 1,365,351 | 5,658,325 | 1,286,704 | 2,740,316 |
| Uttar Pradesh | Hamirpur | 199,403 | 1,025,995 | 189,980 | 539,627 |
| Uttar Pradesh | Hardoi | 1,152,089 | 5,927,874 | 1,099,118 | 3,128,785 |
| Uttar Pradesh | Jalaun | 360,389 | 1,854,318 | 342,925 | 957,124 |
| Uttar Pradesh | Jaunpur | 1,355,965 | 4,476,132 | 1,288,837 | 2,720,292 |
| Uttar Pradesh | Jhansi | 464,944 | 1,298,129 | 438,647 | 734,491 |
| Uttar Pradesh | Jyotiba Phule Nagar | 623,343 | 3,776,046 | 588,759 | 1,816,846 |
| Uttar Pradesh | Kannauj | 520,665 | 1,453,704 | 495,715 | 886,791 |
| Uttar Pradesh | Kanpur Dehat | 529,470 | 2,724,294 | 500,941 | 1,310,644 |
| Uttar Pradesh | Kanpur Nagar | 1,256,601 | 6,465,623 | 1,190,151 | 3,138,440 |
| Uttar Pradesh | Kanshiram Nagar | 551,447 | 3,340,517 | 525,478 | 1,766,984 |
| Uttar Pradesh | Kaushambi | 463,913 | 2,386,985 | 442,103 | 1,246,860 |
| Uttar Pradesh | Kheri | 1,677,256 | 8,630,029 | 1,588,084 | 4,193,643 |
| Uttar Pradesh | Kushinagar | 1,252,785 | 5,191,823 | 1,195,378 | 2,833,730 |
| Uttar Pradesh | Lalitpur | 378,912 | 1,949,624 | 357,382 | 908,564 |
| Uttar Pradesh | Lucknow | 812,705 | 1,690,483 | 769,030 | 1,108,192 |
| Uttar Pradesh | Mahamaya Nagar | 538,017 | 1,776,030 | 510,591 | 1,058,454 |
| Uttar Pradesh | Mahoba | 203,767 | 568,920 | 192,502 | 325,711 |
| Uttar Pradesh | Mahrajganj | 798,354 | 4,836,213 | 759,317 | 2,522,137 |
| Uttar Pradesh | Mainpuri | 516,220 | 3,127,122 | 488,939 | 1,557,921 |
| Uttar Pradesh | Mathura | 986,900 | 2,689,493 | 936,633 | 1,615,720 |
| Uttar Pradesh | Mau | 714,081 | 1,993,724 | 678,625 | 1,201,621 |
| Uttar Pradesh | Meerut | 1,415,992 | 4,674,284 | 1,342,586 | 2,761,138 |
| Uttar Pradesh | Mirzapur | 872,534 | 4,489,474 | 826,609 | 2,201,706 |
| Uttar Pradesh | Moradabad | 1,628,247 | 5,788,397 | 1,550,119 | 2,569,161 |
| Uttar Pradesh | Muzaffarnagar | 1,745,898 | 5,763,329 | 1,653,758 | 3,373,458 |
| Uttar Pradesh | Pilibhit | 604,158 | 1,821,876 | 569,974 | 931,238 |
| Uttar Pradesh | Pratapgarh | 750,725 | 2,045,870 | 710,727 | 1,206,277 |
| Uttar Pradesh | Rae Bareli | 877,006 | 4,512,480 | 827,663 | 2,106,283 |
| Uttar Pradesh | Rampur | 871,689 | 2,433,769 | 822,392 | 1,374,064 |
| Uttar Pradesh | Saharanpur | 1,219,971 | 7,390,258 | 1,151,660 | 3,537,436 |
| Uttar Pradesh | Sant Kabir Nagar | 627,714 | 3,171,068 | 595,619 | 1,397,780 |
| Uttar Pradesh | Sant Ravidas Nagar (Bhadohi) | 643,406 | 2,123,926 | 613,269 | 1,324,576 |
| Uttar Pradesh | Shahjahanpur | 972,943 | 5,893,828 | 919,202 | 2,817,182 |
| Uttar Pradesh | Shrawasti | 531,166 | 2,733,022 | 517,163 | 1,773,823 |
| Uttar Pradesh | Siddharth Nagar | 829,022 | 3,161,696 | 795,948 | 1,699,594 |
| Uttar Pradesh | Sitapur | 1,706,680 | 8,781,423 | 1,619,651 | 4,403,916 |
| Uttar Pradesh | Sonbhadra | 707,976 | 3,642,766 | 676,852 | 1,970,554 |
| Uttar Pradesh | Sultanpur | 1,000,006 | 3,555,009 | 952,050 | 1,581,887 |
| Uttar Pradesh | Unnao | 905,745 | 2,528,854 | 858,840 | 1,484,878 |
| Uttar Pradesh | Varanasi | 1,047,183 | 2,923,749 | 989,937 | 1,680,491 |
| Uttarakhand | Almora | 127,969 | 478,717 | 120,634 | 222,062 |
| Uttarakhand | Bageshwar | 67,486 | 183,912 | 63,828 | 107,505 |
| Uttarakhand | Chamoli | 107,195 | 334,062 | 101,316 | 164,590 |
| Uttarakhand | Champawat | 64,157 | 324,108 | 60,420 | 132,408 |
| Uttarakhand | Dehradun | 469,311 | 1,415,236 | 442,041 | 717,184 |
| Uttarakhand | Garhwal | 156,564 | 472,127 | 147,548 | 239,960 |
| Uttarakhand | Hardwar | 707,110 | 3,638,310 | 671,483 | 1,829,344 |
| Uttarakhand | Nainital | 237,198 | 1,436,879 | 224,005 | 686,215 |
| Uttarakhand | Pithoragarh | 106,636 | 406,684 | 100,317 | 182,714 |
| Uttarakhand | Rudraprayag | 72,278 | 201,800 | 68,134 | 113,815 |
| Uttarakhand | Tehri Garhwal | 151,148 | 626,390 | 142,815 | 311,352 |
| Uttarakhand | Udham Singh Nagar | 452,342 | 2,327,449 | 428,134 | 1,131,869 |
| Uttarakhand | Uttarkashi | 86,195 | 240,659 | 81,218 | 135,584 |
| West Bengal | Bankura | 879,576 | 3,354,496 | 826,460 | 1,486,867 |
| West Bengal | Barddhaman | 2,006,951 | 7,980,773 | 1,888,705 | 3,604,682 |
| West Bengal | Birbhum | 1,057,166 | 3,954,722 | 993,327 | 1,779,366 |
| West Bengal | Dakshin Dinajpur | 421,683 | 1,608,199 | 396,962 | 725,690 |
| West Bengal | Darjiling | 405,156 | 1,515,637 | 380,695 | 681,981 |
| West Bengal | Haora | 1,221,345 | 3,831,426 | 1,150,235 | 1,891,566 |
| West Bengal | Hugli | 1,460,086 | 7,216,378 | 1,372,307 | 2,990,598 |
| West Bengal | Jalpaiguri | 1,013,274 | 3,864,389 | 952,236 | 1,715,965 |
| West Bengal | Koch Bihar | 808,878 | 2,439,220 | 760,937 | 1,222,031 |
| West Bengal | Kolkata | 723,229 | 2,268,808 | 681,081 | 1,120,489 |
| West Bengal | Maldah | 1,477,367 | 4,455,090 | 1,393,473 | 2,281,102 |
| West Bengal | Murshidabad | 3,104,112 | 10,246,883 | 2,919,468 | 5,628,774 |
| West Bengal | Nadia | 1,577,160 | 5,899,950 | 1,481,919 | 2,654,591 |
| West Bengal | North Twenty Four Parganas | 1,804,340 | 6,581,010 | 1,695,850 | 3,059,699 |
| West Bengal | Paschim Medinipur | 1,735,512 | 6,618,838 | 1,630,709 | 2,933,773 |
| West Bengal | Purba Medinipur | 1,225,174 | 4,672,527 | 1,153,236 | 2,112,871 |
| West Bengal | Puruliya | 1,047,693 | 5,292,708 | 985,052 | 2,125,090 |
| West Bengal | South Twenty Four Parganas | 2,833,463 | 10,599,620 | 2,664,848 | 4,819,516 |
| West Bengal | Uttar Dinajpur | 1,175,753 | 3,545,554 | 1,114,613 | 1,876,445 |
| India |  | 354,917,164 | 1,466,697,524 | 335,620,801 | 733,005,419 |

Table A11: Estimated cost (2020 US$) of 90% coverage of BCG, OPV, IPV, pentavalent, hepatitis B, RVV, and PCV vaccines among 12- to 23-month-old Indian children, by district

| State | District | Cost of 90% coverage, vaccine only | Cost of 90% coverage, vaccine and delivery | Incremental cost, current coverage to 90%, vaccine only | Incremental cost, current coverage to 90%, vaccine and delivery |
| --- | --- | --- | --- | --- | --- |
| Andaman and Nicobar Islands | Nicobars | 7,707 | 18,533 | 7,394 | 14,317 |
| Andaman and Nicobar Islands | North and Middle Andaman | 32,057 | 84,841 | 29,883 | 51,701 |
| Andaman and Nicobar Islands | South Andaman | 75,003 | 138,649 | 70,274 | 106,535 |
| Andhra Pradesh | Anantapur | 1,695,506 | 7,197,493 | 1,580,175 | 3,974,468 |
| Andhra Pradesh | Chittoor | 1,700,127 | 4,963,344 | 1,584,930 | 2,586,846 |
| Andhra Pradesh | East Godavari | 1,784,100 | 7,573,578 | 1,669,591 | 4,343,752 |
| Andhra Pradesh | Guntur | 1,628,576 | 6,913,372 | 1,520,331 | 3,899,072 |
| Andhra Pradesh | Krishna | 1,435,364 | 7,160,954 | 1,338,258 | 3,970,631 |
| Andhra Pradesh | Kurnool | 1,958,630 | 3,620,694 | 1,826,239 | 2,753,816 |
| Andhra Pradesh | Prakasam | 1,178,447 | 3,330,140 | 1,099,418 | 2,227,929 |
| Andhra Pradesh | Sri Potti Sriramulu Nellore | 860,243 | 3,651,768 | 811,057 | 2,211,647 |
| Andhra Pradesh | Srikakulam | 811,354 | 3,444,232 | 757,379 | 1,931,338 |
| Andhra Pradesh | Visakhapatnam | 1,077,650 | 4,574,670 | 1,004,811 | 2,542,358 |
| Andhra Pradesh | Vizianagaram | 864,791 | 3,671,076 | 806,761 | 2,053,307 |
| Andhra Pradesh | West Godavari | 1,080,098 | 2,540,654 | 1,007,053 | 1,729,344 |
| Andhra Pradesh | Y.S.R. | 1,329,222 | 3,196,224 | 1,239,196 | 2,156,642 |
| Arunachal Pradesh | Anjaw | 6,634 | 28,162 | 6,340 | 19,061 |
| Arunachal Pradesh | Changlang | 41,854 | 177,672 | 39,352 | 108,212 |
| Arunachal Pradesh | Dibang Valley | 1,305 | 5,541 | 1,232 | 3,385 |
| Arunachal Pradesh | East Kameng | 32,882 | 139,585 | 32,354 | 114,126 |
| Arunachal Pradesh | East Siang | 12,712 | 53,962 | 11,990 | 33,380 |
| Arunachal Pradesh | Kurung Kumey | 33,437 | 141,940 | 32,366 | 104,143 |
| Arunachal Pradesh | Lohit | 50,267 | 213,385 | 47,648 | 136,096 |
| Arunachal Pradesh | Lower Dibang Valley | 15,301 | 64,954 | 14,525 | 41,659 |
| Arunachal Pradesh | Lower Subansiri | 18,052 | 76,633 | 17,223 | 51,140 |
| Arunachal Pradesh | Papumpare | 64,595 | 274,208 | 61,256 | 174,032 |
| Arunachal Pradesh | Tawang | 9,822 | 41,695 | 9,447 | 29,520 |
| Arunachal Pradesh | Tirap | 24,341 | 75,719 | 23,193 | 48,931 |
| Arunachal Pradesh | Upper Siang | 2,209 | 5,311 | 2,097 | 3,871 |
| Arunachal Pradesh | Upper Subansiri | 24,526 | 104,115 | 23,672 | 75,209 |
| Arunachal Pradesh | West Kameng | 22,205 | 94,260 | 21,325 | 65,955 |
| Arunachal Pradesh | West Siang | 21,935 | 93,117 | 20,859 | 61,206 |
| Assam | Baksa | 372,264 | 950,718 | 348,794 | 610,593 |
| Assam | Barpeta | 647,923 | 2,750,462 | 617,833 | 1,857,764 |
| Assam | Bongaigaon | 242,709 | 685,863 | 228,345 | 492,824 |
| Assam | Cachar | 632,436 | 1,520,744 | 598,300 | 1,114,124 |
| Assam | Chirang | 207,363 | 880,264 | 196,374 | 557,929 |
| Assam | Darrang | 358,621 | 1,522,360 | 340,450 | 984,791 |
| Assam | Dhemaji | 275,359 | 1,168,910 | 258,478 | 697,451 |
| Assam | Dhubri | 1,041,278 | 4,420,266 | 1,008,625 | 3,279,919 |
| Assam | Dibrugarh | 408,111 | 1,080,084 | 381,103 | 668,545 |
| Assam | Dima Hasao | 78,451 | 333,029 | 73,563 | 195,358 |
| Assam | Goalpara | 414,763 | 997,331 | 391,491 | 721,815 |
| Assam | Golaghat | 377,214 | 907,042 | 352,517 | 623,916 |
| Assam | Hailakandi | 365,519 | 1,551,644 | 348,464 | 1,036,256 |
| Assam | Jorhat | 384,602 | 982,228 | 359,240 | 620,228 |
| Assam | Kamrup | 480,664 | 1,155,797 | 457,439 | 867,515 |
| Assam | Kamrup Metropolitan | 411,476 | 1,050,859 | 384,016 | 658,020 |
| Assam | Karbi Anglong | 292,166 | 1,240,255 | 279,630 | 853,455 |
| Assam | Karimganj | 712,008 | 1,712,083 | 673,515 | 1,246,315 |
| Assam | Kokrajhar | 326,353 | 1,628,156 | 308,159 | 1,025,266 |
| Assam | Lakhimpur | 388,758 | 1,650,293 | 364,943 | 973,843 |
| Assam | Morigaon | 419,487 | 1,780,738 | 394,724 | 1,087,560 |
| Assam | Nagaon | 1,369,693 | 3,870,578 | 1,298,524 | 2,887,353 |
| Assam | Nalbari | 281,878 | 1,196,585 | 264,785 | 712,209 |
| Assam | Sivasagar | 302,972 | 856,160 | 283,401 | 588,114 |
| Assam | Sonitpur | 627,304 | 3,129,587 | 593,766 | 2,019,670 |
| Assam | Tinsukia | 469,115 | 1,241,533 | 439,317 | 785,785 |
| Assam | Udalguri | 276,676 | 665,291 | 259,407 | 466,130 |
| Bihar | Araria | 1,898,497 | 4,565,094 | 1,786,786 | 3,228,162 |
| Bihar | Arwal | 403,202 | 948,429 | 376,073 | 648,828 |
| Bihar | Aurangabad | 975,990 | 3,036,048 | 910,527 | 1,736,334 |
| Bihar | Banka | 1,166,588 | 2,805,158 | 1,090,613 | 1,921,769 |
| Bihar | Begusarai | 1,878,784 | 5,249,556 | 1,752,388 | 3,169,639 |
| Bihar | Bhagalpur | 2,317,399 | 6,067,484 | 2,165,800 | 3,660,772 |
| Bihar | Bhojpur | 1,358,913 | 4,299,510 | 1,270,960 | 2,460,191 |
| Bihar | Buxar | 812,171 | 3,341,079 | 760,742 | 1,749,377 |
| Bihar | Darbhanga | 2,834,341 | 8,816,885 | 2,667,902 | 5,321,721 |
| Bihar | Gaya | 3,003,927 | 9,344,422 | 2,810,540 | 5,447,562 |
| Bihar | Gopalganj | 1,268,678 | 3,946,521 | 1,187,251 | 2,290,156 |
| Bihar | Jamui | 986,738 | 4,922,781 | 933,162 | 3,113,615 |
| Bihar | Jehanabad | 531,751 | 2,187,500 | 496,583 | 1,130,258 |
| Bihar | Kaimur (Bhabua) | 764,325 | 1,951,994 | 713,953 | 1,228,310 |
| Bihar | Katihar | 1,639,816 | 3,857,246 | 1,533,768 | 2,683,460 |
| Bihar | Khagaria | 1,230,357 | 3,827,314 | 1,149,760 | 2,215,388 |
| Bihar | Kishanganj | 1,031,395 | 2,729,634 | 971,249 | 1,745,645 |
| Bihar | Lakhisarai | 474,220 | 1,241,618 | 445,684 | 760,812 |
| Bihar | Madhepura | 1,236,483 | 3,912,148 | 1,157,280 | 2,241,253 |
| Bihar | Madhubani | 2,641,200 | 8,216,076 | 2,486,730 | 4,966,078 |
| Bihar | Munger | 713,332 | 1,677,932 | 666,478 | 1,154,133 |
| Bihar | Muzaffarpur | 2,420,068 | 9,955,589 | 2,273,051 | 5,376,018 |
| Bihar | Nalanda | 1,312,460 | 4,152,535 | 1,227,495 | 2,381,712 |
| Bihar | Nawada | 1,097,209 | 3,471,495 | 1,027,477 | 2,007,263 |
| Bihar | Pashchim Champaran | 2,226,279 | 9,450,645 | 2,115,383 | 6,097,888 |
| Bihar | Patna | 2,733,071 | 6,428,849 | 2,555,130 | 4,442,782 |
| Bihar | Purba Champaran | 3,603,433 | 9,434,619 | 3,417,659 | 6,036,752 |
| Bihar | Purnia | 2,196,427 | 6,949,348 | 2,050,256 | 3,940,930 |
| Bihar | Rohtas | 1,316,245 | 3,446,232 | 1,227,872 | 2,058,421 |
| Bihar | Saharsa | 1,228,763 | 3,251,978 | 1,147,565 | 2,011,905 |
| Bihar | Samastipur | 2,600,328 | 12,972,898 | 2,441,618 | 7,710,476 |
| Bihar | Saran | 2,215,860 | 10,978,760 | 2,084,389 | 6,318,702 |
| Bihar | Sheikhpura | 312,541 | 1,030,157 | 292,521 | 593,165 |
| Bihar | Sheohar | 492,448 | 1,558,074 | 461,955 | 910,401 |
| Bihar | Sitamarhi | 2,599,229 | 8,085,514 | 2,441,798 | 4,868,308 |
| Bihar | Siwan | 1,598,394 | 5,057,212 | 1,505,145 | 3,022,033 |
| Bihar | Supaul | 1,697,998 | 4,493,828 | 1,589,140 | 2,800,659 |
| Bihar | Vaishali | 2,171,831 | 5,747,850 | 2,031,504 | 3,571,414 |
| Chandigarh | Chandigarh | 287,061 | 908,241 | 267,479 | 507,334 |
| Chhattisgarh | Bastar | 715,339 | 1,720,091 | 666,653 | 1,156,770 |
| Chhattisgarh | Bijapur | 70,106 | 297,602 | 65,324 | 163,915 |
| Chhattisgarh | Bilaspur | 1,319,936 | 4,561,252 | 1,229,757 | 2,641,355 |
| Chhattisgarh | Dakshin Bastar Dantewada | 309,432 | 1,313,552 | 288,596 | 729,070 |
| Chhattisgarh | Dhamtari | 305,072 | 1,054,224 | 284,160 | 608,536 |
| Chhattisgarh | Durg | 1,235,282 | 3,490,748 | 1,150,750 | 2,297,465 |
| Chhattisgarh | Janjgir - Champa | 629,328 | 1,513,271 | 587,179 | 1,027,530 |
| Chhattisgarh | Jashpur | 368,688 | 1,565,097 | 345,159 | 906,696 |
| Chhattisgarh | Kabirdham | 418,011 | 1,005,142 | 390,698 | 691,621 |
| Chhattisgarh | Korba | 581,837 | 2,469,921 | 542,063 | 1,356,684 |
| Chhattisgarh | Korea (Koriya) | 311,899 | 1,324,023 | 291,179 | 742,209 |
| Chhattisgarh | Mahasamund | 545,671 | 1,593,028 | 508,543 | 829,378 |
| Chhattisgarh | Narayanpur | 48,700 | 206,733 | 45,554 | 118,182 |
| Chhattisgarh | Raigarh | 655,633 | 3,270,916 | 611,550 | 1,827,486 |
| Chhattisgarh | Raipur | 1,866,952 | 4,767,969 | 1,740,669 | 2,966,816 |
| Chhattisgarh | Rajnandgaon | 600,926 | 2,997,988 | 559,883 | 1,644,216 |
| Chhattisgarh | Surguja | 1,225,100 | 5,200,600 | 1,144,302 | 2,961,049 |
| Chhattisgarh | Uttar Bastar Kanker | 302,106 | 881,968 | 281,495 | 458,402 |
| Dadra and Nagar Haveli | Dadra and Nagar Haveli | 116,230 | 493,401 | 108,975 | 290,151 |
| Daman and Diu | Daman | 41,201 | 174,900 | 38,832 | 105,985 |
| Daman and Diu | Diu | 12,687 | 51,297 | 11,839 | 26,771 |
| Delhi | Central | 115,124 | 358,120 | 107,573 | 205,028 |
| Delhi | East | 475,564 | 1,258,601 | 444,711 | 775,710 |
| Delhi | New Delhi | 24,076 | 97,349 | 22,491 | 51,888 |
| Delhi | North | 281,320 | 1,403,491 | 263,611 | 802,638 |
| Delhi | North East | 994,833 | 2,540,684 | 932,732 | 1,628,807 |
| Delhi | North West | 1,041,588 | 1,925,463 | 971,910 | 1,465,857 |
| Delhi | South | 1,123,683 | 2,869,751 | 1,050,384 | 1,827,405 |
| Delhi | South West | 1,671,805 | 5,200,544 | 1,557,242 | 2,932,731 |
| Delhi | West | 542,901 | 2,689,872 | 506,416 | 1,451,784 |
| Goa | North Goa | 241,011 | 723,872 | 224,468 | 440,405 |
| Goa | South Goa | 225,501 | 1,033,495 | 210,023 | 568,400 |
| Gujarat | Ahmadabad | 2,319,748 | 6,139,319 | 2,177,749 | 3,886,616 |
| Gujarat | Amreli | 272,575 | 862,410 | 255,845 | 508,310 |
| Gujarat | Anand | 705,068 | 3,517,545 | 658,954 | 2,004,545 |
| Gujarat | Banaskantha | 1,300,380 | 6,487,528 | 1,237,384 | 4,317,896 |
| Gujarat | Bharuch | 595,712 | 1,884,794 | 557,857 | 1,087,934 |
| Gujarat | Bhavnagar | 618,000 | 1,922,434 | 583,640 | 1,178,585 |
| Gujarat | Dohad | 996,813 | 4,231,511 | 956,617 | 2,954,753 |
| Gujarat | Gandhinagar | 553,899 | 1,302,906 | 522,486 | 937,203 |
| Gujarat | Jamnagar | 759,407 | 2,362,314 | 708,332 | 1,349,558 |
| Gujarat | Junagadh | 580,346 | 1,395,490 | 543,810 | 970,964 |
| Gujarat | Kachchh | 644,251 | 1,880,824 | 606,343 | 1,021,252 |
| Gujarat | Kheda | 942,188 | 3,255,882 | 894,620 | 2,144,681 |
| Gujarat | Mahesana | 795,992 | 3,379,018 | 744,343 | 1,927,187 |
| Gujarat | Narmada | 247,680 | 632,544 | 231,411 | 396,963 |
| Gujarat | Navsari | 424,399 | 784,537 | 396,513 | 603,568 |
| Gujarat | Panchmahal | 787,575 | 3,929,170 | 754,556 | 2,757,464 |
| Gujarat | Patan | 413,875 | 2,064,798 | 394,834 | 1,407,386 |
| Gujarat | Porbandar | 125,175 | 389,386 | 116,863 | 224,350 |
| Gujarat | Rajkot | 1,146,550 | 2,756,976 | 1,072,322 | 1,904,134 |
| Gujarat | Sabarkantha | 942,645 | 4,001,566 | 883,241 | 2,304,457 |
| Gujarat | Surat | 2,087,638 | 8,862,110 | 1,963,268 | 5,237,364 |
| Gujarat | Surendranagar | 624,303 | 1,468,512 | 594,005 | 1,101,216 |
| Gujarat | Tapi | 239,273 | 1,015,722 | 223,211 | 565,503 |
| Gujarat | The Dangs | 97,629 | 414,440 | 91,674 | 244,866 |
| Gujarat | Vadodara | 1,409,230 | 4,383,740 | 1,320,032 | 2,592,600 |
| Gujarat | Valsad | 541,675 | 1,418,230 | 507,166 | 867,037 |
| Haryana | Ambala | 351,868 | 846,096 | 327,716 | 565,689 |
| Haryana | Bhiwani | 720,592 | 1,732,723 | 674,939 | 1,200,576 |
| Haryana | Faridabad | 1,060,542 | 4,502,046 | 1,003,918 | 2,802,598 |
| Haryana | Fatehabad | 450,079 | 1,400,078 | 419,261 | 790,034 |
| Haryana | Gurgaon | 842,720 | 3,577,382 | 796,313 | 2,211,487 |
| Haryana | Hisar | 567,960 | 1,450,501 | 529,858 | 898,470 |
| Haryana | Jhajjar | 351,817 | 1,493,476 | 330,176 | 861,972 |
| Haryana | Jind | 642,552 | 2,727,662 | 598,448 | 1,491,715 |
| Haryana | Kaithal | 451,726 | 1,917,595 | 420,947 | 1,057,358 |
| Haryana | Karnal | 753,090 | 1,923,300 | 701,415 | 1,185,148 |
| Haryana | Kurukshetra | 387,887 | 717,042 | 361,513 | 543,817 |
| Haryana | Mahendragarh | 314,968 | 1,337,053 | 293,581 | 739,850 |
| Haryana | Mewat | 975,043 | 4,139,096 | 946,861 | 3,088,690 |
| Haryana | Palwal | 552,283 | 2,344,466 | 529,459 | 1,621,488 |
| Haryana | Panchkula | 191,388 | 506,518 | 178,251 | 306,376 |
| Haryana | Panipat | 331,320 | 936,267 | 309,106 | 622,285 |
| Haryana | Rewari | 328,804 | 1,395,786 | 309,681 | 844,361 |
| Haryana | Rohtak | 660,031 | 1,587,100 | 616,714 | 1,085,790 |
| Haryana | Sirsa | 499,833 | 1,308,678 | 466,150 | 781,635 |
| Haryana | Sonipat | 485,391 | 2,060,506 | 453,194 | 1,152,911 |
| Haryana | Yamunanagar | 304,709 | 732,698 | 283,794 | 489,872 |
| Himachal Pradesh | Bilaspur | 121,889 | 385,650 | 114,084 | 225,453 |
| Himachal Pradesh | Chamba | 186,360 | 766,643 | 174,713 | 411,280 |
| Himachal Pradesh | Hamirpur | 186,255 | 526,332 | 175,134 | 368,676 |
| Himachal Pradesh | Kangra | 465,082 | 1,446,747 | 433,838 | 820,676 |
| Himachal Pradesh | Kinnaur | 18,290 | 43,979 | 17,056 | 29,767 |
| Himachal Pradesh | Kullu | 156,948 | 369,180 | 146,528 | 254,525 |
| Himachal Pradesh | Lahul and Spiti | 6,293 | 26,713 | 5,888 | 15,457 |
| Himachal Pradesh | Mandi | 381,130 | 997,887 | 355,510 | 596,916 |
| Himachal Pradesh | Shimla | 225,018 | 711,941 | 209,768 | 399,720 |
| Himachal Pradesh | Sirmaur | 150,424 | 393,844 | 140,323 | 235,882 |
| Himachal Pradesh | Solan | 205,893 | 640,478 | 192,386 | 373,669 |
| Himachal Pradesh | Una | 227,505 | 935,903 | 212,877 | 486,543 |
| Jammu and Kashmir | Anantnag | 360,953 | 1,247,332 | 336,351 | 723,823 |
| Jammu and Kashmir | Badgam | 229,934 | 715,265 | 214,170 | 403,163 |
| Jammu and Kashmir | Bandipore | 133,393 | 320,754 | 124,647 | 218,618 |
| Jammu and Kashmir | Baramula | 333,729 | 943,076 | 310,904 | 620,884 |
| Jammu and Kashmir | Doda | 153,146 | 432,771 | 145,148 | 316,955 |
| Jammu and Kashmir | Ganderbal | 118,162 | 408,327 | 110,052 | 235,417 |
| Jammu and Kashmir | Jammu | 435,793 | 1,217,661 | 406,672 | 731,894 |
| Jammu and Kashmir | Kargil | 38,923 | 165,230 | 36,254 | 90,453 |
| Jammu and Kashmir | Kathua | 218,449 | 691,157 | 203,454 | 384,182 |
| Jammu and Kashmir | Kishtwar | 111,749 | 295,748 | 104,358 | 181,426 |
| Jammu and Kashmir | Kulgam | 180,474 | 477,634 | 168,087 | 288,905 |
| Jammu and Kashmir | Kupwara | 269,250 | 712,584 | 251,022 | 434,701 |
| Jammu and Kashmir | Leh | 36,314 | 154,154 | 33,826 | 84,454 |
| Jammu and Kashmir | Pulwama | 167,040 | 392,919 | 155,575 | 265,106 |
| Jammu and Kashmir | Punch | 267,634 | 683,504 | 249,264 | 421,082 |
| Jammu and Kashmir | Rajouri | 329,147 | 1,397,241 | 310,293 | 835,130 |
| Jammu and Kashmir | Ramban | 140,580 | 397,261 | 132,258 | 277,140 |
| Jammu and Kashmir | Reasi | 154,999 | 657,977 | 144,854 | 367,733 |
| Jammu and Kashmir | Samba | 116,600 | 494,970 | 109,702 | 297,206 |
| Jammu and Kashmir | Shupiyan | 100,972 | 428,630 | 94,127 | 236,884 |
| Jammu and Kashmir | Srinagar | 336,960 | 1,048,192 | 313,831 | 590,293 |
| Jammu and Kashmir | Udhampur | 200,665 | 624,215 | 187,150 | 353,076 |
| Jharkhand | Bokaro | 665,800 | 2,826,349 | 623,313 | 1,651,410 |
| Jharkhand | Chatra | 724,258 | 3,074,504 | 685,898 | 1,950,053 |
| Jharkhand | Deoghar | 905,245 | 2,176,736 | 847,464 | 1,505,593 |
| Jharkhand | Dhanbad | 954,683 | 4,052,669 | 894,113 | 2,367,606 |
| Jharkhand | Dumka | 576,175 | 2,445,887 | 538,113 | 1,385,585 |
| Jharkhand | Garhwa | 898,721 | 3,815,106 | 846,209 | 2,329,058 |
| Jharkhand | Giridih | 1,365,320 | 5,795,838 | 1,280,937 | 3,407,880 |
| Jharkhand | Godda | 686,929 | 2,916,040 | 643,130 | 1,678,168 |
| Jharkhand | Gumla | 423,624 | 1,798,303 | 397,440 | 1,060,527 |
| Jharkhand | Hazaribagh | 925,795 | 2,226,152 | 863,547 | 1,509,718 |
| Jharkhand | Jamtara | 515,543 | 2,188,500 | 484,052 | 1,285,829 |
| Jharkhand | Khunti | 208,405 | 884,687 | 194,466 | 497,499 |
| Jharkhand | Kodarma | 397,244 | 1,981,831 | 371,078 | 1,120,581 |
| Jharkhand | Latehar | 308,821 | 1,310,960 | 291,279 | 812,337 |
| Jharkhand | Lohardaga | 189,703 | 805,296 | 177,606 | 460,070 |
| Jharkhand | Pakur | 495,275 | 2,102,462 | 462,500 | 1,193,638 |
| Jharkhand | Palamu | 1,128,784 | 4,791,734 | 1,061,761 | 2,892,582 |
| Jharkhand | Pashchimi Singhbhum | 596,019 | 2,530,128 | 562,331 | 1,558,691 |
| Jharkhand | Purbi Singhbhum | 778,669 | 3,305,484 | 726,501 | 1,855,450 |
| Jharkhand | Ramgarh | 344,433 | 1,462,133 | 321,883 | 838,695 |
| Jharkhand | Ranchi | 1,163,450 | 4,938,892 | 1,087,072 | 2,810,838 |
| Jharkhand | Sahibganj | 528,077 | 2,241,708 | 495,922 | 1,325,565 |
| Jharkhand | Saraikela Kharsawan | 622,843 | 2,643,995 | 582,127 | 1,521,027 |
| Jharkhand | Simdega | 253,761 | 1,077,225 | 237,875 | 634,009 |
| Karnataka | Bagalkot | 789,470 | 2,230,941 | 735,679 | 1,473,452 |
| Karnataka | Bangalore | 4,233,974 | 10,813,058 | 3,988,623 | 7,075,193 |
| Karnataka | Bangalore Rural | 307,693 | 1,306,171 | 287,530 | 731,372 |
| Karnataka | Belgaum | 1,775,703 | 4,534,930 | 1,657,357 | 2,819,852 |
| Karnataka | Bellary | 1,118,934 | 4,603,030 | 1,043,198 | 2,331,277 |
| Karnataka | Bidar | 536,818 | 1,290,824 | 502,038 | 881,086 |
| Karnataka | Bijapur | 691,556 | 1,766,151 | 650,672 | 1,137,242 |
| Karnataka | Chamarajanagar | 316,007 | 1,341,463 | 295,630 | 755,744 |
| Karnataka | Chikkaballapura | 319,713 | 1,357,195 | 299,148 | 762,138 |
| Karnataka | Chikmagalur | 281,705 | 1,195,848 | 263,820 | 683,686 |
| Karnataka | Chitradurga | 477,201 | 1,147,470 | 448,146 | 789,737 |
| Karnataka | Dakshina Kannada | 515,886 | 2,573,727 | 481,641 | 1,450,797 |
| Karnataka | Davanagere | 803,883 | 3,306,984 | 748,836 | 1,660,326 |
| Karnataka | Dharwad | 576,568 | 2,876,469 | 538,260 | 1,602,757 |
| Karnataka | Gadag | 335,919 | 790,164 | 315,314 | 546,695 |
| Karnataka | Gulbarga | 984,176 | 2,873,200 | 918,965 | 1,506,198 |
| Karnataka | Hassan | 529,351 | 2,247,116 | 493,759 | 1,242,460 |
| Karnataka | Haveri | 520,336 | 1,798,106 | 486,505 | 1,076,518 |
| Karnataka | Kodagu | 203,595 | 864,270 | 190,148 | 480,240 |
| Karnataka | Kolar | 506,154 | 1,217,090 | 471,685 | 818,195 |
| Karnataka | Koppal | 515,551 | 1,603,742 | 480,862 | 916,578 |
| Karnataka | Mandya | 680,726 | 2,889,710 | 636,206 | 1,633,263 |
| Karnataka | Mysore | 1,175,545 | 4,990,236 | 1,108,428 | 2,999,865 |
| Karnataka | Raichur | 549,743 | 1,739,349 | 514,165 | 997,362 |
| Karnataka | Ramanagara | 324,453 | 1,377,314 | 303,233 | 779,038 |
| Karnataka | Shimoga | 479,336 | 2,034,802 | 450,795 | 1,179,765 |
| Karnataka | Tumkur | 661,414 | 2,807,731 | 618,233 | 1,574,171 |
| Karnataka | Udupi | 257,348 | 814,232 | 240,217 | 460,982 |
| Karnataka | Uttara Kannada | 449,094 | 1,397,012 | 418,897 | 798,015 |
| Karnataka | Yadgir | 480,910 | 1,944,513 | 450,771 | 1,061,840 |
| Kerala | Alappuzha | 358,602 | 1,643,512 | 334,316 | 918,274 |
| Kerala | Ernakulam | 551,402 | 1,558,192 | 515,109 | 1,046,892 |
| Kerala | Idukki | 230,493 | 542,175 | 214,672 | 365,810 |
| Kerala | Kannur | 850,656 | 3,037,381 | 792,300 | 1,642,836 |
| Kerala | Kasaragod | 392,454 | 1,241,699 | 365,972 | 700,046 |
| Kerala | Kollam | 564,831 | 1,328,621 | 526,171 | 898,178 |
| Kerala | Kottayam | 614,848 | 2,026,581 | 572,645 | 1,123,527 |
| Kerala | Kozhikode | 870,831 | 3,009,296 | 813,700 | 1,777,116 |
| Kerala | Malappuram | 1,316,836 | 3,166,442 | 1,231,282 | 2,165,603 |
| Kerala | Palakkad | 967,986 | 3,062,641 | 903,136 | 1,736,862 |
| Kerala | Pathanamthitta | 310,552 | 1,538,667 | 289,290 | 813,002 |
| Kerala | Thiruvananthapuram | 779,671 | 1,441,288 | 726,629 | 1,088,651 |
| Kerala | Thrissur | 621,611 | 1,966,736 | 579,313 | 1,101,200 |
| Kerala | Wayanad | 234,325 | 1,169,035 | 218,675 | 647,584 |
| Lakshadweep | Lakshadweep | 24,705 | 80,964 | 23,013 | 45,398 |
| Madhya Pradesh | Alirajpur | 432,578 | 1,836,314 | 413,032 | 1,224,703 |
| Madhya Pradesh | Anuppur | 324,989 | 1,379,592 | 303,820 | 791,022 |
| Madhya Pradesh | Ashoknagar | 322,075 | 1,367,220 | 305,563 | 863,850 |
| Madhya Pradesh | Balaghat | 849,296 | 3,605,296 | 792,609 | 2,021,494 |
| Madhya Pradesh | Barwani | 925,592 | 3,929,175 | 878,805 | 2,517,263 |
| Madhya Pradesh | Betul | 715,894 | 3,039,000 | 667,754 | 1,698,209 |
| Madhya Pradesh | Bhind | 829,515 | 4,138,408 | 776,631 | 2,385,952 |
| Madhya Pradesh | Bhopal | 985,471 | 2,369,647 | 919,151 | 1,604,418 |
| Madhya Pradesh | Burhanpur | 349,556 | 1,483,881 | 329,373 | 893,507 |
| Madhya Pradesh | Chhatarpur | 771,408 | 3,274,661 | 728,600 | 1,992,618 |
| Madhya Pradesh | Chhindwara | 1,066,141 | 4,525,811 | 994,836 | 2,539,909 |
| Madhya Pradesh | Damoh | 568,698 | 2,414,146 | 533,312 | 1,402,178 |
| Madhya Pradesh | Datia | 325,510 | 1,623,953 | 305,256 | 946,400 |
| Madhya Pradesh | Dewas | 728,131 | 2,057,605 | 680,786 | 1,382,389 |
| Madhya Pradesh | Dhar | 1,039,913 | 2,500,558 | 970,444 | 1,691,034 |
| Madhya Pradesh | Dindori | 331,044 | 1,405,297 | 310,048 | 811,377 |
| Madhya Pradesh | Guna | 764,819 | 1,953,256 | 716,365 | 1,236,265 |
| Madhya Pradesh | Gwalior | 858,852 | 2,065,180 | 805,138 | 1,432,319 |
| Madhya Pradesh | Harda | 240,911 | 615,258 | 225,731 | 395,523 |
| Madhya Pradesh | Hoshangabad | 494,666 | 2,099,878 | 463,544 | 1,209,673 |
| Madhya Pradesh | Indore | 1,374,988 | 3,306,274 | 1,286,907 | 2,271,712 |
| Madhya Pradesh | Jabalpur | 966,676 | 2,324,453 | 901,680 | 1,574,874 |
| Madhya Pradesh | Jhabua | 601,941 | 2,555,265 | 570,994 | 1,644,959 |
| Madhya Pradesh | Katni | 562,322 | 2,387,080 | 525,784 | 1,367,517 |
| Madhya Pradesh | Khandwa (East Nimar) | 577,896 | 2,883,090 | 539,906 | 1,631,301 |
| Madhya Pradesh | Khargone (West Nimar) | 788,764 | 2,014,407 | 738,922 | 1,286,332 |
| Madhya Pradesh | Mandla | 399,997 | 1,698,005 | 373,548 | 958,783 |
| Madhya Pradesh | Mandsaur | 698,459 | 1,973,756 | 660,896 | 1,431,171 |
| Madhya Pradesh | Morena | 794,490 | 1,868,834 | 745,362 | 1,304,890 |
| Madhya Pradesh | Narsimhapur | 402,980 | 1,710,665 | 377,022 | 966,047 |
| Madhya Pradesh | Neemuch | 424,469 | 1,020,672 | 399,304 | 720,043 |
| Madhya Pradesh | Panna | 375,256 | 1,592,977 | 359,155 | 1,076,428 |
| Madhya Pradesh | Raisen | 644,383 | 1,881,210 | 600,356 | 976,752 |
| Madhya Pradesh | Rajgarh | 848,331 | 3,601,199 | 797,140 | 2,107,370 |
| Madhya Pradesh | Ratlam | 594,794 | 2,524,926 | 559,900 | 1,510,396 |
| Madhya Pradesh | Rewa | 1,063,464 | 4,514,448 | 995,545 | 2,577,753 |
| Madhya Pradesh | Sagar | 960,491 | 4,077,324 | 907,709 | 2,527,146 |
| Madhya Pradesh | Satna | 833,449 | 3,538,024 | 779,544 | 2,017,396 |
| Madhya Pradesh | Sehore | 492,378 | 1,183,964 | 460,030 | 805,006 |
| Madhya Pradesh | Seoni | 491,696 | 2,087,271 | 458,700 | 1,168,500 |
| Madhya Pradesh | Shahdol | 521,998 | 2,215,904 | 489,971 | 1,314,710 |
| Madhya Pradesh | Shajapur | 589,482 | 1,417,458 | 550,227 | 963,074 |
| Madhya Pradesh | Sheopur | 327,098 | 1,388,544 | 306,891 | 818,127 |
| Madhya Pradesh | Shivpuri | 956,291 | 2,299,482 | 896,149 | 1,589,843 |
| Madhya Pradesh | Sidhi | 653,225 | 2,772,969 | 619,578 | 1,771,082 |
| Madhya Pradesh | Singrauli | 729,429 | 3,096,455 | 688,041 | 1,905,835 |
| Madhya Pradesh | Tikamgarh | 596,774 | 2,533,328 | 565,409 | 1,592,522 |
| Madhya Pradesh | Ujjain | 885,615 | 2,502,636 | 828,812 | 1,712,322 |
| Madhya Pradesh | Umaria | 378,499 | 1,606,745 | 353,499 | 902,373 |
| Madhya Pradesh | Vidisha | 649,412 | 2,756,783 | 615,739 | 1,754,127 |
| Maharashtra | Ahmadnagar | 1,916,868 | 8,137,186 | 1,803,152 | 4,827,459 |
| Maharashtra | Akola | 651,965 | 2,767,620 | 612,271 | 1,622,758 |
| Maharashtra | Amravati | 975,155 | 4,139,574 | 914,228 | 2,420,479 |
| Maharashtra | Aurangabad | 1,180,470 | 5,011,144 | 1,103,319 | 2,849,332 |
| Maharashtra | Bhandara | 301,749 | 1,280,938 | 281,450 | 710,767 |
| Maharashtra | Bid | 1,163,847 | 2,798,566 | 1,091,200 | 1,942,316 |
| Maharashtra | Buldana | 860,994 | 2,070,330 | 808,043 | 1,443,370 |
| Maharashtra | Chandrapur | 609,106 | 2,585,680 | 568,221 | 1,447,944 |
| Maharashtra | Dhule | 812,323 | 4,052,637 | 761,495 | 2,373,537 |
| Maharashtra | Gadchiroli | 265,413 | 1,126,689 | 247,377 | 618,970 |
| Maharashtra | Gondiya | 461,097 | 1,957,378 | 430,579 | 1,102,329 |
| Maharashtra | Hingoli | 517,103 | 1,243,416 | 483,900 | 856,351 |
| Maharashtra | Jalgaon | 1,231,212 | 6,142,452 | 1,160,634 | 3,805,178 |
| Maharashtra | Jalna | 727,390 | 1,749,071 | 680,400 | 1,202,048 |
| Maharashtra | Kolhapur | 1,177,014 | 3,005,950 | 1,106,278 | 1,931,291 |
| Maharashtra | Latur | 1,041,568 | 5,196,324 | 974,262 | 2,983,918 |
| Maharashtra | Mumbai | 1,011,506 | 4,161,095 | 955,389 | 2,311,796 |
| Maharashtra | Mumbai Suburban | 2,759,018 | 6,634,286 | 2,611,064 | 4,812,214 |
| Maharashtra | Nagpur | 1,369,423 | 6,831,979 | 1,277,000 | 3,801,903 |
| Maharashtra | Nanded | 1,688,324 | 8,422,958 | 1,584,943 | 4,970,250 |
| Maharashtra | Nandurbar | 689,457 | 2,926,774 | 658,784 | 1,985,075 |
| Maharashtra | Nashik | 3,482,618 | 10,167,144 | 3,267,672 | 5,483,310 |
| Maharashtra | Osmanabad | 507,921 | 2,533,990 | 475,912 | 1,465,611 |
| Maharashtra | Parbhani | 773,429 | 3,858,595 | 723,498 | 2,231,544 |
| Maharashtra | Pune | 3,340,686 | 9,440,351 | 3,112,768 | 6,229,038 |
| Maharashtra | Raigarh | 1,190,092 | 5,051,992 | 1,111,642 | 2,875,613 |
| Maharashtra | Ratnagiri | 564,471 | 1,755,919 | 527,592 | 1,014,172 |
| Maharashtra | Sangli | 1,003,041 | 4,257,952 | 942,923 | 2,527,949 |
| Maharashtra | Satara | 1,246,487 | 3,638,990 | 1,169,802 | 1,944,863 |
| Maharashtra | Sindhudurg | 160,196 | 506,849 | 149,351 | 283,790 |
| Maharashtra | Solapur | 1,712,096 | 4,027,268 | 1,598,958 | 2,765,223 |
| Maharashtra | Thane | 4,971,532 | 12,696,690 | 4,700,220 | 8,431,361 |
| Maharashtra | Wardha | 311,868 | 1,555,895 | 291,108 | 863,572 |
| Maharashtra | Washim | 361,902 | 870,223 | 337,817 | 585,866 |
| Maharashtra | Yavatmal | 1,172,959 | 4,979,258 | 1,094,302 | 2,779,191 |
| Manipur | Bishnupur | 71,592 | 357,169 | 67,158 | 214,477 |
| Manipur | Chandel | 57,894 | 139,211 | 54,946 | 103,615 |
| Manipur | Churachandpur | 102,931 | 242,119 | 97,535 | 180,482 |
| Manipur | Imphal East | 189,697 | 502,041 | 178,042 | 321,157 |
| Manipur | Imphal West | 218,666 | 680,212 | 204,773 | 406,004 |
| Manipur | Senapati (excluding 3 subdivisions) | 87,270 | 161,327 | 82,517 | 129,953 |
| Manipur | Tamenglong | 58,267 | 107,712 | 55,434 | 87,932 |
| Manipur | Thoubal | 178,356 | 889,808 | 168,024 | 552,764 |
| Manipur | Ukhrul | 69,634 | 286,457 | 66,539 | 173,080 |
| Meghalaya | East Garo Hills | 198,368 | 476,992 | 189,861 | 364,280 |
| Meghalaya | East Khasi Hills | 395,915 | 1,209,506 | 372,334 | 716,229 |
| Meghalaya | Jaintia Hills | 247,445 | 743,198 | 232,731 | 488,715 |
| Meghalaya | Ribhoi | 136,244 | 550,890 | 128,658 | 321,616 |
| Meghalaya | South Garo Hills | 36,772 | 156,098 | 34,286 | 85,995 |
| Meghalaya | West Garo Hills | 231,582 | 654,420 | 218,398 | 466,648 |
| Meghalaya | West Khasi Hills | 231,816 | 937,326 | 217,821 | 534,304 |
| Mizoram | Aizawl | 145,102 | 586,705 | 137,484 | 343,948 |
| Mizoram | Champhai | 60,712 | 142,809 | 57,757 | 107,850 |
| Mizoram | Kolasib | 29,695 | 93,954 | 28,398 | 61,367 |
| Mizoram | Lawngtlai | 47,341 | 200,964 | 45,388 | 139,487 |
| Mizoram | Lunglei | 51,866 | 95,879 | 49,272 | 77,886 |
| Mizoram | Mamit | 33,189 | 140,890 | 31,726 | 96,107 |
| Mizoram | Saiha | 23,665 | 55,666 | 22,376 | 41,145 |
| Mizoram | Serchhip | 19,837 | 81,605 | 18,730 | 46,134 |
| Nagaland | Dimapur | 135,915 | 326,819 | 129,913 | 247,457 |
| Nagaland | Kiphire | 39,698 | 93,380 | 38,129 | 73,049 |
| Nagaland | Kohima | 57,880 | 153,181 | 54,500 | 98,063 |
| Nagaland | Longleng | 16,508 | 82,357 | 15,991 | 62,467 |
| Nagaland | Mokokchung | 39,408 | 110,110 | 37,091 | 70,564 |
| Nagaland | Mon | 122,000 | 379,510 | 118,594 | 272,931 |
| Nagaland | Peren | 31,770 | 81,136 | 30,291 | 55,858 |
| Nagaland | Phek | 62,979 | 175,972 | 59,901 | 116,146 |
| Nagaland | Tuensang | 83,005 | 219,676 | 79,010 | 149,190 |
| Nagaland | Wokha | 22,728 | 64,225 | 21,831 | 51,662 |
| Nagaland | Zunheboto | 47,266 | 163,334 | 45,390 | 116,438 |
| Odisha | Anugul | 593,432 | 1,426,956 | 552,750 | 954,921 |
| Odisha | Balangir | 710,974 | 3,547,010 | 662,173 | 1,933,613 |
| Odisha | Baleshwar | 669,364 | 1,891,538 | 624,175 | 1,251,148 |
| Odisha | Bargarh | 617,386 | 1,484,555 | 575,501 | 997,360 |
| Odisha | Baudh | 191,592 | 460,698 | 178,511 | 309,221 |
| Odisha | Bhadrak | 634,680 | 1,974,323 | 591,286 | 1,115,413 |
| Odisha | Cuttack | 733,541 | 1,920,578 | 684,574 | 1,154,866 |
| Odisha | Debagarh | 117,719 | 499,722 | 109,775 | 276,590 |
| Odisha | Dhenkanal | 464,984 | 1,973,877 | 433,399 | 1,091,948 |
| Odisha | Gajapati | 221,804 | 580,733 | 210,332 | 373,846 |
| Odisha | Ganjam | 1,224,733 | 3,206,634 | 1,146,618 | 1,939,745 |
| Odisha | Jagatsinghapur | 351,600 | 930,525 | 327,797 | 568,411 |
| Odisha | Jajapur | 621,025 | 1,148,018 | 578,616 | 868,660 |
| Odisha | Jharsuguda | 202,501 | 486,930 | 188,698 | 327,140 |
| Odisha | Kalahandi | 588,256 | 1,662,336 | 548,001 | 1,093,943 |
| Odisha | Kandhamal | 321,543 | 1,111,145 | 300,156 | 653,622 |
| Odisha | Kendrapara | 606,098 | 1,425,691 | 566,557 | 983,912 |
| Odisha | Kendujhar | 921,498 | 2,690,218 | 859,592 | 1,400,675 |
| Odisha | Khordha | 771,210 | 1,814,073 | 721,475 | 1,253,095 |
| Odisha | Koraput | 589,115 | 2,500,818 | 553,190 | 1,461,638 |
| Odisha | Malkangiri | 399,623 | 1,020,589 | 372,733 | 634,482 |
| Odisha | Mayurbhanj | 911,703 | 3,870,217 | 853,620 | 2,236,084 |
| Odisha | Nabarangapur | 623,175 | 2,645,404 | 581,496 | 1,478,868 |
| Odisha | Nayagarh | 333,671 | 974,119 | 310,835 | 505,331 |
| Odisha | Nuapada | 294,995 | 1,252,268 | 275,220 | 697,314 |
| Odisha | Puri | 578,460 | 1,069,333 | 538,767 | 807,008 |
| Odisha | Rayagada | 466,400 | 1,221,143 | 435,826 | 729,673 |
| Odisha | Sambalpur | 421,377 | 1,103,261 | 393,228 | 659,003 |
| Odisha | Subarnapur | 251,874 | 605,652 | 234,586 | 404,931 |
| Odisha | Sundargarh | 677,464 | 1,914,428 | 631,149 | 1,260,935 |
| Puducherry | Karaikal | 66,463 | 331,581 | 61,913 | 181,349 |
| Puducherry | Mahe | 8,832 | 43,761 | 8,226 | 23,051 |
| Puducherry | Puducherry | 283,494 | 1,203,446 | 264,036 | 658,146 |
| Puducherry | Yanam | 17,532 | 87,467 | 16,334 | 47,917 |
| Punjab | Amritsar | 779,806 | 2,178,874 | 726,280 | 1,296,493 |
| Punjab | Barnala | 139,775 | 640,602 | 130,181 | 352,317 |
| Punjab | Bathinda | 331,107 | 1,338,799 | 308,380 | 691,405 |
| Punjab | Faridkot | 235,783 | 658,807 | 219,599 | 392,009 |
| Punjab | Fatehgarh Sahib | 188,667 | 762,855 | 175,747 | 394,924 |
| Punjab | Firozpur | 707,305 | 1,871,916 | 658,882 | 1,132,936 |
| Punjab | Gurdaspur | 638,372 | 1,783,690 | 594,554 | 1,061,347 |
| Punjab | Hoshiarpur | 349,252 | 1,105,008 | 325,279 | 614,223 |
| Punjab | Jalandhar | 676,515 | 2,735,425 | 630,080 | 1,412,675 |
| Punjab | Kapurthala | 203,870 | 727,945 | 189,876 | 393,528 |
| Punjab | Ludhiana | 900,079 | 2,799,909 | 840,205 | 1,593,348 |
| Punjab | Mansa | 239,388 | 757,409 | 222,957 | 421,012 |
| Punjab | Moga | 257,620 | 1,041,664 | 239,938 | 537,954 |
| Punjab | Muktsar | 259,004 | 924,809 | 241,226 | 499,952 |
| Punjab | Patiala | 694,048 | 1,939,256 | 646,409 | 1,153,914 |
| Punjab | Rupnagar | 192,856 | 635,666 | 179,618 | 352,410 |
| Punjab | Sahibzada Ajit Singh Nagar | 363,392 | 1,494,909 | 338,449 | 748,844 |
| Punjab | Sangrur | 169,296 | 554,816 | 157,695 | 310,949 |
| Punjab | Shahid Bhagat Singh Nagar | 613,679 | 1,908,992 | 572,294 | 1,081,219 |
| Punjab | Tarn Taran | 309,452 | 1,251,240 | 288,212 | 646,187 |
| Rajasthan | Ajmer | 963,136 | 2,265,531 | 899,710 | 1,554,082 |
| Rajasthan | Alwar | 1,599,723 | 3,846,667 | 1,509,131 | 2,778,500 |
| Rajasthan | Banswara | 991,395 | 4,208,515 | 930,571 | 2,463,158 |
| Rajasthan | Baran | 560,207 | 1,430,700 | 522,676 | 891,271 |
| Rajasthan | Barmer | 1,251,264 | 5,311,665 | 1,197,329 | 3,583,902 |
| Rajasthan | Bharatpur | 1,530,600 | 4,050,802 | 1,450,186 | 2,682,741 |
| Rajasthan | Bhilwara | 1,091,633 | 3,084,816 | 1,018,955 | 2,070,401 |
| Rajasthan | Bikaner | 1,027,390 | 2,903,272 | 967,866 | 2,053,865 |
| Rajasthan | Bundi | 449,096 | 1,397,019 | 420,534 | 815,788 |
| Rajasthan | Chittaurgarh | 523,485 | 1,479,302 | 492,606 | 1,041,787 |
| Rajasthan | Churu | 780,175 | 3,892,251 | 730,779 | 2,254,406 |
| Rajasthan | Dausa | 652,017 | 1,567,829 | 610,922 | 1,088,961 |
| Rajasthan | Dhaulpur | 620,398 | 1,962,899 | 582,852 | 1,147,810 |
| Rajasthan | Dungarpur | 734,117 | 1,765,246 | 686,828 | 1,209,209 |
| Rajasthan | Ganganagar | 926,753 | 3,202,542 | 863,361 | 1,852,218 |
| Rajasthan | Hanumangarh | 780,983 | 3,315,304 | 730,975 | 1,938,214 |
| Rajasthan | Jaipur | 2,364,251 | 11,795,121 | 2,212,326 | 6,801,676 |
| Rajasthan | Jaisalmer | 366,408 | 1,827,990 | 348,549 | 1,193,474 |
| Rajasthan | Jalor | 1,028,862 | 2,420,135 | 982,195 | 1,826,989 |
| Rajasthan | Jhalawar | 453,982 | 1,067,877 | 423,135 | 724,264 |
| Rajasthan | Jhunjhunun | 855,157 | 2,056,297 | 798,340 | 1,397,634 |
| Rajasthan | Jodhpur | 1,554,046 | 7,753,053 | 1,465,237 | 4,778,254 |
| Rajasthan | Karauli | 683,749 | 1,790,213 | 643,114 | 1,110,191 |
| Rajasthan | Kota | 720,232 | 1,839,384 | 672,690 | 1,152,594 |
| Rajasthan | Nagaur | 1,260,922 | 5,352,664 | 1,190,090 | 3,298,497 |
| Rajasthan | Pali | 847,568 | 2,636,559 | 796,886 | 1,569,436 |
| Rajasthan | Pratapgarh | 432,045 | 1,834,048 | 403,906 | 1,033,570 |
| Rajasthan | Rajsamand | 496,617 | 918,039 | 465,037 | 707,887 |
| Rajasthan | Sawai Madhopur | 707,445 | 2,910,261 | 668,061 | 1,621,737 |
| Rajasthan | Sikar | 1,173,347 | 2,821,409 | 1,101,294 | 1,976,504 |
| Rajasthan | Sirohi | 616,530 | 1,450,229 | 584,100 | 1,063,688 |
| Rajasthan | Tonk | 558,330 | 1,766,518 | 520,545 | 993,302 |
| Rajasthan | Udaipur | 1,444,309 | 6,131,153 | 1,366,799 | 3,831,973 |
| Sikkim | East District | 58,772 | 166,083 | 54,774 | 109,867 |
| Sikkim | North District | 8,430 | 35,787 | 7,852 | 19,582 |
| Sikkim | South District | 30,895 | 131,152 | 28,782 | 72,000 |
| Sikkim | West District | 22,694 | 96,336 | 21,141 | 52,867 |
| Tamil Nadu | Ariyalur | 231,090 | 980,985 | 216,214 | 556,849 |
| Tamil Nadu | Chennai | 1,208,379 | 5,129,619 | 1,125,686 | 2,814,808 |
| Tamil Nadu | Coimbatore | 1,415,564 | 6,009,130 | 1,318,690 | 3,296,677 |
| Tamil Nadu | Cuddalore | 912,225 | 3,872,433 | 852,431 | 2,172,005 |
| Tamil Nadu | Dharmapuri | 598,060 | 2,538,789 | 559,877 | 1,449,339 |
| Tamil Nadu | Dindigul | 1,184,327 | 5,027,516 | 1,103,170 | 2,754,308 |
| Tamil Nadu | Erode | 1,152,212 | 3,363,763 | 1,073,206 | 1,741,892 |
| Tamil Nadu | Kancheepuram | 1,256,451 | 5,333,687 | 1,176,371 | 3,013,988 |
| Tamil Nadu | Kanniyakumari | 868,216 | 3,685,613 | 813,542 | 2,111,512 |
| Tamil Nadu | Karur | 549,876 | 1,322,224 | 512,133 | 884,021 |
| Tamil Nadu | Krishnagiri | 834,772 | 3,543,642 | 777,988 | 1,949,467 |
| Tamil Nadu | Madurai | 858,777 | 3,645,544 | 803,411 | 2,054,752 |
| Tamil Nadu | Nagapattinam | 610,338 | 2,590,912 | 578,555 | 1,623,111 |
| Tamil Nadu | Namakkal | 550,796 | 2,338,154 | 514,303 | 1,302,501 |
| Tamil Nadu | Perambalur | 239,845 | 1,018,150 | 224,115 | 573,476 |
| Tamil Nadu | Pudukkottai | 570,010 | 2,419,715 | 534,818 | 1,396,178 |
| Tamil Nadu | Ramanathapuram | 510,341 | 2,166,418 | 477,757 | 1,256,233 |
| Tamil Nadu | Salem | 1,862,357 | 7,905,783 | 1,736,726 | 4,369,820 |
| Tamil Nadu | Sivaganga | 405,223 | 2,021,638 | 378,094 | 1,126,779 |
| Tamil Nadu | Thanjavur | 962,190 | 2,313,664 | 896,581 | 1,554,210 |
| Tamil Nadu | The Nilgiris | 260,010 | 1,103,753 | 242,199 | 604,608 |
| Tamil Nadu | Theni | 475,388 | 2,018,043 | 445,394 | 1,161,136 |
| Tamil Nadu | Thiruvallur | 1,243,432 | 5,278,422 | 1,158,911 | 2,901,915 |
| Tamil Nadu | Thiruvarur | 621,061 | 2,636,428 | 579,599 | 1,475,068 |
| Tamil Nadu | Thoothukkudi | 448,920 | 1,079,464 | 422,242 | 758,741 |
| Tamil Nadu | Tiruchirappalli | 1,064,695 | 4,519,674 | 994,479 | 2,546,308 |
| Tamil Nadu | Tirunelveli | 1,036,200 | 2,491,628 | 969,275 | 1,712,503 |
| Tamil Nadu | Tiruppur | 1,184,400 | 5,027,825 | 1,103,103 | 2,749,638 |
| Tamil Nadu | Tiruvannamalai | 958,217 | 4,067,670 | 896,089 | 2,299,837 |
| Tamil Nadu | Vellore | 2,020,146 | 8,575,603 | 1,884,334 | 4,743,154 |
| Tamil Nadu | Viluppuram | 1,456,601 | 6,183,333 | 1,359,250 | 3,415,305 |
| Tamil Nadu | Virudhunagar | 762,489 | 3,236,799 | 716,315 | 1,924,581 |
| Telangana | Adilabad | 889,022 | 1,643,432 | 830,607 | 1,255,500 |
| Telangana | Hyderabad | 2,853,498 | 12,113,216 | 2,660,436 | 6,697,948 |
| Telangana | Karimnagar | 1,294,644 | 5,495,820 | 1,205,784 | 3,005,692 |
| Telangana | Khammam | 1,052,985 | 2,531,990 | 981,836 | 1,708,178 |
| Telangana | Mahbubnagar | 1,445,794 | 6,137,453 | 1,351,764 | 3,524,971 |
| Telangana | Medak | 1,015,684 | 4,311,620 | 946,008 | 2,359,479 |
| Telangana | Nalgonda | 1,186,523 | 5,036,840 | 1,107,938 | 2,819,328 |
| Telangana | Nizamabad | 1,189,222 | 2,859,582 | 1,108,844 | 1,932,312 |
| Telangana | Rangareddy | 2,768,113 | 11,750,755 | 2,580,195 | 6,501,038 |
| Telangana | Warangal | 1,333,140 | 5,659,235 | 1,244,197 | 3,174,014 |
| Tripura | Dhalai | 109,277 | 463,884 | 103,805 | 305,304 |
| Tripura | North Tripura | 259,155 | 661,850 | 245,914 | 453,335 |
| Tripura | South Tripura | 215,430 | 550,182 | 203,891 | 370,771 |
| Tripura | West Tripura | 548,242 | 1,318,292 | 516,093 | 939,586 |
| Uttar Pradesh | Agra | 2,052,043 | 5,240,670 | 1,926,792 | 3,402,850 |
| Uttar Pradesh | Aligarh | 1,877,310 | 4,915,229 | 1,759,946 | 3,027,083 |
| Uttar Pradesh | Allahabad | 2,723,762 | 13,588,700 | 2,574,447 | 8,561,775 |
| Uttar Pradesh | Ambedkar Nagar | 1,143,974 | 4,856,218 | 1,075,843 | 2,915,510 |
| Uttar Pradesh | Auraiya | 594,277 | 2,522,732 | 565,846 | 1,671,271 |
| Uttar Pradesh | Azamgarh | 1,501,428 | 7,490,543 | 1,423,122 | 4,830,704 |
| Uttar Pradesh | Baghpat | 622,090 | 1,646,391 | 582,380 | 1,025,636 |
| Uttar Pradesh | Bahraich | 2,110,786 | 8,960,374 | 2,067,120 | 7,079,124 |
| Uttar Pradesh | Ballia | 1,417,381 | 3,408,210 | 1,341,658 | 2,463,371 |
| Uttar Pradesh | Balrampur | 1,144,784 | 2,752,729 | 1,113,666 | 2,256,203 |
| Uttar Pradesh | Banda | 511,866 | 2,172,891 | 481,954 | 1,301,403 |
| Uttar Pradesh | Bara Banki | 1,252,545 | 5,317,104 | 1,186,303 | 3,323,635 |
| Uttar Pradesh | Bareilly | 1,925,456 | 4,917,381 | 1,821,808 | 3,262,649 |
| Uttar Pradesh | Basti | 1,098,234 | 2,583,314 | 1,031,202 | 1,828,670 |
| Uttar Pradesh | Bijnor | 1,579,886 | 7,881,964 | 1,477,074 | 4,524,040 |
| Uttar Pradesh | Budaun | 2,050,774 | 10,231,199 | 1,938,188 | 6,399,906 |
| Uttar Pradesh | Bulandshahr | 1,613,248 | 5,574,839 | 1,516,993 | 3,496,754 |
| Uttar Pradesh | Chandauli | 992,620 | 4,213,713 | 932,516 | 2,523,622 |
| Uttar Pradesh | Chitrakoot | 462,031 | 1,961,340 | 432,271 | 1,120,926 |
| Uttar Pradesh | Deoria | 1,281,758 | 3,392,231 | 1,202,710 | 2,135,758 |
| Uttar Pradesh | Etah | 865,485 | 2,526,694 | 819,025 | 1,404,048 |
| Uttar Pradesh | Etawah | 649,732 | 2,758,140 | 613,365 | 1,681,069 |
| Uttar Pradesh | Faizabad | 878,488 | 3,729,216 | 828,593 | 2,265,736 |
| Uttar Pradesh | Farrukhabad | 864,890 | 2,079,699 | 821,538 | 1,529,597 |
| Uttar Pradesh | Fatehpur | 969,890 | 4,117,222 | 915,188 | 2,539,252 |
| Uttar Pradesh | Firozabad | 1,168,337 | 2,809,364 | 1,098,610 | 1,985,705 |
| Uttar Pradesh | Gautam Buddha Nagar | 1,010,706 | 4,290,490 | 949,833 | 2,537,814 |
| Uttar Pradesh | Ghaziabad | 2,078,380 | 8,822,809 | 1,956,263 | 5,360,767 |
| Uttar Pradesh | Ghazipur | 1,396,727 | 3,358,546 | 1,317,810 | 2,413,094 |
| Uttar Pradesh | Gonda | 1,479,572 | 4,181,082 | 1,414,590 | 3,219,635 |
| Uttar Pradesh | Gorakhpur | 1,933,282 | 6,680,766 | 1,809,823 | 4,066,745 |
| Uttar Pradesh | Hamirpur | 282,347 | 1,198,576 | 266,826 | 750,244 |
| Uttar Pradesh | Hardoi | 1,631,312 | 6,924,986 | 1,546,404 | 4,350,219 |
| Uttar Pradesh | Jalaun | 510,296 | 2,166,228 | 481,794 | 1,333,415 |
| Uttar Pradesh | Jaunpur | 1,919,991 | 5,425,649 | 1,813,535 | 3,943,919 |
| Uttar Pradesh | Jhansi | 658,341 | 1,583,036 | 616,551 | 1,094,703 |
| Uttar Pradesh | Jyotiba Phule Nagar | 882,629 | 4,403,388 | 827,307 | 2,594,196 |
| Uttar Pradesh | Kannauj | 737,240 | 1,772,756 | 697,693 | 1,284,072 |
| Uttar Pradesh | Kanpur Dehat | 749,708 | 3,182,540 | 704,826 | 1,887,792 |
| Uttar Pradesh | Kanpur Nagar | 1,779,297 | 7,553,188 | 1,672,812 | 4,488,326 |
| Uttar Pradesh | Kanshiram Nagar | 780,826 | 3,895,500 | 739,989 | 2,492,193 |
| Uttar Pradesh | Kaushambi | 656,882 | 2,788,494 | 622,274 | 1,758,399 |
| Uttar Pradesh | Kheri | 2,374,927 | 10,081,664 | 2,232,882 | 6,016,646 |
| Uttar Pradesh | Kushinagar | 1,773,892 | 6,129,969 | 1,681,091 | 3,999,390 |
| Uttar Pradesh | Lalitpur | 536,524 | 2,277,566 | 501,937 | 1,291,632 |
| Uttar Pradesh | Lucknow | 1,150,758 | 2,127,273 | 1,080,894 | 1,659,035 |
| Uttar Pradesh | Mahamaya Nagar | 761,810 | 2,152,778 | 718,552 | 1,537,349 |
| Uttar Pradesh | Mahoba | 288,525 | 693,783 | 270,679 | 481,648 |
| Uttar Pradesh | Mahrajganj | 1,130,436 | 5,639,686 | 1,067,189 | 3,517,770 |
| Uttar Pradesh | Mainpuri | 730,946 | 3,646,652 | 687,347 | 2,203,153 |
| Uttar Pradesh | Mathura | 1,397,410 | 3,287,048 | 1,318,018 | 2,371,547 |
| Uttar Pradesh | Mau | 1,011,109 | 2,431,296 | 954,794 | 1,759,326 |
| Uttar Pradesh | Meerut | 2,004,986 | 5,665,835 | 1,889,815 | 4,029,815 |
| Uttar Pradesh | Mirzapur | 1,235,474 | 5,244,636 | 1,161,363 | 3,120,972 |
| Uttar Pradesh | Moradabad | 2,305,531 | 6,730,760 | 2,180,958 | 3,769,794 |
| Uttar Pradesh | Muzaffarnagar | 2,472,121 | 6,985,898 | 2,327,640 | 4,917,500 |
| Uttar Pradesh | Pilibhit | 855,464 | 2,184,750 | 801,516 | 1,395,882 |
| Uttar Pradesh | Pratapgarh | 1,062,996 | 2,500,425 | 999,804 | 1,796,943 |
| Uttar Pradesh | Rae Bareli | 1,241,805 | 5,271,513 | 1,163,053 | 3,022,196 |
| Uttar Pradesh | Rampur | 1,234,277 | 2,967,920 | 1,156,545 | 2,041,536 |
| Uttar Pradesh | Saharanpur | 1,727,429 | 8,618,052 | 1,619,700 | 5,122,298 |
| Uttar Pradesh | Sant Kabir Nagar | 888,817 | 3,656,383 | 837,712 | 1,999,233 |
| Uttar Pradesh | Sant Ravidas Nagar (Bhadohi) | 911,036 | 2,574,472 | 863,117 | 1,900,613 |
| Uttar Pradesh | Shahjahanpur | 1,377,648 | 6,873,010 | 1,292,236 | 4,039,978 |
| Uttar Pradesh | Shrawasti | 752,110 | 3,192,737 | 728,518 | 2,340,932 |
| Uttar Pradesh | Siddharth Nagar | 1,173,861 | 3,714,018 | 1,120,240 | 2,387,041 |
| Uttar Pradesh | Sitapur | 2,416,590 | 10,258,524 | 2,278,350 | 6,170,886 |
| Uttar Pradesh | Sonbhadra | 1,002,465 | 4,255,507 | 951,101 | 2,726,073 |
| Uttar Pradesh | Sultanpur | 1,415,968 | 4,133,772 | 1,339,331 | 2,316,217 |
| Uttar Pradesh | Unnao | 1,282,498 | 3,083,873 | 1,207,904 | 2,164,062 |
| Uttar Pradesh | Varanasi | 1,482,768 | 3,565,439 | 1,391,602 | 2,493,917 |
| Uttarakhand | Almora | 181,200 | 563,664 | 169,482 | 328,571 |
| Uttarakhand | Bageshwar | 95,557 | 224,774 | 89,638 | 160,037 |
| Uttarakhand | Chamoli | 151,783 | 397,404 | 142,418 | 245,037 |
| Uttarakhand | Champawat | 90,844 | 373,711 | 84,915 | 195,160 |
| Uttarakhand | Dehradun | 664,525 | 1,697,117 | 620,620 | 1,069,927 |
| Uttarakhand | Garhwal | 221,688 | 566,164 | 207,294 | 359,192 |
| Uttarakhand | Hardwar | 1,001,239 | 4,250,300 | 943,378 | 2,580,512 |
| Uttarakhand | Nainital | 335,862 | 1,675,598 | 314,720 | 973,062 |
| Uttarakhand | Pithoragarh | 150,992 | 477,729 | 140,928 | 271,891 |
| Uttarakhand | Rudraprayag | 102,342 | 246,090 | 95,705 | 169,641 |
| Uttarakhand | Tehri Garhwal | 214,019 | 739,577 | 200,563 | 453,260 |
| Uttarakhand | Udham Singh Nagar | 640,498 | 2,718,943 | 601,867 | 1,621,812 |
| Uttarakhand | Uttarkashi | 122,049 | 293,477 | 114,040 | 202,331 |
| West Bengal | Bankura | 1,245,444 | 3,940,499 | 1,160,973 | 2,212,339 |
| West Bengal | Barddhaman | 2,841,761 | 9,366,638 | 2,654,556 | 5,374,609 |
| West Bengal | Birbhum | 1,496,904 | 4,656,474 | 1,395,531 | 2,651,767 |
| West Bengal | Dakshin Dinajpur | 597,086 | 1,889,139 | 558,333 | 1,091,717 |
| West Bengal | Darjiling | 573,685 | 1,784,581 | 535,772 | 1,036,283 |
| West Bengal | Haora | 1,729,376 | 4,576,870 | 1,616,667 | 2,839,174 |
| West Bengal | Hugli | 2,067,423 | 8,359,430 | 1,929,859 | 4,452,255 |
| West Bengal | Jalpaiguri | 1,434,754 | 4,539,467 | 1,339,230 | 2,587,126 |
| West Bengal | Koch Bihar | 1,145,338 | 2,925,055 | 1,069,652 | 1,848,759 |
| West Bengal | Kolkata | 1,024,063 | 2,710,229 | 956,826 | 1,678,504 |
| West Bengal | Maldah | 2,091,892 | 5,342,438 | 1,959,938 | 3,448,628 |
| West Bengal | Murshidabad | 4,395,296 | 12,420,544 | 4,102,411 | 8,363,777 |
| West Bengal | Nadia | 2,233,194 | 6,946,875 | 2,082,136 | 3,959,931 |
| West Bengal | North Twenty Four Parganas | 2,554,872 | 7,783,122 | 2,384,756 | 4,606,299 |
| West Bengal | Paschim Medinipur | 2,457,415 | 7,775,096 | 2,290,060 | 4,350,410 |
| West Bengal | Purba Medinipur | 1,734,796 | 5,488,780 | 1,621,424 | 3,171,921 |
| West Bengal | Puruliya | 1,483,490 | 6,102,730 | 1,384,595 | 3,145,262 |
| West Bengal | South Twenty Four Parganas | 4,012,069 | 12,480,483 | 3,746,566 | 7,237,424 |
| West Bengal | Uttar Dinajpur | 1,664,818 | 4,251,744 | 1,568,105 | 2,775,388 |
| India |  | 502,548,302 | 1,730,429,635 | 471,543,840 | 1,055,728,860 |
